# Agreement of an invasive angiography-derived computational model of coronary microvascular physiology against continuous thermodilution

**DOI:** 10.1371/journal.pdig.0001830

**Published:** 2026-11-13

**Authors:** Daniel J. Taylor, Tom Newman, Karolina Tlałka, Eron Yones, Kenneth Anigboro, Harry Saxton, Xu Xu, Alberto M. Biancardi, Rebecca Gosling, Krzysztof Czechowicz, Andrew Narracott, D. Rod Hose, Dominika Ciupek, Alessandro Maino, Federico Marin, Samer Fawaz, Ian Halliday, OxAMI Investigators, Julian P. Gunn, Giovanni Luigi De Maria, Thomas R. Keeble, Paul D. Morris

**Affiliations:** 1 Division of Clinical Medicine, School of Medicine and Population Health, University of Sheffield, Sheffield, United Kingdom; 2 Insigneo Institute for Healthcare Technology, Sheffield, United Kingdom; 3 NIHR Sheffield Biomedical Research Centre, Sheffield Teaching Hospitals NHS Foundation Trust, Sheffield, United Kingdom; 4 Department of Cardiology and Cardiothoracic Surgery, Sheffield Teaching Hospitals NHS Trust, Sheffield, United Kingdom; 5 Sano Centre for Computational Medicine, Cracow, Poland; 6 School of Computer Science, University of Sheffield, Sheffield, United Kingdom; 7 Oxford Heart Centre, Oxford University Hospitals, Headley Way, United Kingdom; 8 Anglia Ruskin School of medicine and MTRC, Chelmsford, Essex, United Kingdom; 9 Essex Cardiothoracic Centre, Basildon, Essex, United Kingdom; Nanjing University, CHINA

## Abstract

Coronary microvascular dysfunction is associated with myocardial ischaemia and an adverse prognosis, but accurate invasive assessment is technically challenging, requires dedicated hardware and increases procedure time and cost. Assessment can be performed with three-dimensional (3D) computational fluid dynamics (CFD) modelling, but this technique lacks robust *in vivo* validation. In this study, we compared the accuracy of a 3D CFD method against continuous thermodilution (CIT). Patients with acute and chronic coronary syndromes, undergoing CIT and angiography-derived assessment, were prospectively recruited from two tertiary cardiac centres. Invasive coronary angiographic images were used to reconstruct 3D anatomy *in silico*. Microvascular resistance reserve (MRR) and absolute flow were computed using 3D CFD in reconstructed coronary arteries. Invasive pressure measurements, recorded during fractional flow reserve assessment, informed the CFD boundary conditions. Paired flow results were available for 131 arteries from 89 patients. Median FFR was 0.81 [0.70 – 0.89] and 108 (81%) vessels contained an epicardial stenosis. Median computed MRR was 2.31 [1.83 – 3.00], which was significantly lower than CIT assessed MRR (2.79 [2.21 – 3.52], z = 3.57, p = 0.0004). There was evidence of a moderate relationship between computed and measured MRR (ρ = 0.58, p < 0.0001) and area under the receiver operator characteristic curve was 0.77 (95% CI 0.68–0.86), indicating fair classification. There was a moderate relationship between computed and measured hyperaemic vessel inlet flow (ρ = 0.44, p < 0.0001). For both MRR and flow, agreement improved at lower, more clinically relevant, values. In this clinical validation study, when compared with CIT, the novel CFD method demonstrated a moderate correlation, and fair diagnostic accuracy. This CFD method may be a useful, low-cost and less-invasive tool in the assessment of microvascular physiology that could complement current invasive techniques.

## Author summary

The coronary arteries feed the heart muscle with oxygen and nutrients which are vital to keep the heart pumping. With time, fatty deposits may narrow the coronary arteries, and this can give people chest pain (angina) or cause a heart attack. An invasive heart scan may aid doctors in decided how best to manage these patients. However, it is currently difficult to quantify the reduction in blood flow caused by a narrowing (i.e., 50 mL per minute lost), or whether there is disease in the arteries too small to see with the heart scan. In this study, we tested whether a computer simulation could predict flow down the coronary artery in mL/min and if it could predict whether a patient’s small arteries were healthy. We tested against a gold-standard invasive measurement, performed in only a few specialist heart centres. We compared the invasive and computer results in 89 patients. The two methods were moderately comparable, with the computer simulation achieving a correct diagnosis 63% of the time. The computer method is, therefore, not a universal replacement to invasive testing. It is best considered as a method to quickly select the patients who truly need the full invasive test.

## Introduction

In the invasive management of coronary artery disease (CAD), the use of intra-coronary physiological assessment is associated with improved clinical outcomes, reduced costs and is recommended in international guideline documents [1,2]. Pressure-based assessment of fractional flow reserve (FFR) is now widely adopted, to identify ischaemia causing lesions in the epicardial coronary arteries and to guide percutaneous coronary intervention (PCI) [3]. While FFR is influenced both by anatomy (stenosis severity) and physiology (coronary flow), it does not yield any focused assessment of the coronary microcirculation [4]. This is a significant limitation; microvascular dysfunction is a prognostically important cause of ischaemia and angina [5], especially in women [6]. To assess the coronary microcirculation, an estimation of coronary flow must be made. This adds technical complexity, time and cost to the procedure. Common techniques for assessing microvascular physiology are bolus thermodilution and Doppler. These estimate surrogates of coronary blood flow (mean transit time and flow velocity) to derive functional indices like coronary flow reserve (CFR) and microvascular resistance reserve (MRR). Abnormalities of these surrogates are associated with prognostically significant endpoints [7] and functional outcomes [8]. Yet, correlation and agreement between these techniques is often poor, even in expert hands [9]. Continuous infusion thermodilution (CIT) provides an alternative with more robust repeatability [10] and superior accuracy [11,12], but requires a dedicated catheter (Rayflow, Hexacath, France), and software module (Coroventis, Uppsala, Sweden) which quantifies flow at the location of an infusion microcatheter which also adds to procedure complexity, time and cost.

Computational fluid dynamics (CFD) modelling has been used to assess coronary physiology during routine angiography and FFR assessment using a system called virtuQ (University of Sheffield, UK) [13,14]. Thus far, however, clinical validation is limited to *in vitro* studies [13] and a small cohort of healthy vessels [15]. Given that the CFD model was developed for use in the context of CAD, the *in vivo* accuracy for this method remains unknown. The primary aim of this clinical validation study was to quantify the agreement of MRR between virtuQ and continuous thermodilution (CIT). Secondary aims included quantification of agreement between virtuQ and CIT for absolute flow at the vessel inlet, total vessel resistance and CFR along with identification of primary sources of error in the CFD workflow.

## Methods

### Ethics statement

Data collection for research purposes was approved by Regional Ethics Committees (OxAMI REC number 10/H0408/24, Essex-SAAMI REC reference 22/EE/0016), compliant with the Declaration of Helsinki and all patients gave written informed consent.

### Patient recruitment and clinical data collection

Clinical data for this multi-centre cohort study were sourced from the Oxford Heart Centre and the Essex Cardiothoracic Centre. This included adult patients undergoing clinically indicated invasive coronary angiography and coronary physiological assessment, who were prospectively enrolled into the OxAMI (Oxford Acute Myocardial Infarction) and Essex-SAAMI (Essex Stable Angina and Acute Myocardial Infarction) studies respectively. Patients presenting with either acute (ST-elevation myocardial infarction (STEMI), non-ST elevation myocardial infarction (NSTEMI)) or chronic coronary syndromes (CCS) were considered suitable for inclusion. Exclusion criteria included ostial left main stem disease, chronic total occlusion, history of coronary artery bypass grafting, severe renal impairment (estimated glomerular filtration rate <30 ml/min), contraindication to the induction of hyperaemia, hemodynamic or electrical instability, pregnancy or inability to consent.

Coronary angiograms were acquired following standard clinical protocols, using a 6 French guide catheter, with images optimised for computational reconstruction (minimum two views of vessel of interest separated by 30°). A thermo-pressure sensitive guidewire (PressureWire X, Abbott Vascular, Santa Clara, California) was placed in the guide catheter tip, the pressure equalised, and subsequently advanced into the distal coronary artery. The dedicated, CIT Rayflow microcatheter (Hexacath, Paris, France) was advanced over the guidewire and absolute coronary flow (mL/min), under both baseline and saline-induced hyperaemic conditions, assessed using the Coroventis (Coroflow, Uppsala, Sweden) system [16]. The 0.84mm diameter CIT microcatheter, measures absolute coronary flow by continuously infusing room temperature saline into the proximal coronary artery and computes flow at the location of infusion from downstream temperature dynamics. In patients presenting acutely, flow was recorded in both culprit and non-culprit vessels during the index angiogram. For baseline measurements, saline was infused at 10mL/min and for hyperaemic measurements, 20mL/min for LAD arteries and 15mL/min for non-LAD arteries. The CIT method is therefore dependent upon complete mixing of blood and saline infusate upstream to distal temperature measurement, which has been investigated in previous computational modelling studies [17]. Pseudonymised angiography (DICOM) and physiological data were exported to the University of Sheffield for offline computational processing and statistical analysis.

In addition to absolute flow, MRR, vessel resistance distal to the CIT microcatheter outlet and CFR were calculated using:


$$\begin{array}{c} MRR\;=\;\frac{CFR}{FFR}\;\times \;\frac{P_{aRest}}{P_{aHyp}} \end{array}$$ (1)

$$\begin{array}{c} \;R\mu \;=\;\frac{P_{d}}{Q}\; \end{array}$$ (2)

$$\begin{array}{c} \;CFR\;=\;\frac{Q_{Hyperaemia}}{Q_{Rest}} \end{array}$$ (3)

where Q represents flow, while P_a_ and P_d_ denote aortic and distal coronary pressure respectively and Rμ is vessel resistance. The subscripts _CIT_ and _Virtu_ are hereon used to denote haemodynamic values measured with the continuous infusion thermodilution method and computed with virtuQ respectively.

### Coronary reconstruction and flow simulation

The CFD workflow (virtuQ), including vessel reconstruction protocol, has been described previously [13,18]. In brief, two angiographic projections, acquired ≥ 30° apart, displaying the vessel of interest were selected. Cases without two projections clearly displaying the vessel and lesion(s) of interest or in surgically grafted vessels were excluded. End-diastole was selected manually, guided by the ECG, and table movement artefact was corrected for. The inlet and outlet of the arterial reconstruction were selected by the operator, corresponding to the location of the CIT infusion head (i.e., location of invasive flow measurement) and distal pressure measurement respectively. The vessel centreline and borders were traced semi-automatically from the imaged contrast gradient, with manual correction if required. Finally, a rigid, locally axisymmetric 3D reconstruction was generated automatically using an epipolar line method [18]. Specifically, epipolar lines were computed to establish point-to-point correspondence between projections, identifying corresponding 2D nodes along the paired centrelines. These matched points were then triangulated into 3D space and smoothed using parametric Hermite cubic spline interpolation to form the final 3D centreline and luminal surface geometry.

Anatomical reconstruction accuracy including reconstruction inlet and outlet placement, luminal border tracing and appropriate representation of vessel 3D structure was verified qualitatively by an interventional cardiologist against the original angiogram. All reconstructions and physiological simulations were performed blinded to invasive physiology.

Absolute coronary flow, during hyperaemia, was then computed from a CFD simulation using P_a_ and P_d_ values as static boundary conditions at the vessel inlet and outlet respectively. To account for side branch losses, taper of the reconstructed main vessel was used to infer branch magnitude and flow loss using the Huo-Kassab law [19]:


$$\begin{array}{c} {{Diameter}_{SB}}^{\frac{\text{7}}{\text{3}}}\;=\;{{Diameter}_{PV}}^{\frac{\text{7}}{\text{3}}}\;-\;{{Diameter}_{DV}}^{\frac{\text{7}}{\text{3}}} \end{array}$$ (4)

$$\begin{array}{c} \;Q\;\propto \;\;{Diameter}^{\text{7}/\text{3}} \end{array}$$ (5)

where PV and DV denote diameter of the reconstructed (main) vessel proximal and distal to the side branch (SB). The appropriateness of this scaling law for human coronary arteries has been previously demonstrated [20]. In simulations, side branch flow losses were distributed along the vessel length and were removed by ‘leaking’ flow through the arterial wall within the CFD simulation by modelling this as a porous medium [14]. All simulations included side branch flow and used standard parameters for blood viscosity μ = 0.0035 Pa.s and density ρ = 1050 kg/m^3^. Simulations considered only static and not dynamic or gravitational pressure losses.

### Statistical analysis

Categorical variables are presented as frequency (percentage). Continuous variables are presented as mean ±standard deviation or median [interquartile range] for normally distributed and skewed data respectively. Normality of data distribution was assessed using histograms and, where inconclusive, the Shapiro–Wilk test. Mean values were compared using t-tests and the Wilcoxon signed-rank test as appropriate. The relationship between haemodynamic variables was assessed with Spearman’s ρ and linear regression. Agreement was assessed with Bland Altman plots. Where data did not meet the parametric assumptions for the original Bland Altman method [21], we derived median bias and limits of agreement using quantile regression at the 50^th^, 2.5^th^ and 97.5^th^ centiles [22,23]. The diagnostic performance of CFR (prespecified threshold ≤2.5 [24]) and MRR (prespecified threshold ≤2.5 [25]) were quantified with receiver operating characteristic (ROC) curves and associated area under the curve (AUC) values computed using DeLong’s method. We calculated diagnostic accuracy, sensitivity, specificity, positive predictive value (PPV) and negative predictive value (NPV) with associated 95% CI computed using the Clopper-Pearson exact method and Locally Estimated Scatterplot Smoothing (LOESS) plots to visually display classification concordance. All statistical tests were two-tailed unless otherwise specified. Analyses were performed using RStudio version 2024.04.2 + 764, using the pROC, binom.test and ggplot2 packages.

We also performed an error propagation analysis, using regression and SHapley Additive exPlanations (SHAP) analysis, to assess predictors of agreement between CIT- and CFD-derived flow and MRR. This hypothesis-driven secondary analysis considered 13 variables likely to influence the physics underpinning CFD simulations. Details of included variables, inclusion rationale and further SHAP methodological details are given in the Text A and B in S1 Appendix. SHAP results are presented as a dimensionless value, with larger numbers denoting a greater overall contribution to model agreement.

## Results

### Study population

One hundred and two patients were included, in whom invasive physiology was assessed in 178 arteries. Thirteen patients and 43 vessels were excluded, primarily due to insufficient angiographic views for arterial reconstruction, leaving 89 patients and 135 vessels in the final analysis (see Fig 1 for exclusion flowchart and Table A in S1 Appendix for characteristics of excluded patients and vessels). Of the included patients, 70 (79%) were male and mean age was 63 ±11 years. The left anterior descending artery (LAD) was the most frequently included target vessel (n = 83 (62%)) and the median FFR was 0.81 [0.70 – 0.89] (see Figure A in S1 Appendix for full invasive FFR data). Of the reconstructed vessels, 27 (20%) were angiographically free of epicardial disease, with no focal or diffuse lesions producing >10% stenosis as assessed with 3D quantitative coronary angiography. Most lesions (n = 87 (81%)) were in the proximal – mid vessel and median diameter stenosis across all cases was 31% [19 – 47] (see Table 1 for demographics and vessel characteristics and Text C in S1 Appendix for lesion data including screenshots of every reconstructed vessel and supplementary stl files S1 File, S2 File and S3 File for all coronary reconstructions).

**Table 1:** Patient demographics and lesion characteristics of included cases.

Demographics (n = 89)	
Age, y	63.5 ±10.6
Male	70 (79%)
BMI	30.3 ±8.8
Current or ex-smoker	47 (53%)
**Enrolment pathology**	
CCS	32 (36%)
STEMI	19 (21%)
NSTEMI	38 (43%)
**Comorbidities**	
Left ventricular ejection fraction	52.1 ±7.2
Hypertension	38 (43%)
Diabetes mellitus	18 (20%)
Hypercholesterolaemia	36 (40%)
Previous myocardial infarction	24 (27%)
Previous stroke	4 (4%)
**Vessel characteristics (n = 135)**	
Left anterior descending	82 (62%)
Left circumflex	13 (10%)
Right coronary artery	33 (25%)
Diagonal branch	2 (1%)
Obtuse marginal	2 (1%)
Intermediate	1 (1%)
Pre-PCI physiology assessment	64 (48%)
Vessel inlet diameter (mm)	3.0 ±0.5
Vessel outlet diameter (mm)	1.9 [1.7 – 2.2]
**Lesion characteristics**	
Stenosis present	108 (81%)
3D-QCA percentage stenosis	31 [19 – 47]
Stenosis location Proximal Mid Distal	44 (41%) 43 (40%) 21 (20%)
Tandem lesions	19 (18%)
Diffuse disease	31 (29%)
FFR	0.81 [0.70 – 0.89]
FFR significant disease (≤0.80)	62 (47%)

BMI, body mass index; CCS, chronic coronary syndrome; NSTEMI, non-ST elevation myocardial infarction; PCI, percutaneous coronary intervention; QCA, quantitative coronary angiography; STEMI, ST-elevation myocardial infarction.

**Fig 1:**
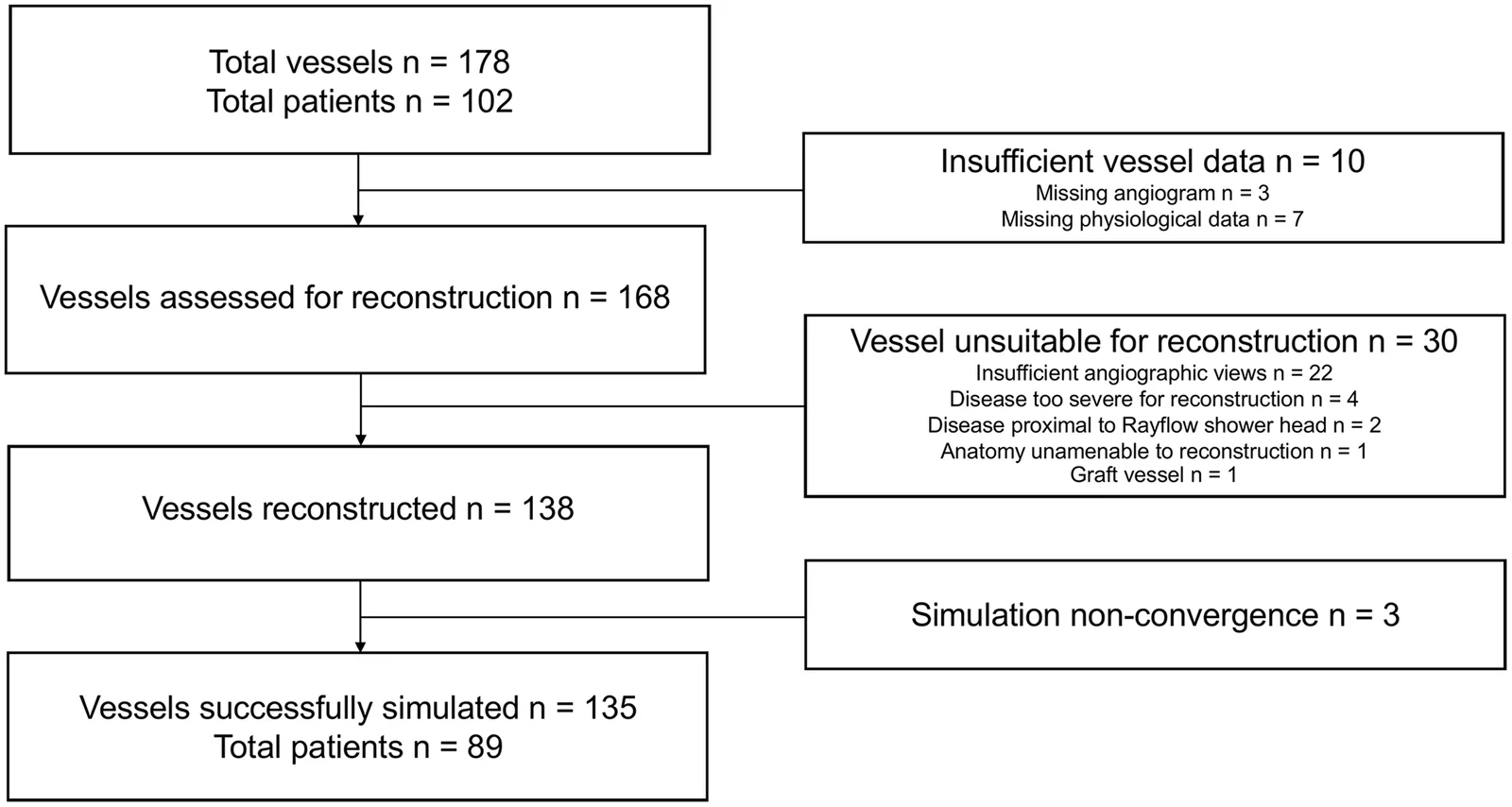
Study exclusion flowchart.

### MRR

MRR was computed successfully in 116 vessels. Median MRR_Virtu_ was 2.31 [1.83 – 3.00], which was significantly lower than median MRR_CIT_ (2.79 [2.21 – 3.52], z = 3.57, p = 0.0004). There was evidence of a moderate relationship between paired MRR measurements (ρ = 0.58, p < 0.0001). Across all cases, there was a tendency for virtuQ to underestimate MRR_CIT_. Agreement did however improve at lower values, closer to the diagnostic threshold (Fig 2). Overall diagnostic accuracy of MRR_Virtu_ was 70.6% (95% CI 61.5 – 78.6) and AUC was 0.77 (95% CI 0.68 – 0.86), indicating moderate diagnostic classification (Table 2, Fig 3). In keeping with the predefined diagnostic threshold of 2.5, ROC sensitivity analysis identified an optimal diagnostic threshold for MRR_Virtu_ of 2.5 (Figure D in S1 Appendix).

**Table 2:** Summary diagnostic accuracy results for total vessel resistance, microvascular resistance reserve (MRR) and coronary flow reserve (CFR). Median bias and 95% LOA computed with Bland-Altman quantile regression plots. 95% CI for sensitivity, specificity, positive and negative predictive values calculated with the Clopper–Pearson exact confidence interval and AUC 95% CI calculated with DeLong’s method. AUC, area under the curve; LOA, limits of agreement.

	MRR	CFR
Successful simulations	116 (85.9%)	116 (85.9%)
Median Bias at diagnostic threshold	-0.61	0.04
95% LOA at diagnostic threshold	-2.23 to 1.67	-2.54 to 1.88
Accuracy, % (95% CI)	69.8% (60.6 – 78.0)	71.6% (62.4 – 79.5)
Sensitivity, % (95% CI)	60.0% (38.7– 78.9)	89.4% (80.8 – 95.0)
Specificity, % (95% CI)	72.5% (62.2 – 81.4)	22.6% (9.6 – 41.1)
Positive predictive value, % (95% CI)	37.5% (22.7 – 54.2)	76.0% (66.4 – 84.0)
Negative predictive value, % (95% CI)	86.8% (77.1 – 93.5)	43.8% (19.8 – 70.1)
AUC (95% CI)	0.77 (0.68 – 0.86)	0.69 (0.58 – 0.80)

**Fig 2:**
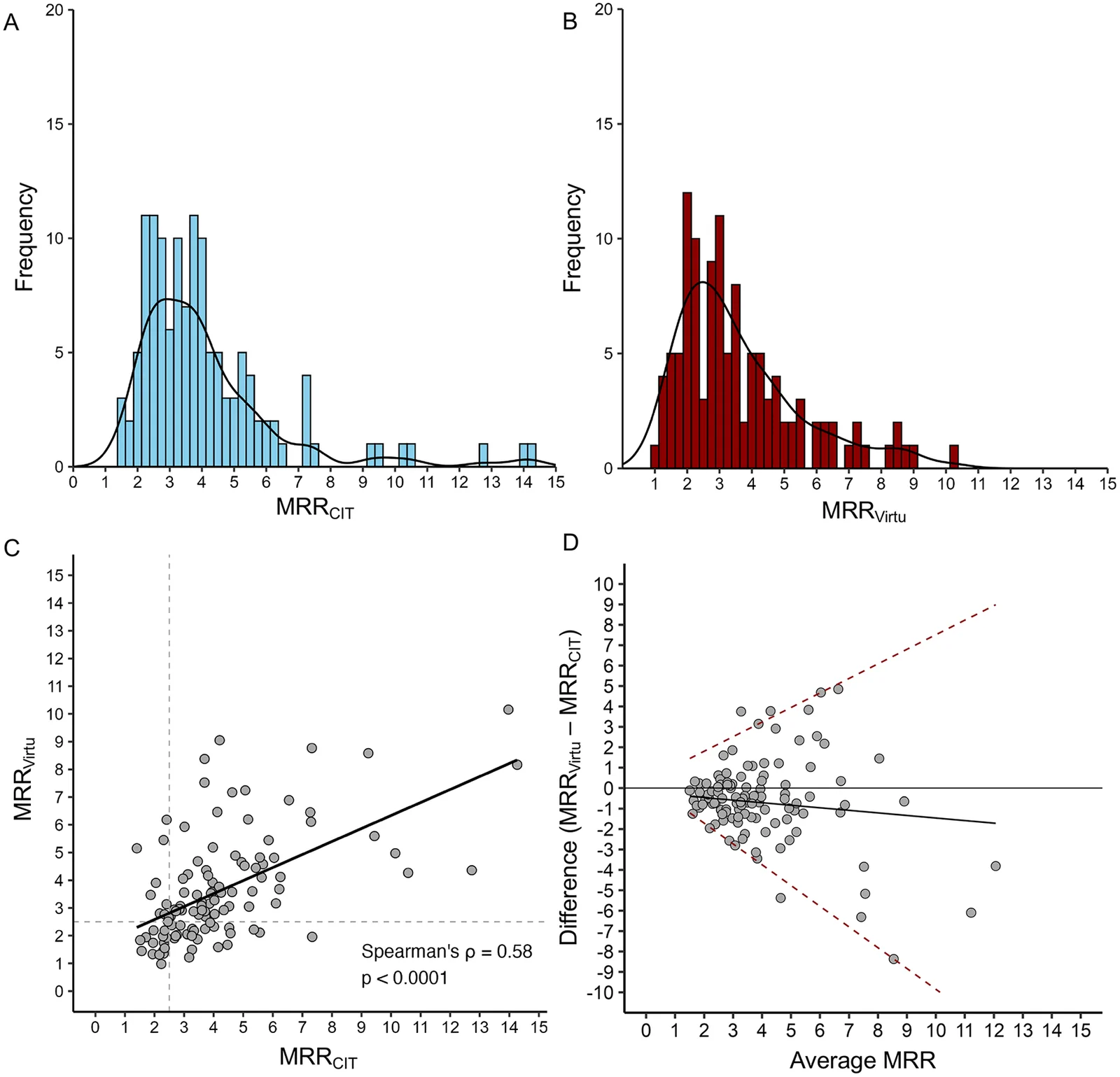
Summary and agreement results for MRR. A) Histogram of CIT-assessed MRR. B) Histogram of computed (virtuQ) MRR. C) Correlation plot and simple least regression for CIT versus virtuQ derived MRR (p value computed from Spearman’s rank correlation test). D) Bland-Altman plot demonstrating agreement between CIT and virtuQ derived MRR. Median bias (solid line) and 95% limits of agreement (dashed lines) derived from quantile regression. Data derived from 116 observations. CIT, continuous thermodilution; MRR, microvascular resistance reserve.

**Fig 3:**
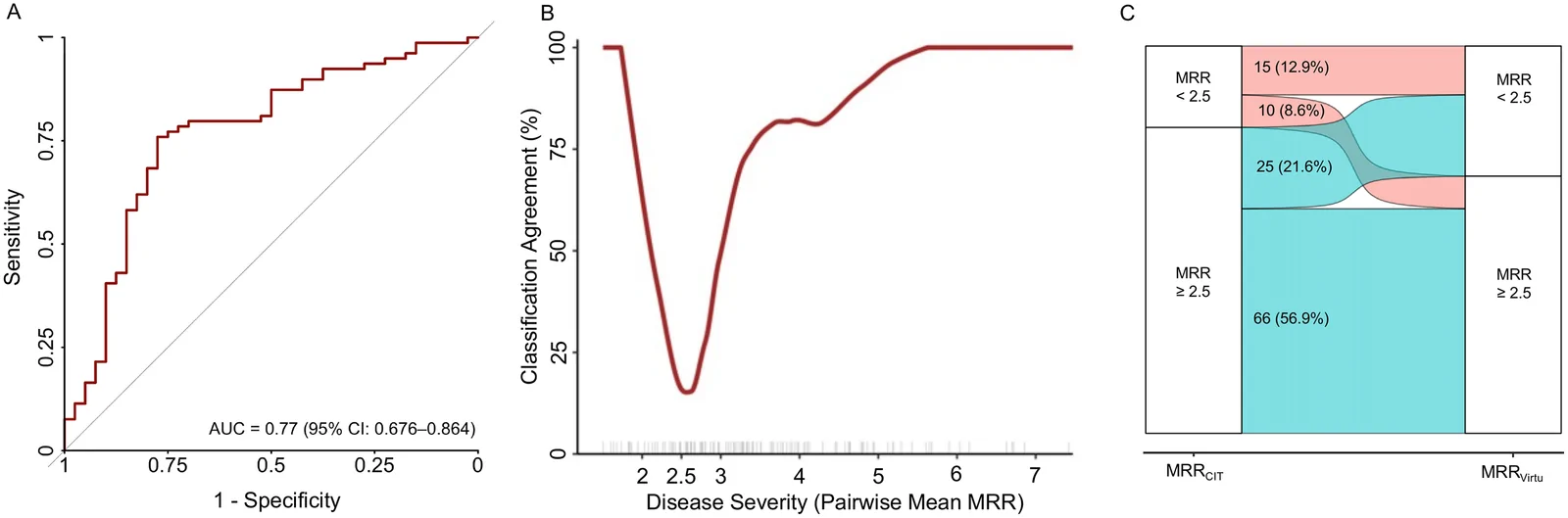
Diagnostic accuracy results for MRR. A) Receiver operator characteristic curve for MRR using a diagnostic threshold of 2.5 (95% CI calculated using DeLong’s method). B) LOESS-smoothed curve showing the percentage agreement between virtuQ and CIT MRR classifications across the range of disease severity, with individual data points indicated by rugs along the x-axis. C) Alluvial plot at diagnostic threshold 2.5. Data derived from 116 observations. AUC, area under the curve; CIT, continuous thermodilution; MRR, microvascular resistance reserve.

### Absolute flow

Hyperaemic flow was computed successfully in 131 vessels. At the vessel inlet, corresponding to the location of CIT assessment, median hyperaemic Q_Virtu_ was 194 [127 – 272] mL/min. This was significantly larger than hyperaemic Q_CIT_ (median 157 [112 – 205] mL/min, z = -4.5915, p < 0.0001). There was evidence of a moderate relationship between computed and CIT-assessed hyperaemic flow (ρ = 0.44, p < 0.0001). In arteries drawing lower flow (typically <200 mL/min), there was minimal overall bias between methods. However, at higher flow values, computed flow significantly overestimated hyperaemic Q_CIT_ with an associated worsening in limits of agreement (Fig 4).

**Fig 4:**
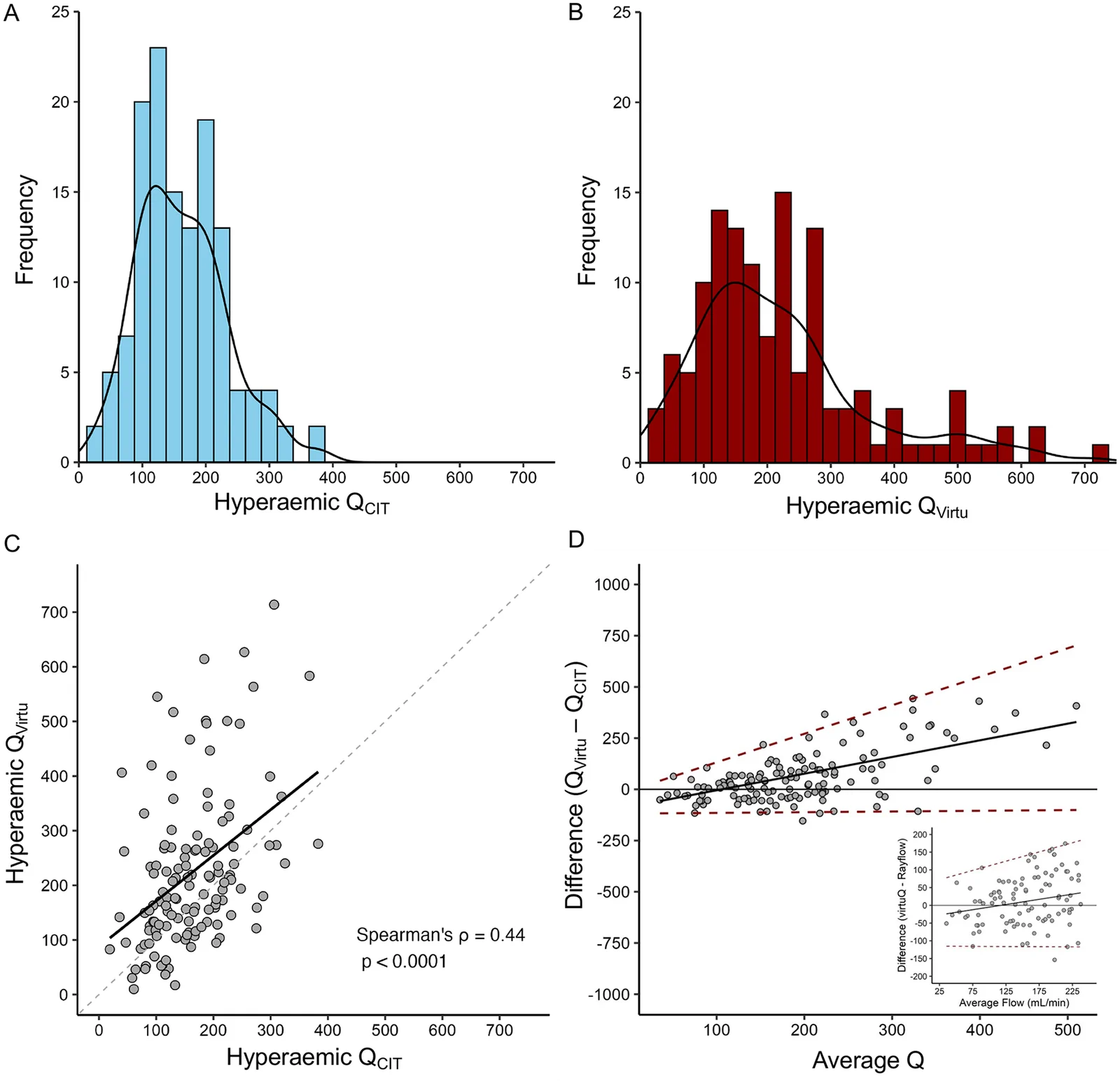
Summary results for hyperaemic flow. **A)** Histogram of CIT-assessed hyperaemic flow. **B)** Histogram of computed (virtuQ) hyperaemic flow. **C)** Correlation plot and simple least regression for CIT versus virtuQ derived hyperaemic flow (p value computed from Spearman’s rank correlation test). **D)** Bland-Altman plot demonstrating agreement between CIT and virtuQ derived hyperaemic flow. Median bias (solid line) and 95% limits of agreement (dashed lines) derived from quantile regression. The sub figure shows a magnified Bland-Altman plot at the lowest flow values. Data derived from 131 observations. CIT, continuous thermodilution.

### Vessel resistance and CFR

Hyperaemic Rμ was computed successfully in 131 vessels. Median Rμ_Virtu_ was 360 [238 – 524] WU, which was significantly lower than Rμ_CIT_ (median 452 [350 – 586] WU, z = -2.82, p = 0.0048). There was evidence of a weak relationship between computed and CIT-assessed Rμ (ρ = 0.39, p < 0.0001). For typical cases below 500 WU, there was a tendency for virtuQ to overestimate CIT-assessed resistance and vice-versa above this value (Figure K in S1 Appendix). CFR was computed in 116 vessels, disclosing a median value of 1.57 [1.33 – 1.94]. Computed CFR was significantly lower than median CFR_CIT_ (1.98 [1.60 – 2.55], z = -3.43, p = 0.0006), with evidence of a weak relationship between these values (ρ = 0.36, p < 0.0001). For cases with a CFR_CIT_ below the diagnostic threshold, there was a tendency for CFR_Virtu_ to overestimate this value and vice-versa above the diagnostic threshold. Across all cases, agreement was improved at lower CFR values (Figure L in S1 Appendix). Overall diagnostic accuracy of CFR_Virtu_ was 71.6% (95% CI 62.4 – 79.5) and AUC was 0.69 (95% CI 0.58 – 0.80)(see table 2 for full results). ROC sensitivity analysis identified an optimal diagnostic threshold for computed CFR of 2.7 (Figure M in S1 Appendix).

### Determinants of agreement

Correlation analysis identified uncertainty in the flow diameter scaling exponent as the most significant determinant of agreement for MRR (ρ = -0.36, p < 0.0001). The correlation matrix suggested no other variables, independent of side branch flow, were predictive of MRR agreement (Text B in S1 Appendix). SHAP coefficients could not be calculated for MRR agreement, due to inability to reliably fit a predictive model (Table B in S1 Appendix). For absolute flow, uncertainty in the flow diameter scaling (Huo-Kassab) exponent was identified as the dominant source of error (SHAP value = 72.6, ρ = -0.60, p < 0.0001), followed by FFR (SHAP value = 7.7, ρ = 0.24, p = 0.0051). A third variable, mismatch between FFR and CFD-derived hyperaemic stenosis resistance (HSR) was also associated with agreement (ρ = -0.40, p < 0.0001). Increasing HSR/FFR mismatch values are suggestive of either pressure losses proximal to the CIT infusion head or inaccurate reconstruction anatomy, but this mismatch variable was excluded from SHAP analysis to avoid data overfitting.

## Discussion

In this study, we evaluated the agreement between CFD- (virtuQ) and CIT-derived coronary physiology. The main results were:

1) MRR_Virtu_ demonstrated a moderate relationship between CIT-measured values with fair diagnostic classification and stronger agreement at lower, more clinically relevant, values.
2) Absolute hyperaemic Q_Virtu_ demonstrated a moderate relationship with CIT-measurements, with improved agreement at lower values.

### Current accuracy and future CFD developments

Previous validation studies of invasive techniques for quantifying microvascular disease (MVD) have shown mixed results (Table 3). The largest study that compared bolus thermodilution- against Doppler-derived index of microvascular resistance, in two expert centres, revealed only a modest correlation between the two methods measuring the same index (r = 0.43; P < 0.0001) [9]. More recent data from 175 patients demonstrated a lower correlation coefficient between bolus and CIT of 0.26 [24], and further work suggesting this may be as low as 0.11 [31,32]. In the current study, MRR_Virtu_ was the most directly applicable metric for quantifying microvascular-specific physiology (i.e., independent of epicardial lesion severity). Our reported correlation between MRR measurements (0.58, p < 0.0001) is comparable with recent studies of invasive techniques, but remains lower than the strong correlations originally observed between Doppler and bolus thermodilution (Table 3).

**Table 3:** Summary of key studies evaluating the agreement between invasive techniques for coronary microvascular physiology assessment. Cont denotes continuous infusion thermodilution. hMR expressed as the ratio between hyperaemic mean distal pressure and hyperaemic Doppler average peak velocity. IMR computed from hypaeremic mean distal pressure multiplied by hypaeremic bolus thermodilution mean transit time. *Denotes values computed from absolute bias reported in original study to facilitate comparison. p values for correlation reproduced as reported in original study. ACS, acute coronary syndrome; CCS, chronic coronary syndrome; CFR, coronary flow reserve; CIT, continuous thermodilution; hMR, hyperaemic microvascular resistance; IMR, index of microvascular resistance; MRR, microvascular resistance reserve; PET, positron emission tomography; Q, Absolute flow (mL/min).

Study	Patients (n)	Number of vessels	Assessment methods	Mean values ±SD	Correlation	Percentage bias
Pijls 2002 [26]	CCS (50)	92	Doppler and bolus thermodilution	CFR_Dopp_ = 1.47 ± 0.52 CFR_bolus_ = 1.49 ± 0.51	r = 0.80; p<0.001	17 ± 14%
Barbato 2004 [27]	CCS (34)	42	Doppler and bolus thermodilution	CFR_Dopp_ = 2.0 ± 0.8 CFR_bolus_ = 2.2 ± 0.9	r = 0.79; p<0.001	–
Everaars 2018 [28]	CCS (40)	92	Doppler and bolus thermodilution	CFR_Dopp_ = 3.2 ± 1.4 CFR_bolus_ = 2.6 ± 0.8	r = 0.59; p < 0.001	16 ± 36%
Everaars 2019 [11]	CCS (25)	44	CIT and [^15^O]H_2_O PET	Q_cont_ = 174 ± 72 Q_PET_ = -	r = 0.91; p < 0.001	0 ± 20%*
De Bruyne 2021 [29]	CCS (26) NSTE-ACS (1)	30	Doppler and CIT	MRR_Dopp_ = 3.29 ± 1.18 MRR_cont_ = 3.58 ± 1.25	r= 0.86; p<0.001	8 ± 19%*
Demir 2022 [9]	CCS (135) ACS (115)	250	Doppler and bolus thermodilution	CFR_Dopp_ = 2.05 ± 0.89 CFR_bolus_ = 2.59 ± 1.46	r = 0.60; p<0.001	25 ± 53%*
				hMR = 2.51 [2.00-3.40] IMR = 22.04 [14.25-35.18]	r = 0.37; p<0.001	–
Gallinoro 2023 [24]	CCS (175)	175	Bolus and CIT	MRR_bolus_ = 4.40 ± 1.99 MRR_cont_ = 3.22 ± 1.02	r = 0.26; p<0.001	31 ± 52%*
				CFR_bolus_ = 3.47 ± 1.42 CFR_cont_ = 2.67 ± 0.81	r = 0.28; p<0.001	26 ± 52%*
Jansen 2023 [30]	CCS (248)	–	Bolus and CIT	MRR_bolus_ = 4.2 ± 2.5 MRR_cont_ = 3.1 ± 1.1	ρ=0.28; p<0.001	33 ± 73%*
				CFR_bolus_ = 3.5 ± 1.8 CFR_cont_ = 2.6 ± 1.0	ρ=0.31; p<0.001	30 ± 51%*

Agreement between virtuQ and CIT was strongest at the lower, more clinically relevant, values. At the diagnostic threshold overall bias was 24 ±40% and while this is comparable with recent validation studies of invasive techniques, it represents a systematic underestimation of MRR_CIT_ by MRR_Virtu_ of around 0.6. In practice, this bias increases the proportion of patients with abnormal results when virtuQ is used and makes the CFD method a more appropriate tool for ruling MVD out versus in (PPV 38% versus NPV 87%). While diagnostic accuracy is inevitably weakest at the diagnostic threshold, this error increases the number of grey zone cases where diagnostic confidence is low. Thus, the current study suggests that, with CIT as the reference, the accuracy of the novel CFD method may be comparable to routinely used techniques like Doppler and bolus thermodilution in terms of assessing microvascular physiology. Nevertheless, the virtuQ technique may be implemented in a standard catheterisation laboratory, with any pressure-sensitive guidewire, to produce results within five minutes. Furthermore, in the current study, the relationship between CIT-assessed and virtuQ-derived MRR was comparable [33–35] with that reported for other angiography-derived (computed) techniques for microvascular assessment.

The relationship between virtuQ and CIT-assessed absolute flow at the vessel inlet was moderate. Error propagation analysis suggested that error in the computational method was attributable primarily to the method of side branch flow incorporated into the CFD simulation. This is consistent with similar previous analyses [36]. For the current study, the side branch method used a population-averaged scaling exponent of 7/3, supported by theoretical [19] and meta-analysis studies [20], but further work may wish to investigate novel models of side branch flow incorporation to reduce error [37]. Additional sources of error included mismatch between FFR and CFD-derived HSR. This may be attributable to either the artificial stenosis created by the CIT microcatheter in the left main stem, organic causes of pressure loss proximal to the microcatheter infusion head (i.e., left main stem disease) or inaccuracies in 3D reconstructed anatomy.

Agreement was also modestly dependent on FFR, which may be multifactorial in aetiology; a low pressure gradient principally sensitises the simulation to errors in reconstruction diameter. Additionally, with minimal epicardial stenoses, dynamic pressure losses within the vessel become non-negligible and these are not currently accounted for in CFD simulations. The lack of inclusion of dynamic pressure may be particularly important for CFR and MRR calculations; omitting this from simulations is more likely to introduce error when total pressure gradients (Pa minus Pd) are low. This disproportionately affects baseline flow calculations and consequently has a heightened influence over CFR and MRR calculations versus Q and Rμ which are derived solely from hyperaemic pressure measurements. These factors may provide targets to improve simulations and adjust clinical data collection protocol for further prospective validation against CIT.

### Clinical implications

The importance of MVD for current diagnosis and management of CCS is increasingly recognised [1,2,38,39]. In the context of CCS, current guidelines advocate for assessment of MVD in the absence of significant (obstructive) epicardial disease [1]. Indeed, a seminal controlled trial of MVD treatment (CorMicA) randomised only patients without obstructed coronary arteries [40]. Given the increased cost and time associated with invasive microvascular assessment, this appears a sensible approach, because it strikes a balance between test sensitivity and the pressures of real-world clinical practice. The current results, where agreement between CIT and virtuQ measurements weakens in vessels lacking an appreciable epicardial stenosis, suggest the CFD method may not be suitable for microvascular assessment in a population enriched by patients with suspected angina with non-obstructive coronary arteries (ANOCA). However, the assumption of a predominant phenotype where pathology lies exclusively in the microvascular compartment appears biologically unlikely; large registry data has shown MRR to be a stronger independent predictor of major adverse cardiac events (MACE) in patients with versus without obstructive epicardial stenoses [7]. Further, recent re-analysis of the ORBITA trial [41], using virtuQ, suggested MVD may mediate the symptomatic response to PCI in the context of flow-limiting epicardial stenoses despite optimal medical therapy [42].

In the context of acute coronary syndromes, the indication for invasive physiological assessment is less clear (IIb, B recommendation) [43]. As MVD is independently predictive of MACE in this cohort [44], assessment may help stratify those in need of aggressive pharmacotherapy. However, during and shortly after an acute insult, the coronary microvasculature undergoes dynamic changes which influence physiology results. This complicates dichotomisation of patients, during a single invasive catheterisation, as having a favourable versus unfavourable physiological profile. Under circumstances where acute MVD restricts flow, FFR is likely to increase, and this would reduce accuracy of virtuQ.

Should focused microvascular assessment be considered for a wider patient cohort, our results suggest virtuQ may effectively rule MVD in or out in most patients and identify those most likely to benefit from full invasive assessment. Taking the results of this study, using MRR thresholds of 1.8 and 2.5 for ruling MVD in or out respectively would include 89 (76.7%) vessels and yield sensitivity and specificity of 87.3% and 85.0% respectively. While in-procedure (online) virtuQ assessment may be performed faster than CIT measurements can be taken [45], the CFD method still requires a pressure wire. This requirement for coronary instrumentation poses a barrier to routine assessment in all vessels, especially for diagnostic only catheterisation laboratories. Alternative wire-free computational methods have been described, where flow is inferred from thrombolysis in myocardial infarction frame counting. While comparable diagnostic accuracy results have been reported in a similar cohort to the current study [34], vessel foreshortening and geometric reconstruction errors contribute uncertainty to estimated flow.

As total vessel resistance and CFR are influenced by disease in both the microvascular and epicardial compartments, their specificity for MVD detection in stenotic vessels is limited. MRR may therefore provide the most universally applicable microvascular assessment. It is also reassuring that the sensitivity analyses identified a diagnostic threshold for MRR (2.5) which is consistent with the results of other invasive studies and the guidelines [25,29].

## Limitations

None of the current invasive techniques for measuring flow or microvascular resistance represent a ‘ground-truth’, and this should be borne in mind when interpreting the results. Absolute resistance measurements in the distal third of the vessel are unreliable with continuous infusion, due to the artificial stenosis created by the microcatheter, meaning direct validation of virtuQ absolute resistance at the vessel outlet was not possible. The virtuQ technique requires a distal pressure measurement, necessitating coronary instrumentation, and two high-quality angiographic projections may theoretically increase contrast use. Compared with validation studies of invasive microvascular assessment techniques, our cohort contained a greater proportion of cases with low MRR (likely attributable to the greater proportion of ACS patients) thus limiting the strength of external study comparisons. As pilot validation data suggested agreement between virtuQ and CIT did not meet the assumptions required for simple Bland-Altman analysis [15], a power calculation for sample size could not be calculated [46]. Agreement data for numerically higher flow and resistance values are derived from a relatively small number of data points and lack statistical power. Furthermore, accuracy may be poorer in patients undergoing assessment for ANOCA. MRR measurement in focally stenosed epicardial arteries did not account for collateral flow with wedge pressure measurements. MRR agreement did not however improve in the subgroup of cases with high FFR (>0.85, Figure G in S1 Appendix). The current study evaluated agreement only in the context of structural microvascular disease, although the same principles may be applied with pressures acquired during acute vasoreactivity testing. There is no diagnostic threshold for absolute flow (mL/min) reduction, which is the subject of ongoing research [47]. As an exploratory secondary analysis, results from the SHAP and sub-group analyses are hypothesis generating and should be interpreted with caution.

## Conclusion

Our CFD workflow allows simultaneous assessment of absolute coronary blood flow and indices of coronary physiology, from standard pressure wire data, with a modest agreement against CIT measurements. MRR demonstrated the strongest association with invasive measurements, giving fair diagnostic classification and, with future work, may represent an effective strategy for gatekeeping invasive microvascular assessment.

## Supporting information

S1 Appendix Text A: SHAP analysis included variables. Text B: SHAP analysis technical specifications. Figure A: Distribution of invasive FFR values in included cases. Figure B: Distribution of epicardial lesion severity in included cases. Figure C: Distribution of hyperaemic outlet flow and associated distal microvascular resistance. Text C: All 131 included cases with computed hyperaemic flowTable A: Demographics and lesion characteristics of excluded patients. Figure D: ROC sensitivity analysis for microvascular resistance reserve (MRR). Figure E: Quantile regression Bland-Altman plot for microvascular resistance reserve (MRR) displaying quantile regression line equations. Table B: MRR SHAP analysis results. Figure F: Agreement results for MRR stratified by ACS vs CCS cohorts. Figure G: Agreement results for MRR in FFR >0.85 cohort. Text D: Rayflow/virtuQ hyperaemic flow Bland-Altman quantile regression line equations. Table C: Absolute inlet flow SHAP analysis results. Figure H: Agreement results for hyperaemic flow stratified by ACS vs CCS cohorts. Figure I: Before and after dot plot for hyperaemic inlet flow. Figure J: Summary results for total resting flow. Figure K: Summary results for total vessel resistance. Figure L: Summary results for coronary flow reserve (CFR). Figure M: ROC sensitivity analysis for coronary flow reserve (CFR)
(PDF)

S1 File All Essex STL reconstruction files. (ZIP)

S2 File Oxford STL reconstruction files 1 of 2. (ZIP)

S3 File Oxford STL reconstruction files 2 of 2. (ZIP)

S1 Fig Graphical Abstract: Graphical Abstract. (TIFF)
