# Supplementary material for: Agreement of an invasive angiography-derived computational model of coronary microvascular physiology against continuous thermodilution
Source: PLOS Digit Health. 2026 Nov 13;5(11):e0001830. doi: 10.1371/journal.pdig.0001830 (PMC13766283; doi:10.1371/journal.pdig.0001830)

Supplementary Material for:

Agreement of an invasive angiography-derived computational model of coronary microvascular physiology against continuous thermodilution

Contents

Page 2 – 3:	SHAP analysis included variables
Page 4:	SHAP analysis technical specifications
Page 5:	Distribution of invasive FFR values in included cases
Page 6:	Distribution of epicardial lesion severity in included cases
Page 7:	Distribution of computed physiology values
Page 8 – 22:	Details of every included case
Page 23:	Demographics and lesion characteristics of excluded patients
Page 24:	ROC sensitivity analysis for MRR
Page 25:	MRR Bland-Altman quantile regression line equations
Page 26:	MRR SHAP analysis results
Page 27:	Agreement results for MRR stratified by ACS vs CCS cohort
Page 28:	Agreement results for MRR in FFR >0.85 group
Page 29:	Flow Bland-Altman quantile regression line equations
Page 30 – 32:	Absolute inlet flow SHAP analysis results
Page 33:	Agreement results for flow stratified by ACS vs CCS cohort
Page 34:	Before and after dot plot for hyperaemic inlet flow
Page 35:	Summary results for resting flow
Page 36:	Summary agreement results for total vessel resistance
Page 37:	Summary agreement results for CFR
Page 38:	ROC sensitivity analysis for CFR

Text A: SHAP analysis included variables

For SHAP analysis, we considered 13 variables. Given a total of 131 vessels with hyperaemic flow results, this preserved a ratio of variables to data points of 1:10 as to try and avoid overfitting data. The variables considered are listed below along with a brief justification:

1. Fractional flow reserve (FFR): For high FFR cases, the total pressure delta driving flow from the vessel inlet to outlet will be low. Given FFR is frequently expressed as the ratio of integer values, the precision of the pressure wire will contribute significantly greater uncertainty for health cases. Additionally, the CFD methodology does not consider dynamic pressure changes which are likely to exert a greater influence at lower pressure deltas. Finally, at very low FFR values, there is likely to be a considerable stenosis restricting adequate opacification of the vessel lumen on angiography.

2. Flow diameter scaling (Huo-Kassab) exponent: This is likely the primary determining factor when computing side branch flow (opposed to using an alternative model of leak). Given significant intra-population uncertainty has been described, we compared the differences in flow when using the Huo-Kassab exponent of 2.33 and the Murray exponent of 3.0.

3. Proximal taper: Defined as the ratio between the smallest diameter in the proximal 10% of the reconstructed vessel against the inlet diameter. A significant degree of taper in the proximal vessel may lead to unstable simulation results.

4. Proximal estasia (i.e., pathological downstream vessel widening): Defined as the ratio between the largest diameter in the proximal 10% of the reconstructed vessel against the inlet diameter. A significant degree of ectasia in the proximal vessel may lead to unstable simulation results.

5. Distal taper: Defined as the ratio between the smallest diameter in the distal 10% of the reconstructed vessel against the inlet diameter. A significant degree of taper in the distal vessel may lead to unstable simulation results.

6. Distal vessel ectasia: Defined as the ratio between the largest diameter in the distal 10% of the reconstructed vessel against the inlet diameter. A significant degree of ectasia in the distal vessel may lead to unstable simulation results.

7. Vessel length: The Rayflow technique recommends 70mm distance between the shower head and location of distal pressure measurement. A significant difference in this result, particularly lower values, are likely to influence factors such as complete mixing of blood with saline infusate and heat transfer to the vessel wall which may worsen accuracy.

8. Inlet vessel diameter: A particularly large or small inlet may be indicative of error in the vessel reconstruction. We have previously used OCT data to show the virtuQ reconstruction protocol underestimates true lumen radius in the proximal vessel.

9. Total vessel taper (inlet – outlet diameter): A high taper not only influences side branch flow but also increases pressure losses within the vessel. The balance

between increasing uncertainty from side branch losses versus improved precision and simulation fidelity from pressure delta is unknown.

10. 3D quantitative coronary angiography diameter stenosis: The virtuQ technique is designed primarily for use in cases with an intermediate stenosis. In cases with a low % stenosis, there the simulation becomes increasingly sensitive to anatomical accuracy in the reconstruction inlet and outlet. Further, for very stenosed vessels, opacification of the vessel lumen on angiography may be compromised, thus limiting reconstruction accuracy.

11. Focal versus diffuse disease: The virtuQ reconstruction protocol was designed specifically for focal lesions. In cases of diffuse disease, anatomical accuracy may be worse.

12. Lesion location: Lesions in the proximal and distal vessel may not allow for sufficient development of flow within the simulation.

13. Hyperaemic stenosis (HSR)/ FFR mismatch: Computed using LOG(1 + HSR)/(1 - FFR). This provides a metric from 0 to infinity which gives an estimate of the concordance between these two measures of epicardial lesion physiology. FFR is invasively measured which HSR is computed from the reconstructed artery in virtuQ. Consequently, should a significant source of pressure loss within the vessel not be captured within the CFD simulation, mismatch will increase. Possible sources of error for a higher mismatch include significant pressure wire drift, the effect of the artificial stenosis induced by the Rayflow catheter, significant left main stem disease, inaccurate anatomical reconstruction.

Text B: SHAP analysis technical specifications

Before deriving SHAP coefficients, we examined for possible correlations between included variables. For absolute flow, we identified strong associations between

- FFR with 3D QCA
- Proximal taper with proximal ectasia
- Distal taper with distal ectasia
- Reconstruction inlet diameter with total vessel taper
- Flow-diameter scaling exponent with HSR/FFR mismatch

The correlation matrix is shown below

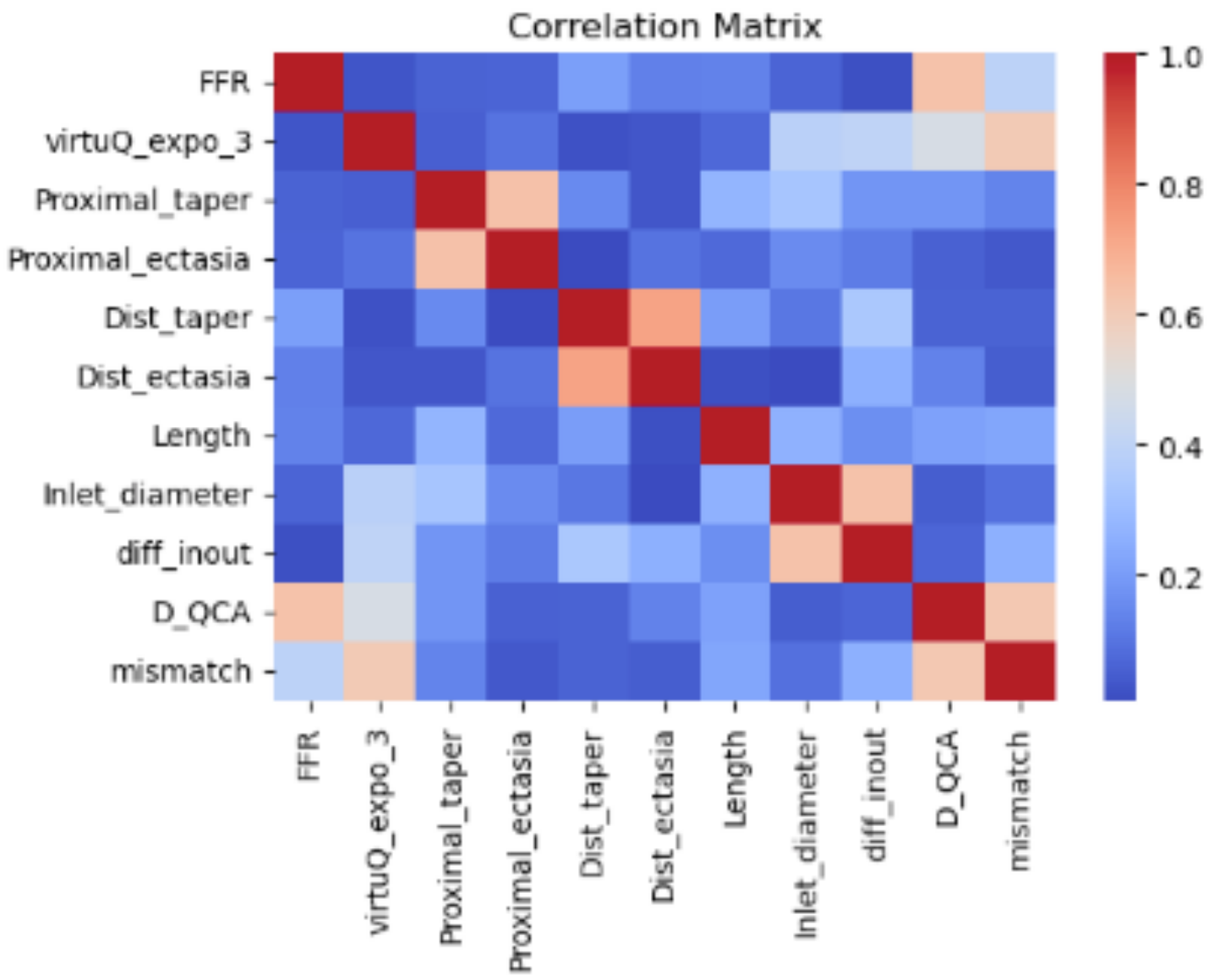

Accordingly, to reduce the likelihood of data overfitting and improve interpretability of SHAP coefficients, we removed the following variables from the analysis: 3D QCA, proximal ectasia, distal ectasia, total vessel taper and HSR/FFR mismatch.

We then split the data into training (80% of data) and test sets (20%). Random Forest regression and Ridge Linear Regression models were then trained, with the difference between computed and measured inlet flow as a target. We performed 5-fold cross validation on the training set and used the optimum model for evaluation on the test set. Because of the modest sample size and high result variability, we repeated the analysis 60 times with different train-test splits and averaged the cross-validation train R^2 scores, the cross-validation test R^2 scores, test R^2 scores and SHAP values for cross-validation test folds and test set.

Figure A: Distribution of invasive FFR values in included cases
131 observations

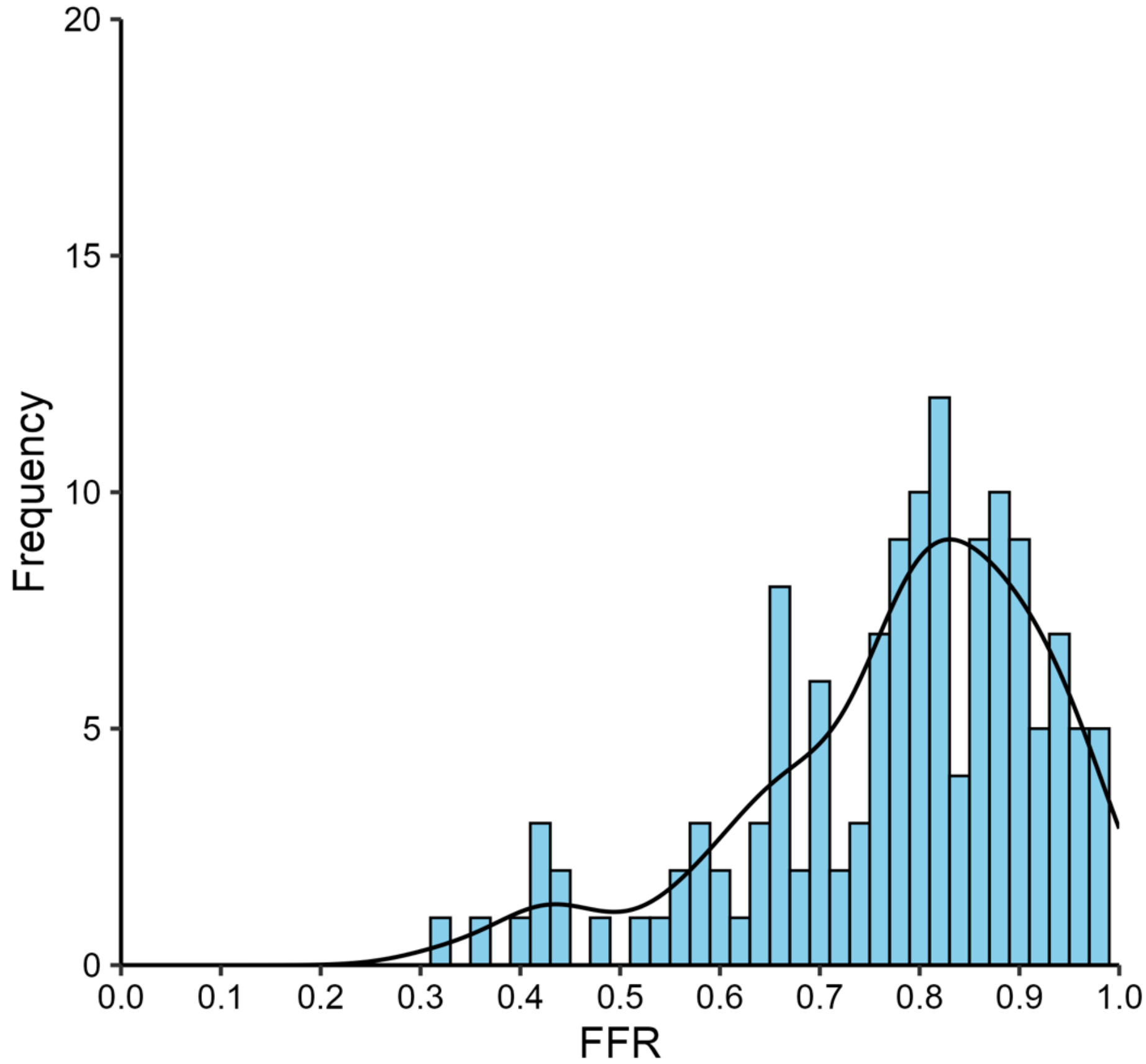

Figure B: Distribution of epicardial lesion severity in included cases
131 observations

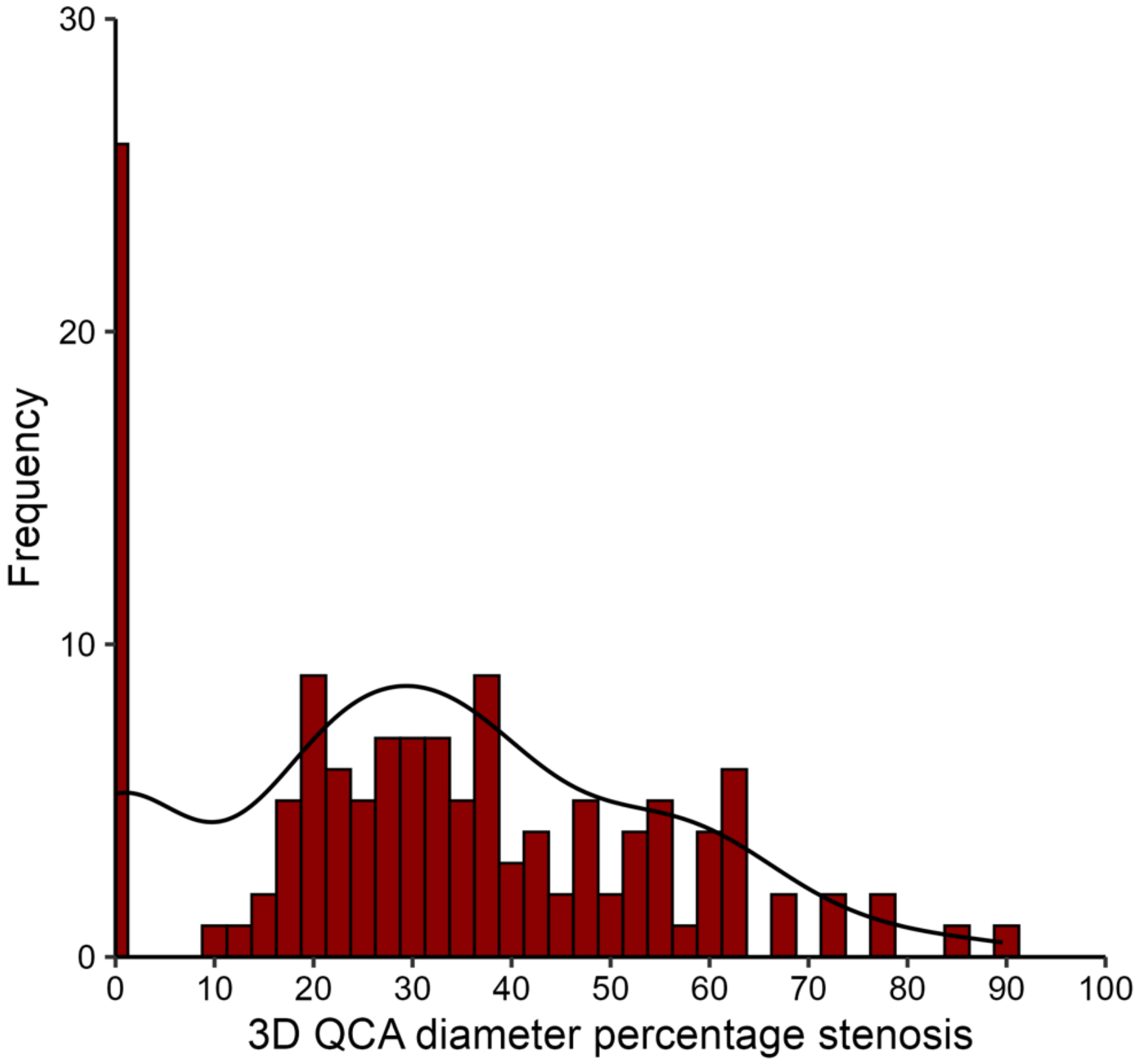

Figure C: Distribution of hyperaemic outlet flow and associated distal microvascular resistance

Microvascular resistance (MVR) calculated from distal pressure/outlet flow)

131 observations

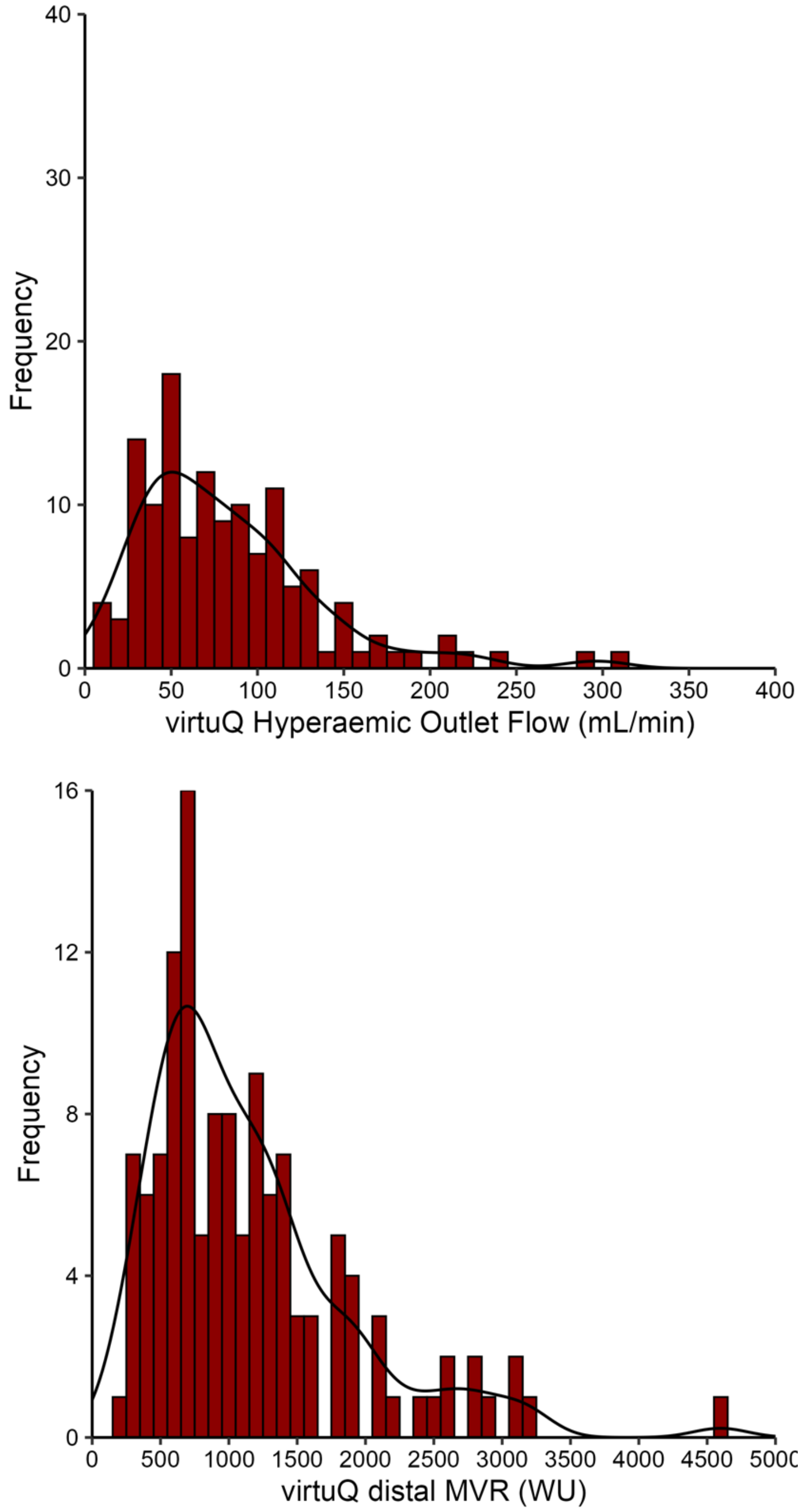

Text C: All 131 included cases with computed hyperaemic flow

For every case we include a screenshot of the 3D reconstruction with pressure distribution mapped along the vessel surface, a scale for colours and corresponding pressure drop is provided below the final case. We also include angiogram screenshots with segmented vessel centreline backprojected onto the angiogram and key invasive and simulated physiology (inlet diameter, outlet diameter, 3D quantitative coronary angiography (QCA) percentage diameter stenosis, invasive FFR and simulated flow at the vessel inlet and outlet under hyperaemia).

Case E1

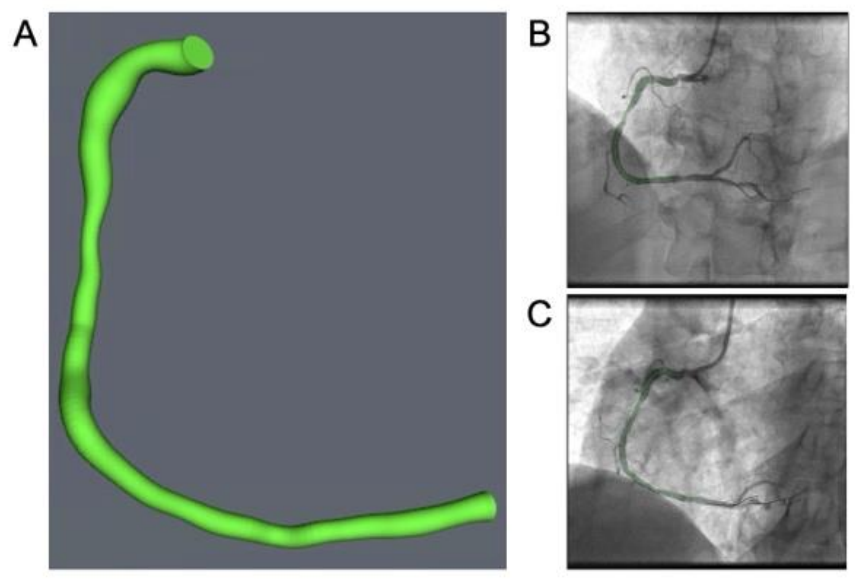

Inlet diameter: 3.26 mm
Outlet diameter: 2.71 m
3D QCA: 33%
FFR: 82/85 mmHg
Hyp inlet Q: 52 mL/min
Hyp outlet Q: 32 mL/min

Case E2

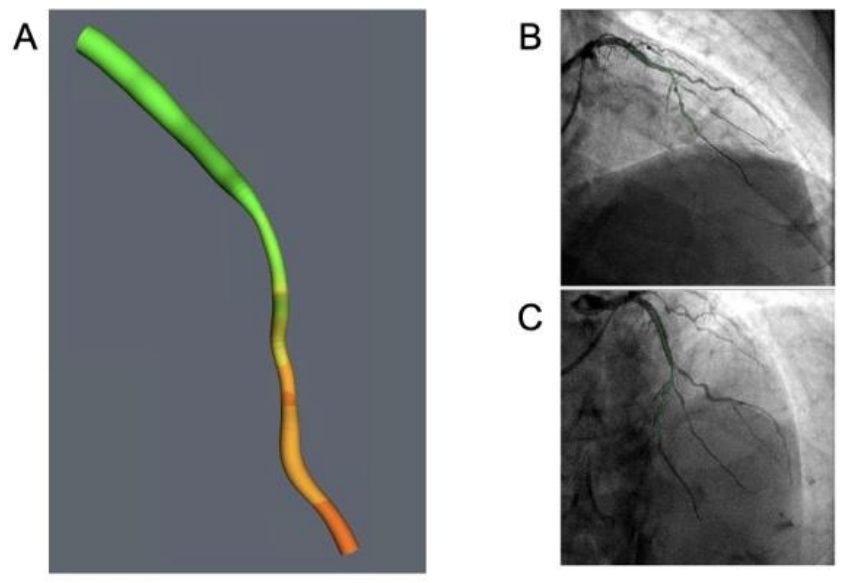

Inlet diameter: 2.75 mm
Outlet diameter: 1.60 m
3D QCA: 34%
FFR: 55/74 mmHg
Hyp inlet Q: 125 mL/min
Hyp outlet Q: 35 mL/min

Case E3

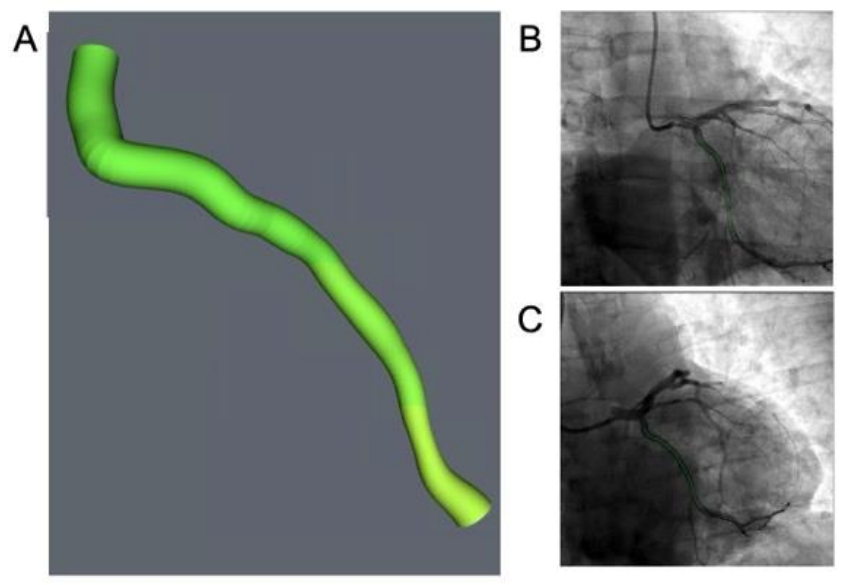

Inlet diameter: 3.00 mm
Outlet diameter: 2.77 m
3D QCA: 35 %
FFR: 79/92 mmHg
Hyp inlet Q: 160 mL/min
Hyp outlet Q: 129 mL/mi

Case E4

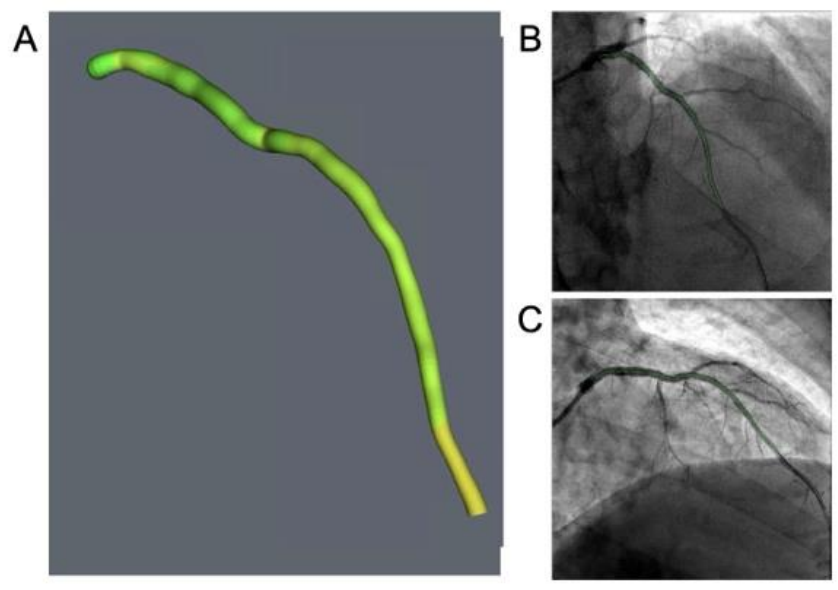

Inlet diameter: 2.86 mm
Outlet diameter: 2.07 m
3D QCA: 0 %
FFR: 87/106 mmHg
Hyp inlet Q: 273 mL/min
Hyp outlet Q: 121 mL/mi

Case E5

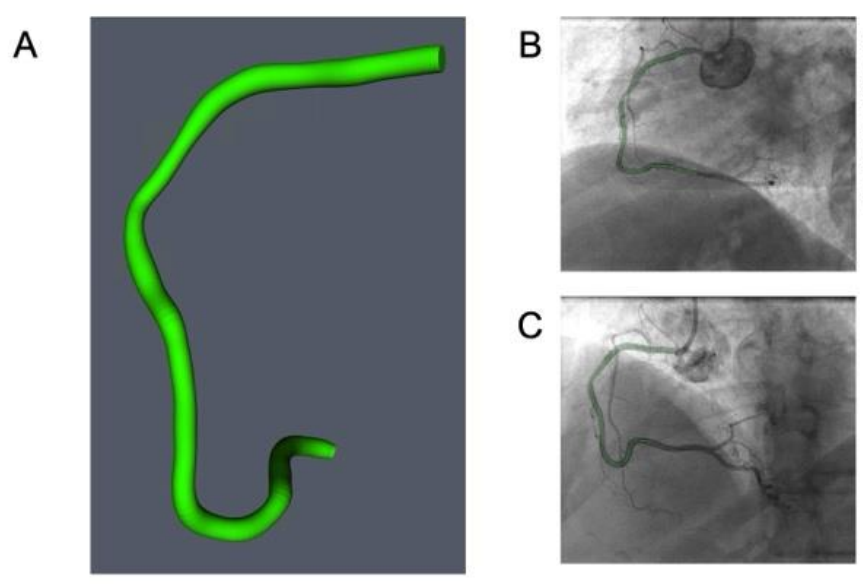

Inlet diameter: 2.34 mm
Outlet diameter: 1.58 m
3D QCA: 29 %
FFR: 97/101 mmHg
Hyp inlet Q: 48 mL/min
Hyp outlet Q: 21 mL/min

Case E6

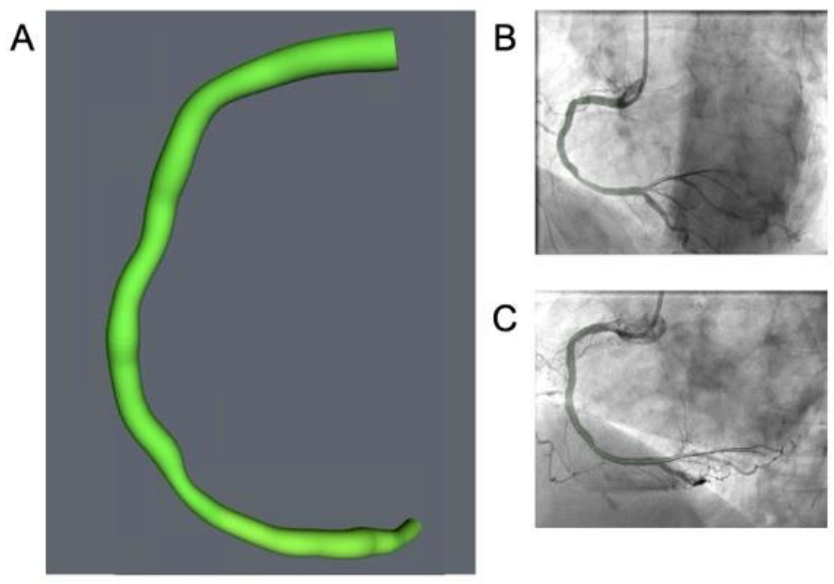

Inlet diameter: 3.65 mm
Outlet diameter: 1.91 mm
3D QCA: 34 %
FFR: 88/93 mmHg
Hyp inlet Q: 192 mL/min
Hyp outlet Q: 47 mL/min

Case E9

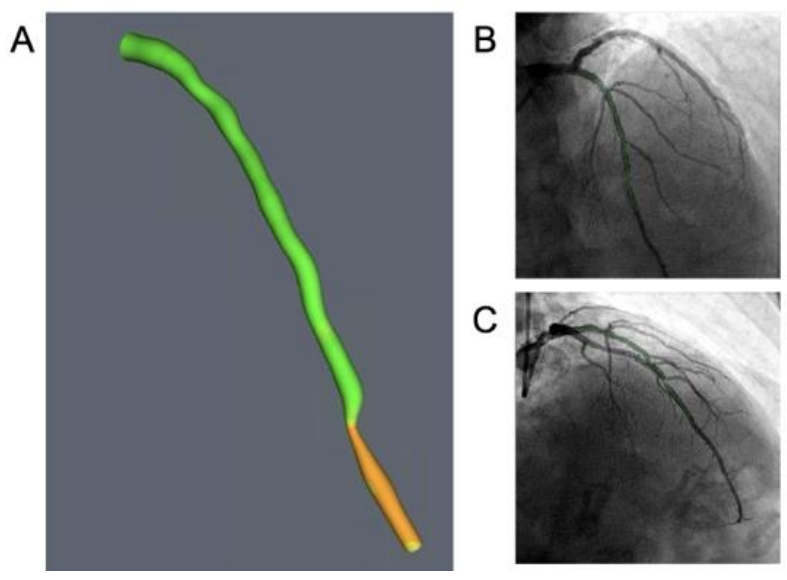

Inlet diameter: 3.00 mm
Outlet diameter: 1.80 mm
3D QCA: 55 %
FFR: 68/89 mmHg
Hyp inlet Q: 159 mL/min
Hyp outlet Q: 51 mL/min

Case E11

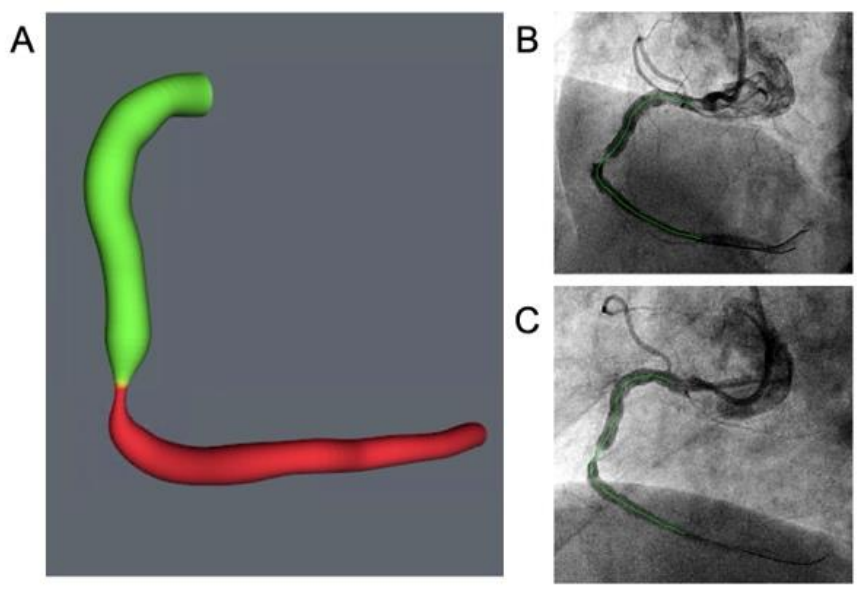

Inlet diameter: 3.07 mm
Outlet diameter: 1.86 mm
3D QCA: 73 %
FFR: 37/87 mmHg
Hyp inlet Q: 142 mL/min
Hyp outlet Q: 38 mL/min

Case E12

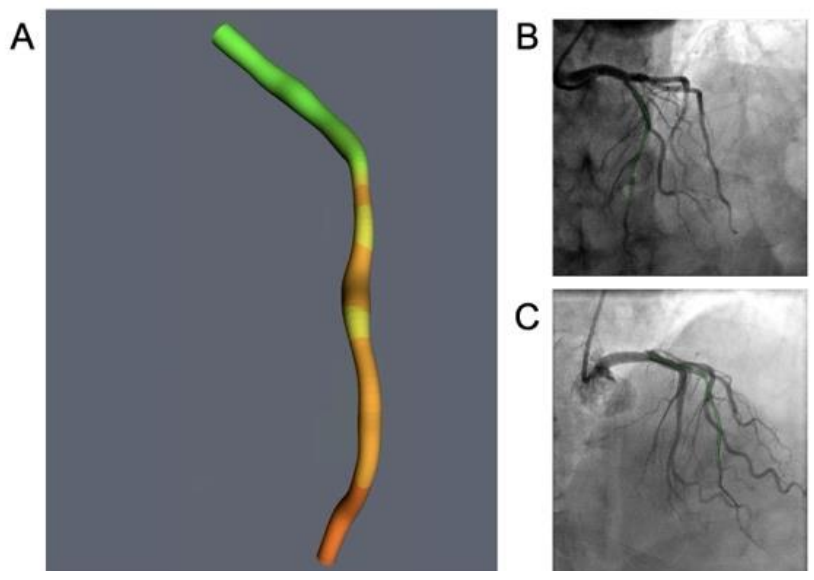

Inlet diameter: 2.33 mm
Outlet diameter: 1.53 mm
3D QCA: 35 %
FFR: 70/90 mmHg
Hyp inlet Q: 183 mL/min
Hyp outlet Q: 69 mL/min

Case E14

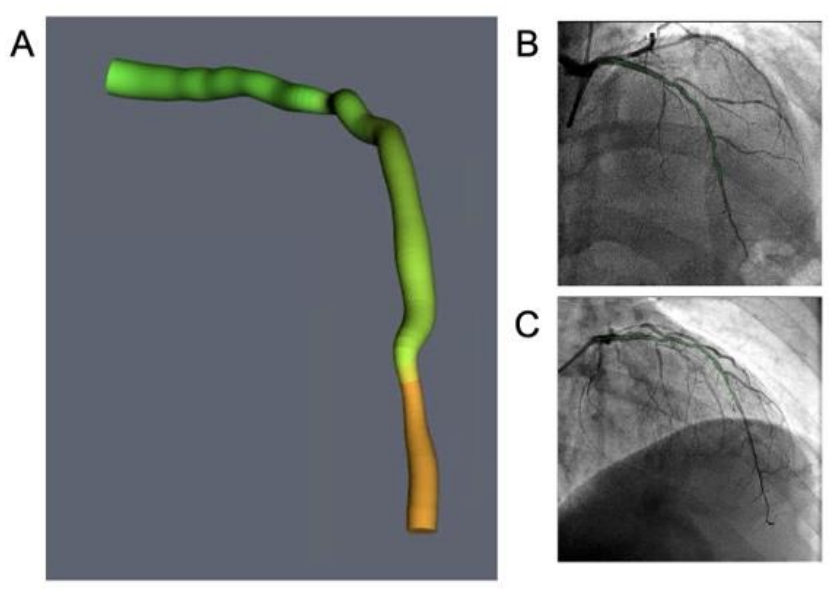

Inlet diameter: 3.21 mm
Outlet diameter: 1.66 mm
3D QCA: 37 %
FFR: 85/108 mmHg
Hyp inlet Q: 261 mL/min
Hyp outlet Q: 59 mL/min

Case E15

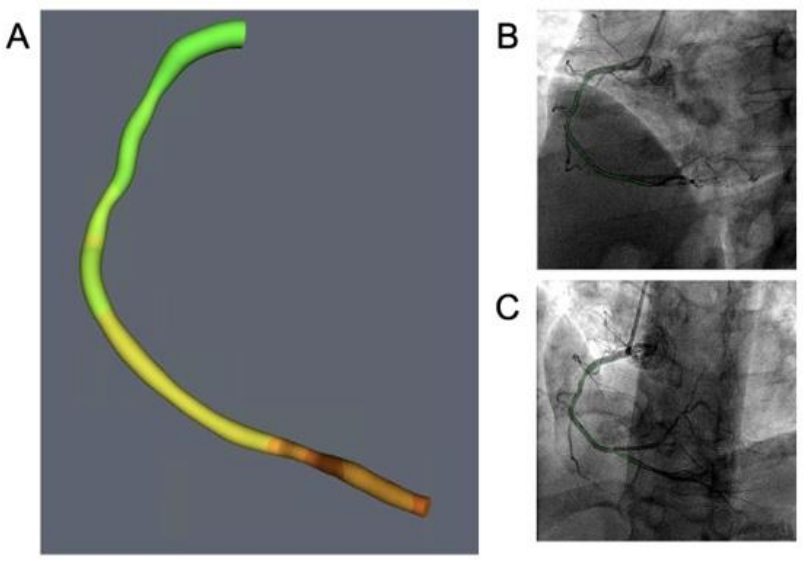

Inlet diameter: 2.64 mm
Outlet diameter: 1.74 mm
3D QCA: 33 %
FFR: 76/102 mmHg
Hyp inlet Q: 173 mL/min
Hyp outlet Q: 65 mL/min

Case E16

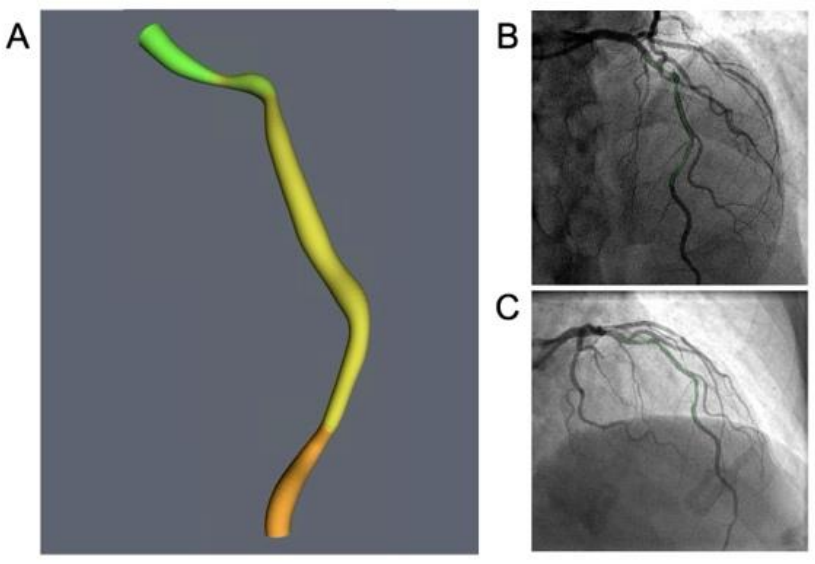

Inlet diameter: 2.82 mm
Outlet diameter: 2.10 mm
3D QCA: 52 %
FFR: 68/88 mmHg
Hyp inlet Q: 101 mL/min
Hyp outlet Q: 48 mL/min

Case E17

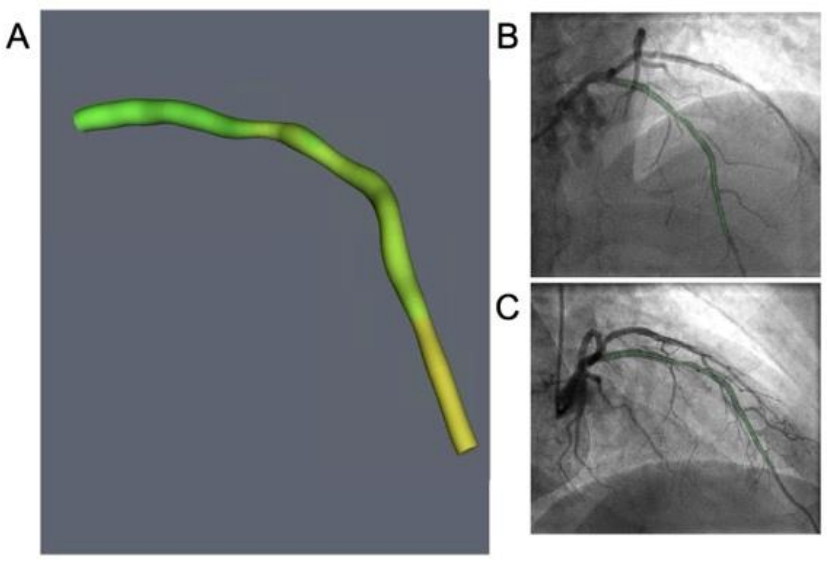

Inlet diameter: 2.74 mm
Outlet diameter: 1.92 mm
3D QCA: 31 %
FFR: 74/89 mmHg
Hyp inlet Q: 224 mL/min
Hyp outlet Q: 102 mL/min

Case E18

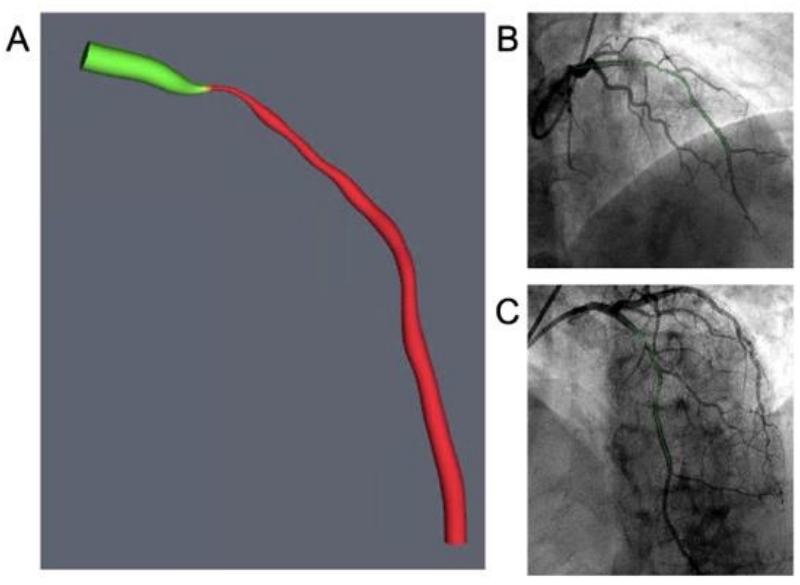

Inlet diameter: 2.70 mm
Outlet diameter: 1.94 mm
3D QCA: 76 %
FFR: 45/107 mmHg
Hyp inlet Q: 30 mL/min
Hyp outlet Q: 14 mL/min

Case E19

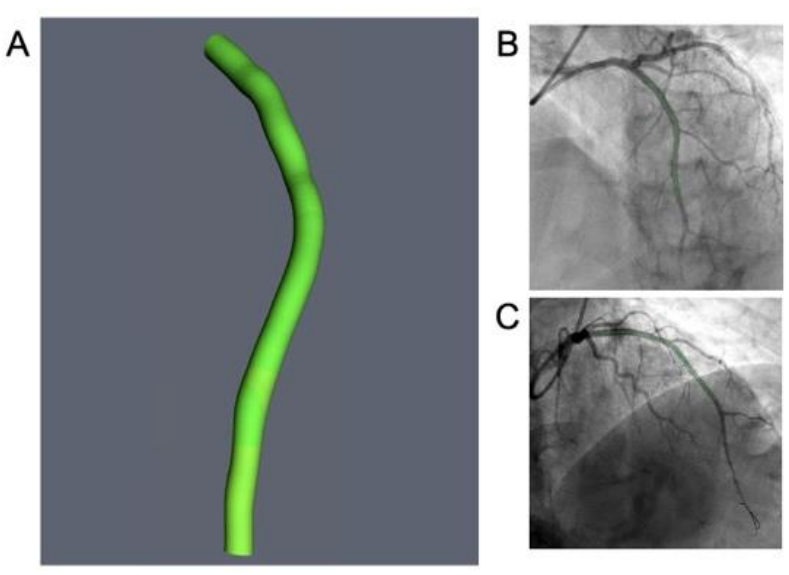

Inlet diameter: 2.60 mm
Outlet diameter: 2.11 mm
3D QCA: 0 %
FFR: 94/100 mmHg
Hyp inlet Q: 159 mL/min
Hyp outlet Q: 97 mL/min

Case E20

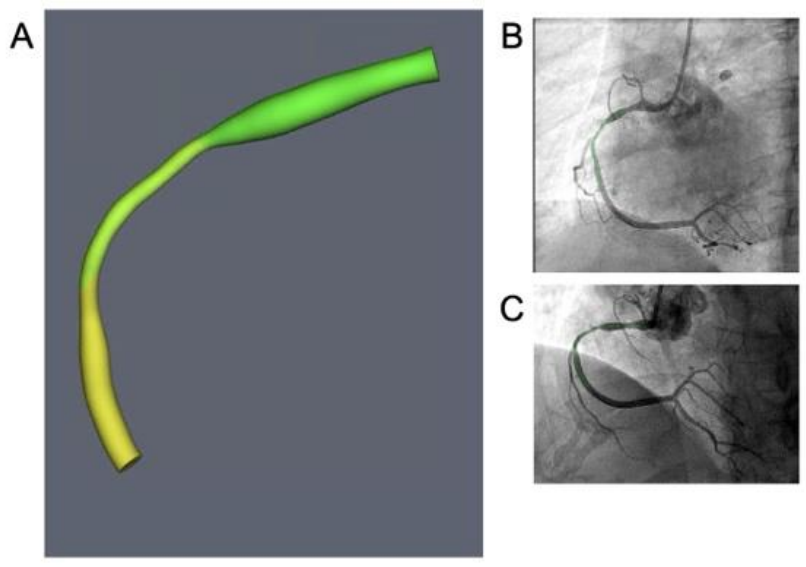

Inlet diameter: 2.74 mm
Outlet diameter: 2.51 mm
3D QCA: 55 %
FFR: 94/112 mmHg
Hyp inlet Q: 121 mL/min
Hyp outlet Q: 105 mL/min

Case E21

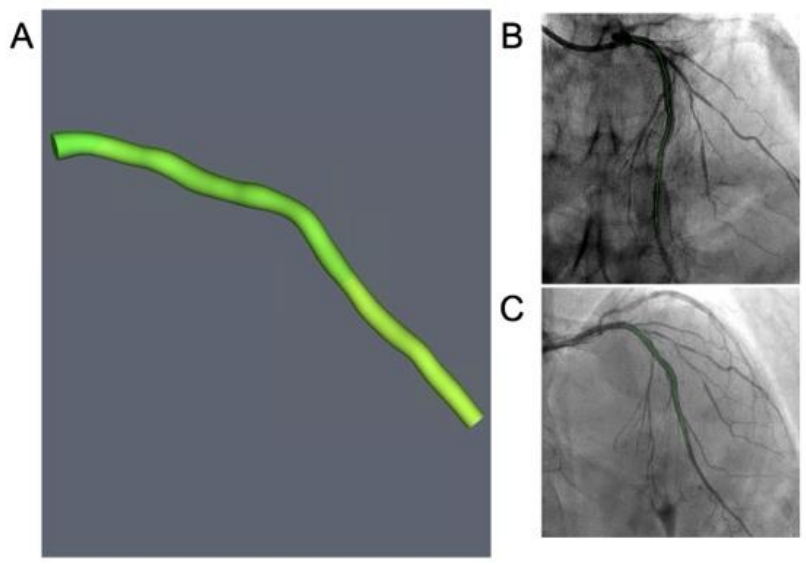

Inlet diameter: 3.10 mm
Outlet diameter: 2.08 mm
3D QCA: 11 %
FFR: 99/113 mmHg
Hyp inlet Q: 348 mL/min
Hyp outlet Q: 134 mL/min

Case E22

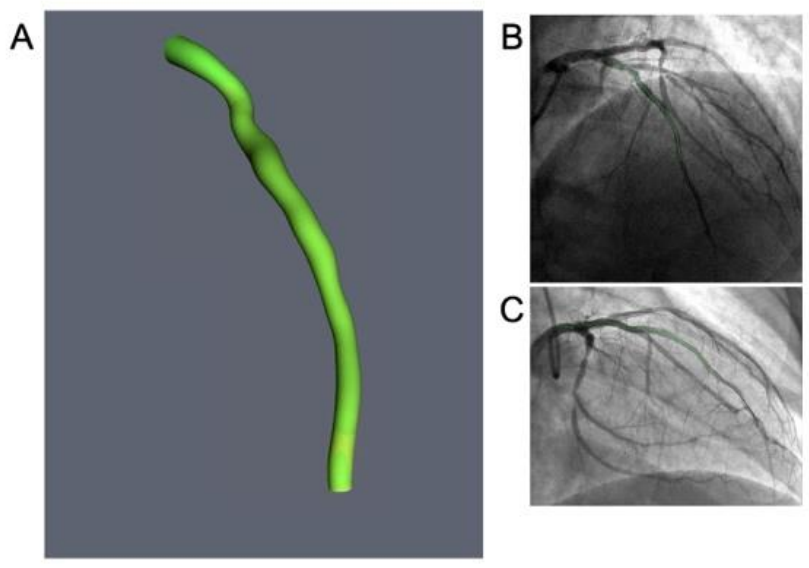

Inlet diameter: 2.92 mm
Outlet diameter: 1.80 mm
3D QCA: 0 %
FFR: 104/116 mmHg
Hyp inlet Q: 273 mL/min
Hyp outlet Q: 89 mL/min

Case E23

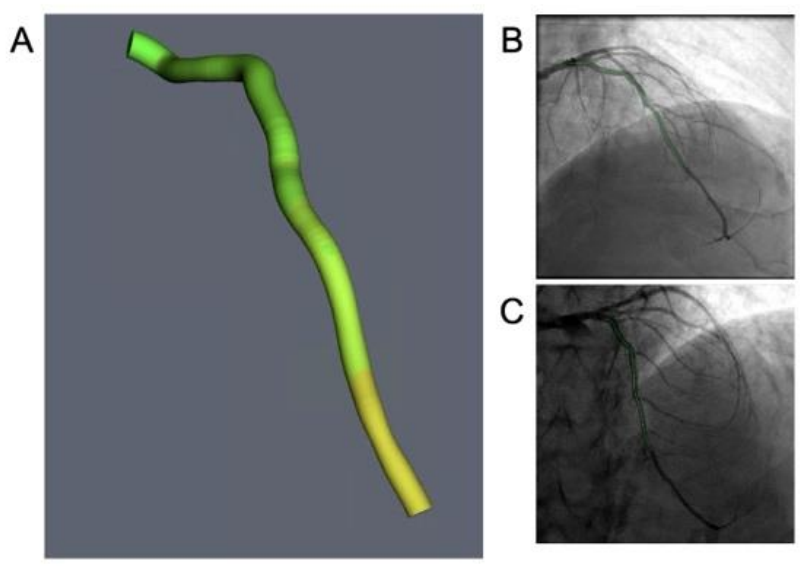

Inlet diameter: 2.62 mm
Outlet diameter: 1.80 mm
3D QCA: 0 %
FFR: 80/99 mmHg
Hyp inlet Q: 269 mL/min
Hyp outlet Q: 113 mL/min

Case E24

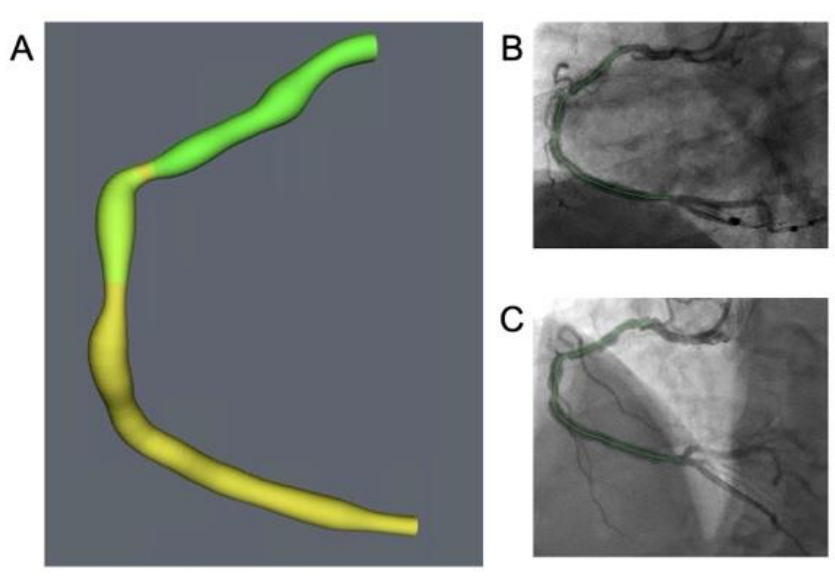

Inlet diameter: 2.53 mm
Outlet diameter: 1.93 mm
3D QCA: 52 %
FFR: 86/108 mmHg
Hyp inlet Q: 188 mL/min
Hyp outlet Q: 107 mL/min

Case E25

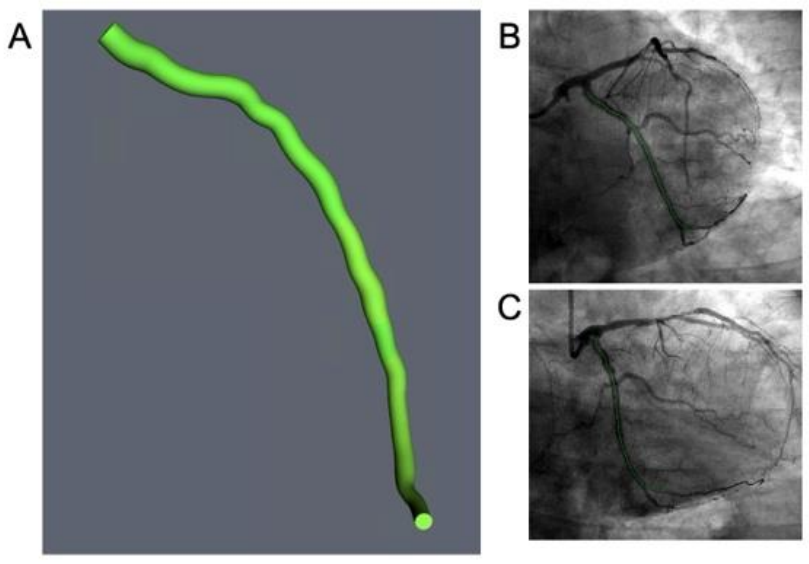

Inlet diameter: 2.87 mm
Outlet diameter: 1.46 mm
3D QCA: 0 %
FFR: 68/75 mmHg
Hyp inlet Q: 134 mL/min
Hyp outlet Q: 24 mL/min

Case E26

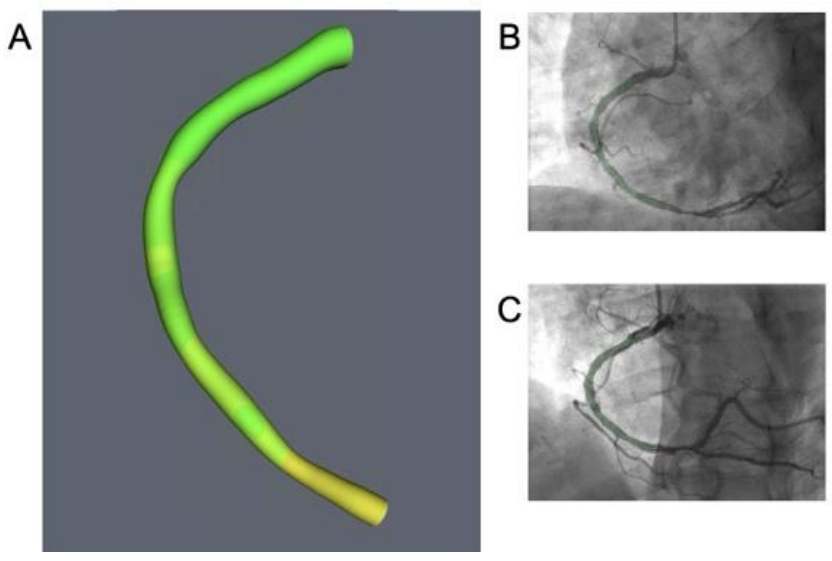

Inlet diameter: 4.10 mm
Outlet diameter: 2.89 mm
3D QCA: 26 %
FFR: 81/86 mmHg
Hyp inlet Q: 501 mL/min
Hyp outlet Q: 221 mL/min

Case E27

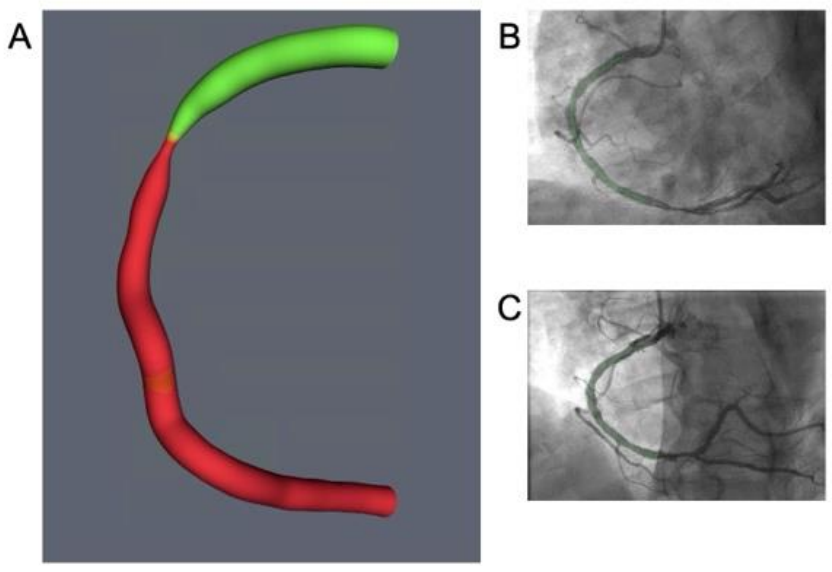

Inlet diameter: 3.65 mm
Outlet diameter: 3.34 mm
3D QCA: 67 %
FFR: 68/115 mmHg
Hyp inlet Q: 199 mL/min
Hyp outlet Q: 156 mL/min

Case E28

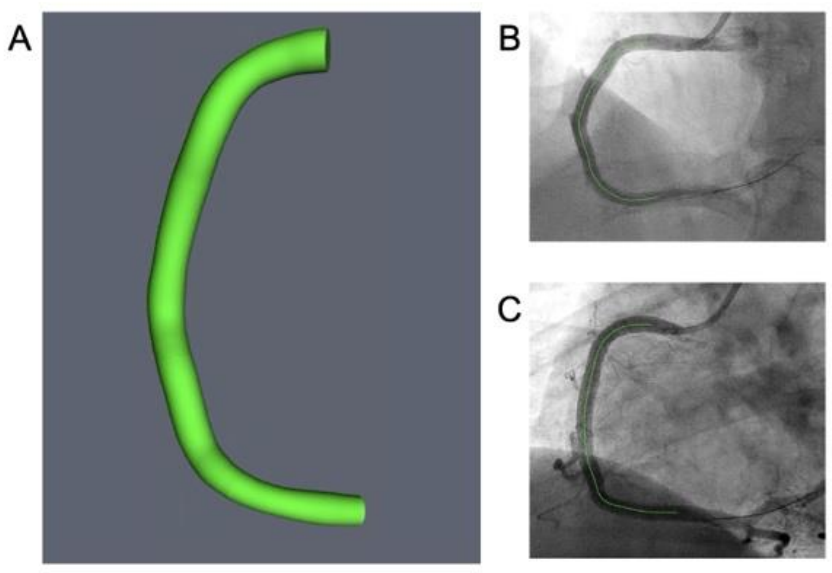

Inlet diameter: 4.09 mm
Outlet diameter: 2.92 mm
3D QCA: 0 %
FFR: 114/117 mmHg
Hyp inlet Q: 219 mL/min
Hyp outlet Q: 100 mL/min

Case E30

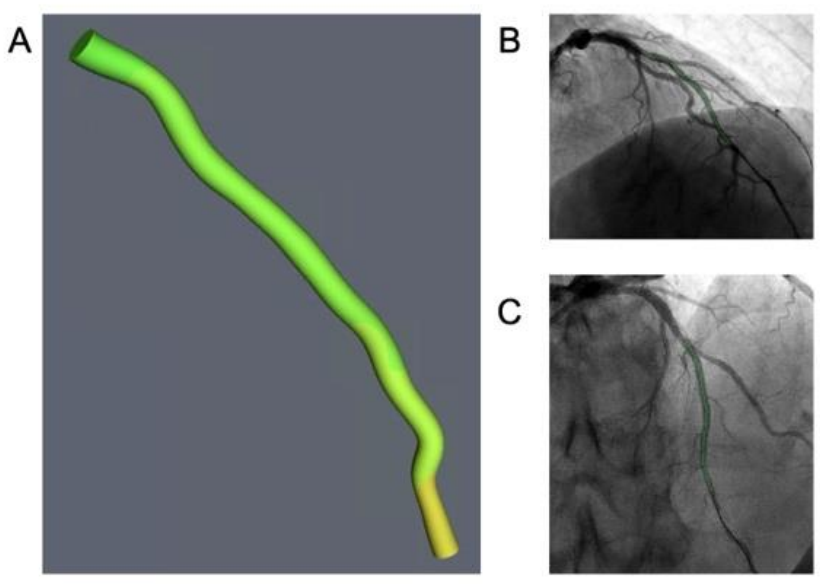

Inlet diameter: 2.63 mm
Outlet diameter: 1.87 mm
3D QCA: 0 %
FFR: 63/73 mmHg
Hyp inlet Q: 234 mL/min
Hyp outlet Q: 107 mL/min

Case E31

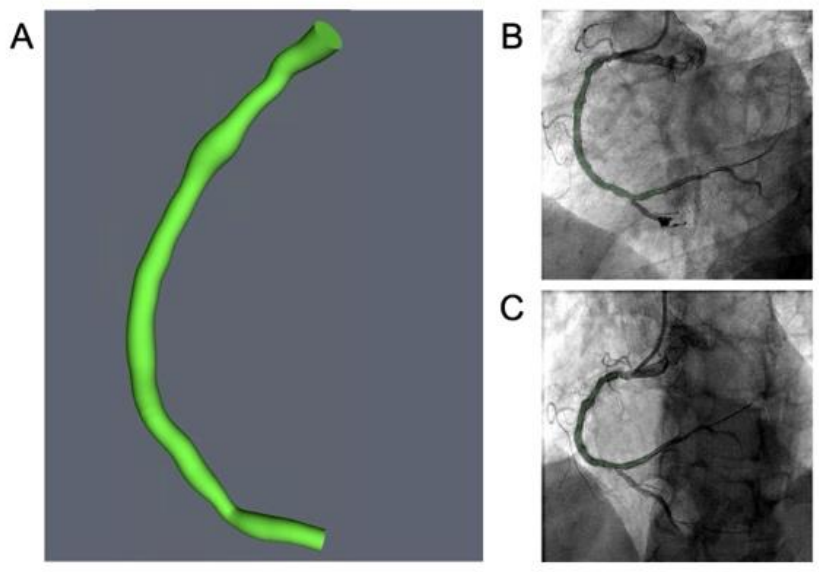

Inlet diameter: 3.98 mm
Outlet diameter: 2.49 mm
3D QCA: 37 %
FFR: 88/91 mmHg
Hyp inlet Q: 149 mL/min
Hyp outlet Q: 50 mL/min

Case E32

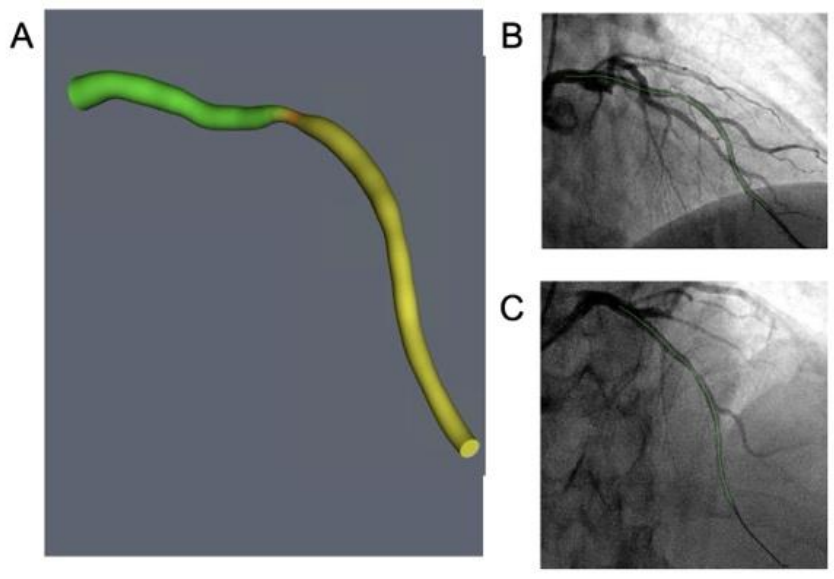

Inlet diameter: 4.04 mm
Outlet diameter: 1.74 mm
3D QCA: 40 %
FFR: 69/88 mmHg
Hyp inlet Q: 369 mL/min
Hyp outlet Q: 54 mL/min

Case E33

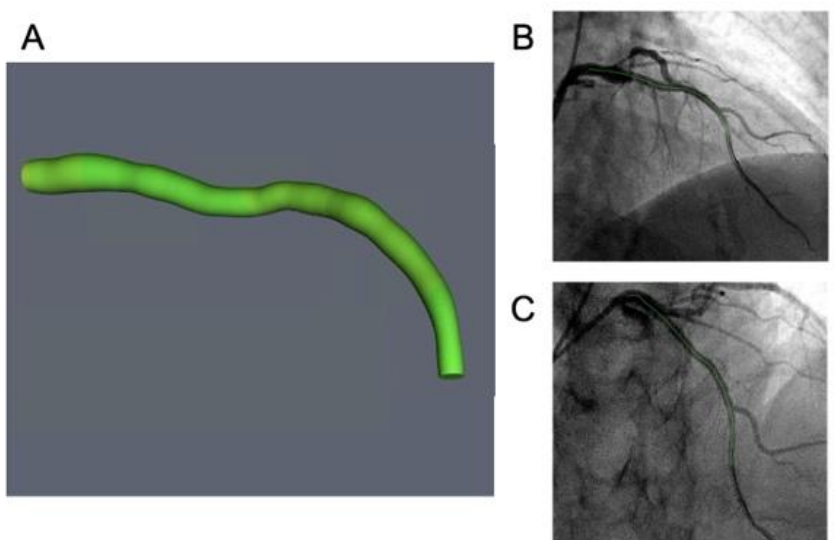

Inlet diameter: 3.27 mm
Outlet diameter: 1.87 mm
3D QCA: 0 %
FFR: 81/90 mmHg
Hyp inlet Q: 501 mL/min
Hyp outlet Q: 131 mL/min

Case E34

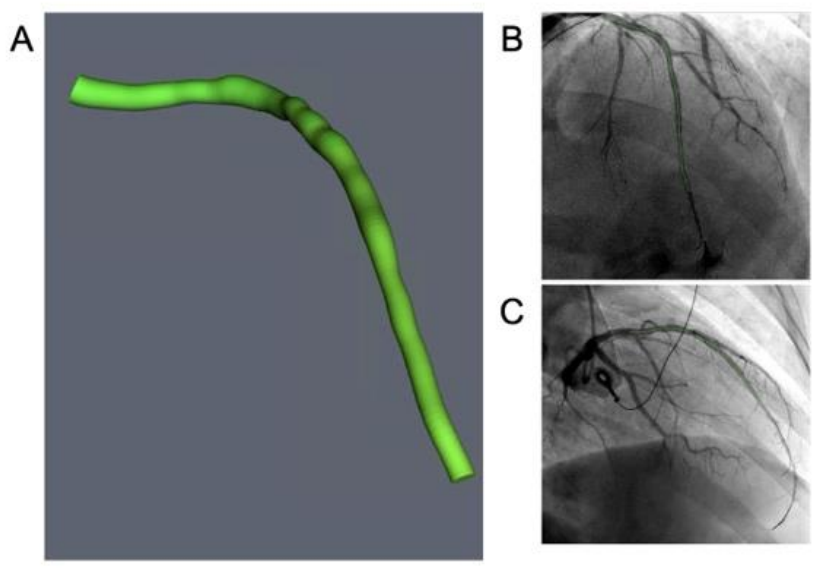

Inlet diameter: 3.08 mm
Outlet diameter: 1.82 mm
3D QCA: 24 %
FFR: 84/94 mmHg
Hyp inlet Q: 240 mL/min
Hyp outlet Q: 70 mL/min

Case E36

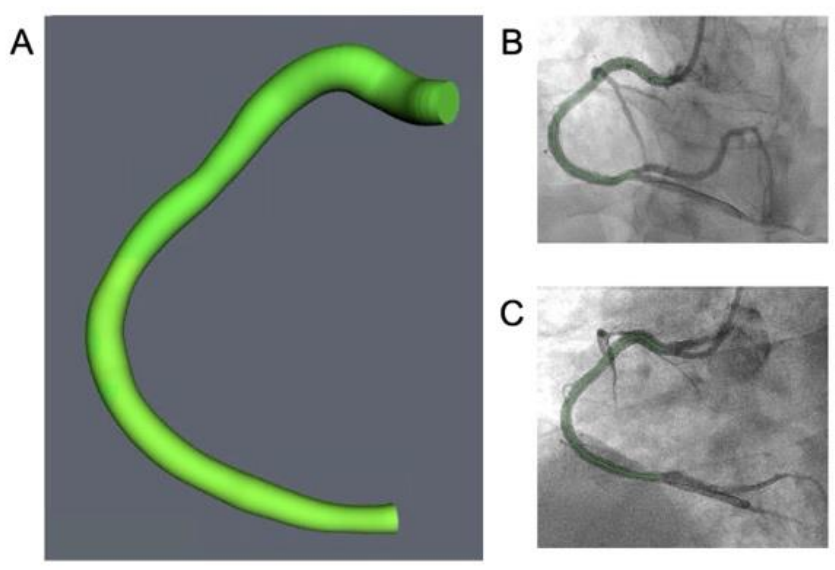

Inlet diameter: 3.36 mm
Outlet diameter: 2.99 mm
3D QCA: 15 %
FFR: 78/84 mmHg
Hyp inlet Q: 214 mL/min
Hyp outlet Q: 119 mL/min

Case E37

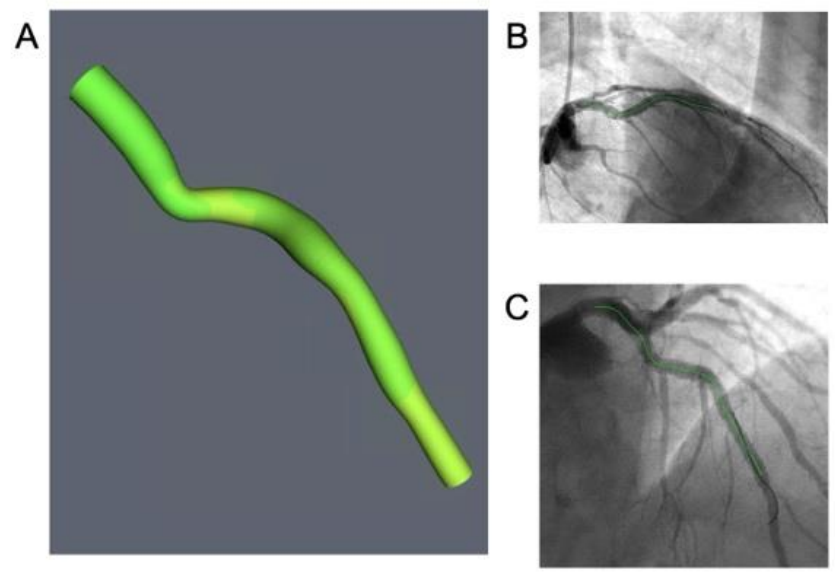

Inlet diameter: 3.66 mm
Outlet diameter: 3.23 mm
3D QCA: 20 %
FFR: 102/116 mmHg
Hyp inlet Q: 545 mL/min
Hyp outlet Q: 206 mL/min

Case E38

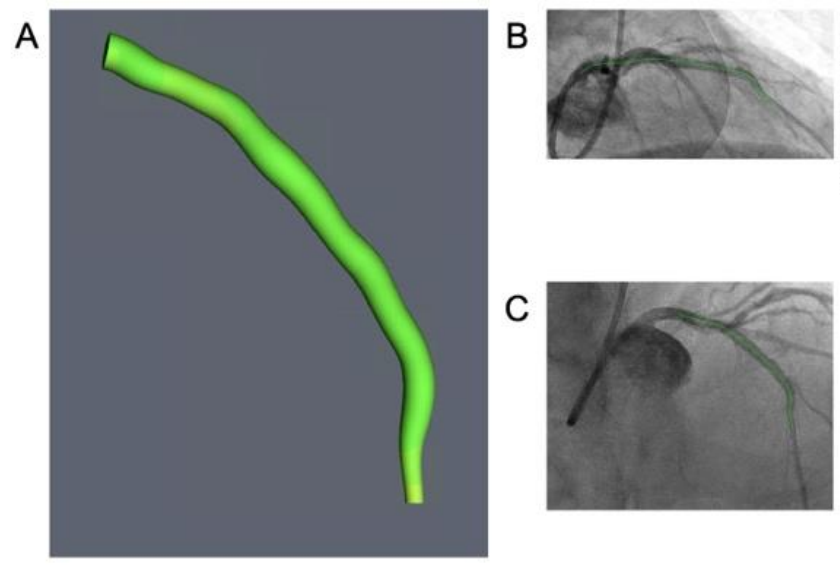

Inlet diameter: 2.87 mm
Outlet diameter: 1.30 mm
3D QCA: 0 %
FFR: 89/100 mmHg
Hyp inlet Q: 467 mL/min
Hyp outlet Q: 75 mL/min

Case E39

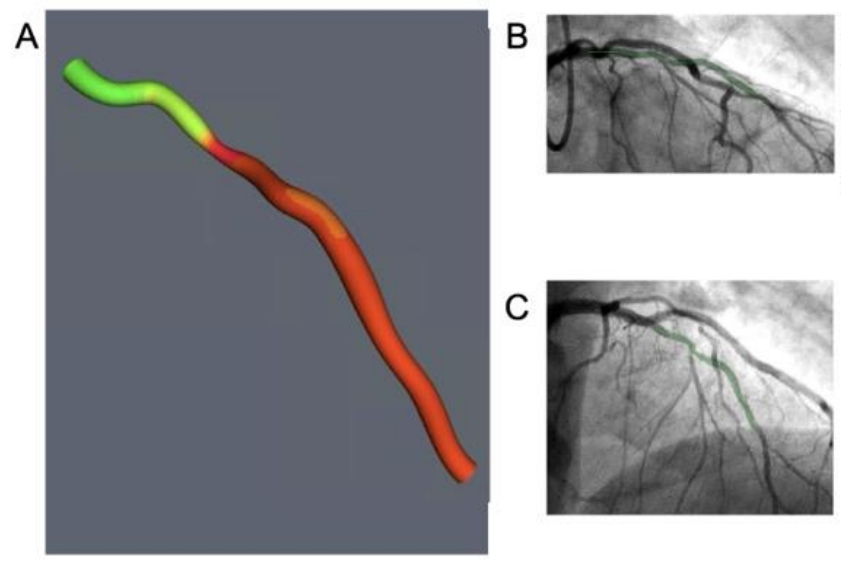

Inlet diameter: 2.02 mm
Outlet diameter: 1.66 mm
3D QCA: 34 %
FFR: 52/81 mmHg
Hyp inlet Q: 125 mL/min
Hyp outlet Q: 79 mL/min

Case E40

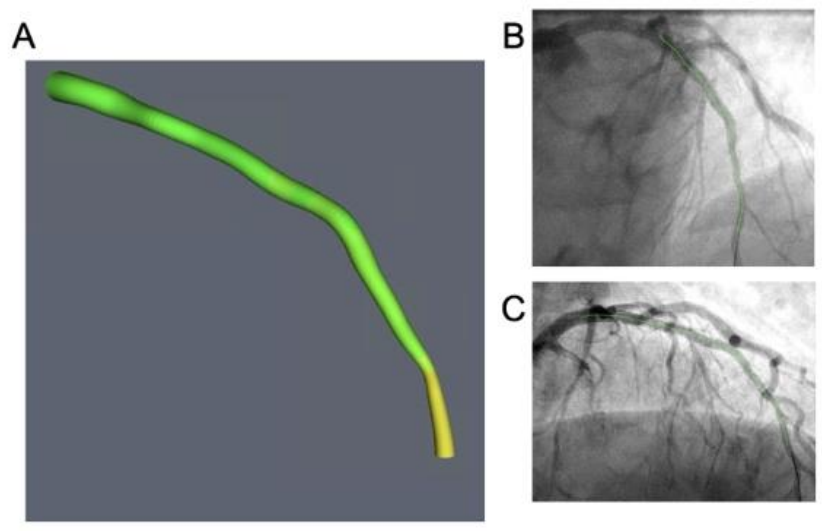

Inlet diameter: 3.28 mm
Outlet diameter: 1.89 mm
3D QCA: 32 %
FFR: 67/82 mmHg
Hyp inlet Q: 267 mL/min
Hyp outlet Q: 72 mL/min

Case E41

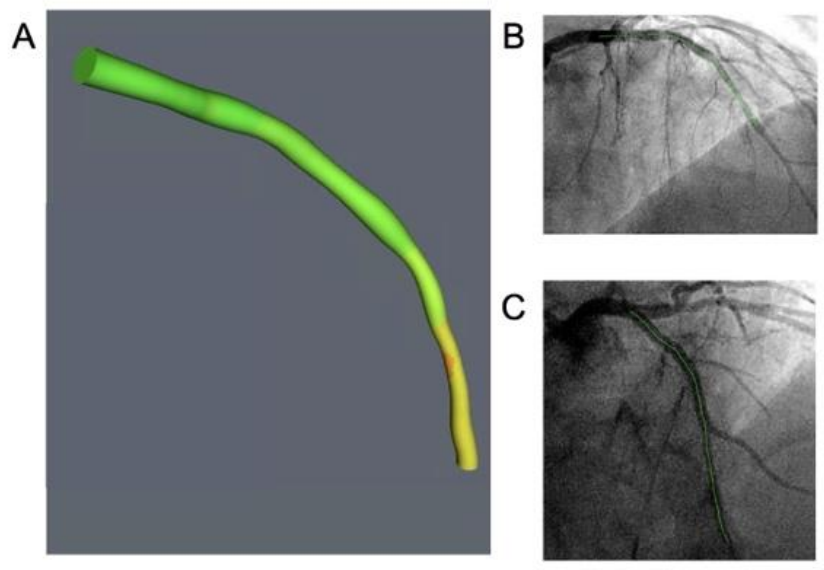

Inlet diameter: 3.30 mm
Outlet diameter: 2.16 mm
3D QCA: 17 %
FFR: 72/90 mmHg
Hyp inlet Q: 400 mL/min
Hyp outlet Q: 149 mL/min

Case E42

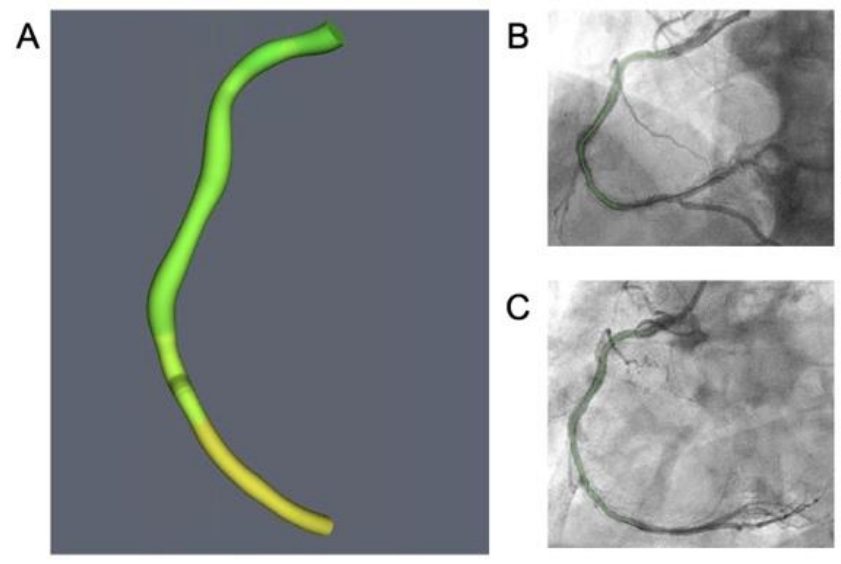

Inlet diameter: 2.88 mm
Outlet diameter: 2.09 mm
3D QCA: 27 %
FFR: 75/91 mmHg
Hyp inlet Q: 214 mL/min
Hyp outlet Q: 106 mL/min

Case E44

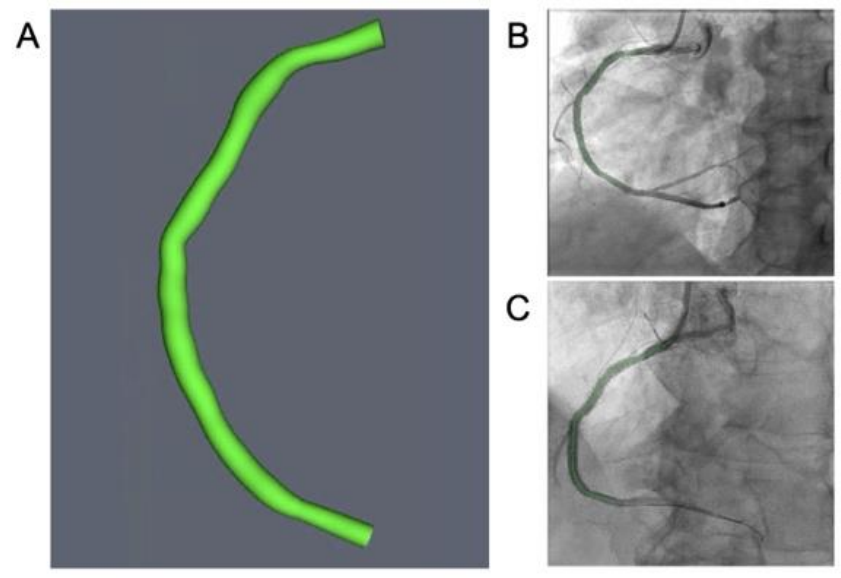

Inlet diameter: 2.91 mm
Outlet diameter: 2.33 mm
3D QCA: 29 %
FFR: 98/101 mmHg
Hyp inlet Q: 84 mL/min
Hyp outlet Q: 52 mL/min

Case E45

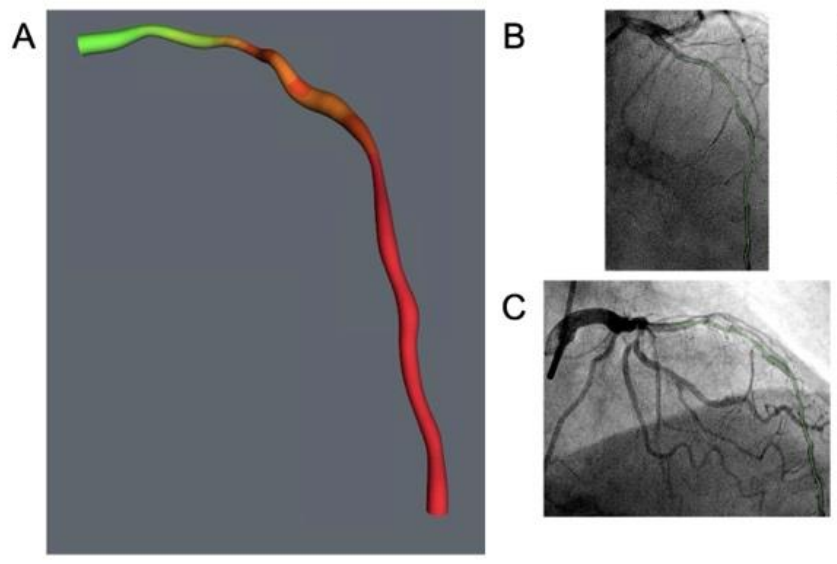

Inlet diameter: 2.45 mm
Outlet diameter: 1.82 mm
3D QCA: 57 %
FFR: 57/98 mmHg
Hyp inlet Q: 87 mL/min
Hyp outlet Q: 46 mL/min

Case E46

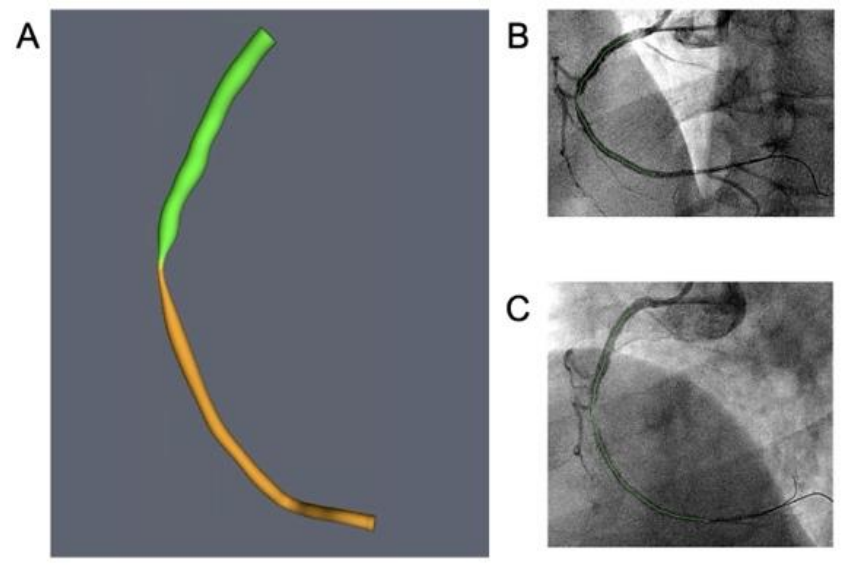

Inlet diameter: 2.74 mm
Outlet diameter: 2.03 mm
3D QCA: 67 %
FFR: 69/90 mmHg
Hyp inlet Q: 54 mL/min
Hyp outlet Q: 26 mL/min

Case E47

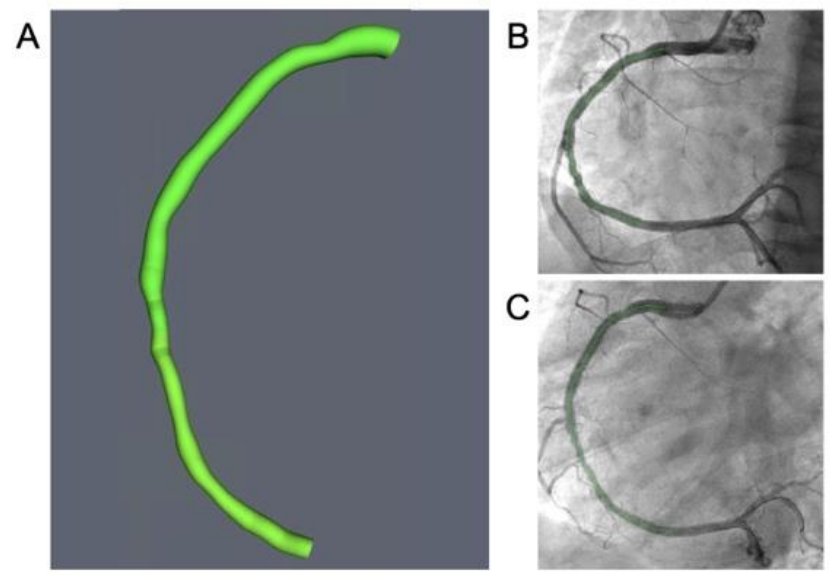

Inlet diameter: 3.92 mm
Outlet diameter: 2.66 mm
3D QCA: 25 %
FFR: 82/94 mmHg
Hyp inlet Q: 194 mL/min
Hyp outlet Q: 82 mL/min

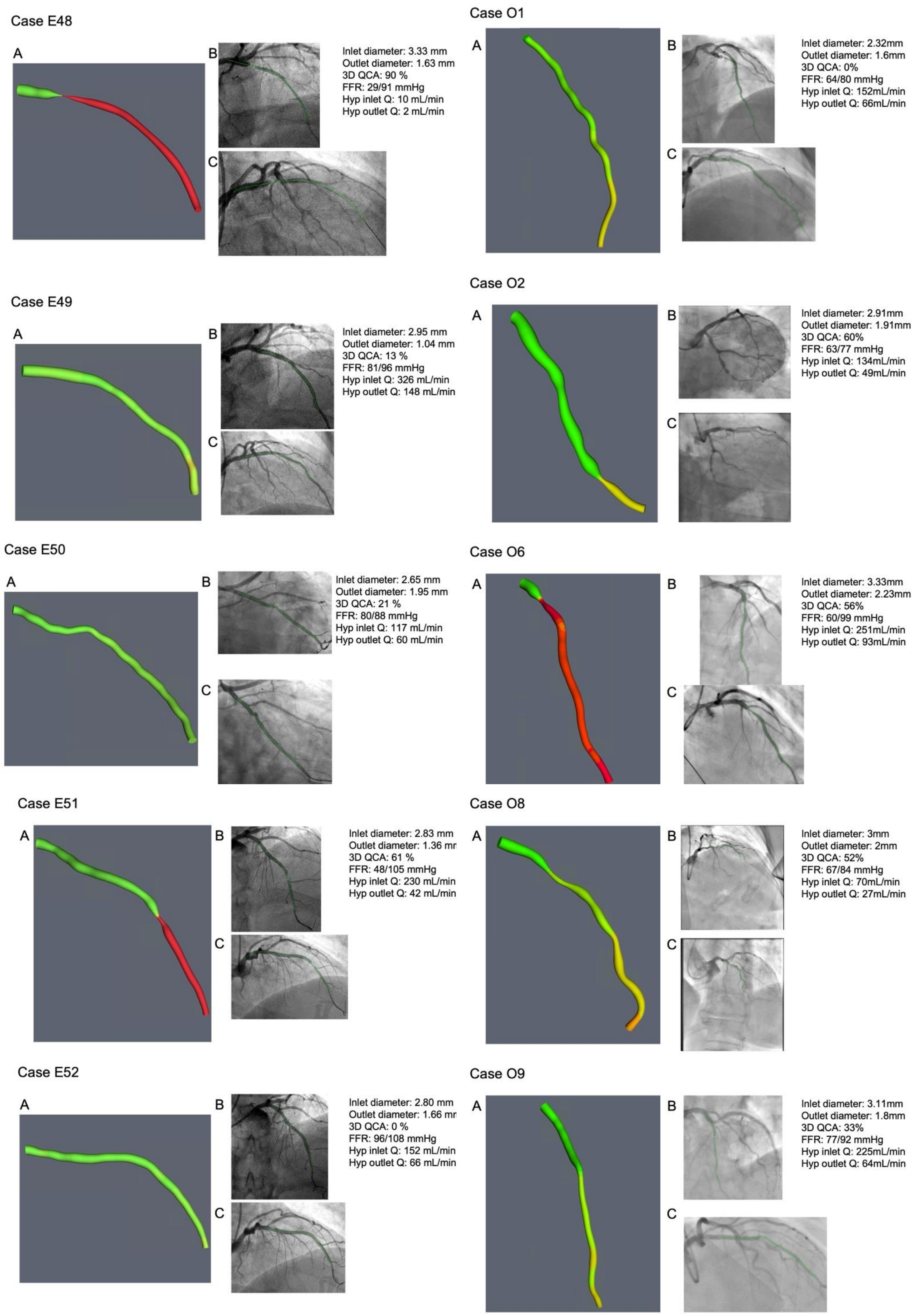

Case E48
A
B
C
Inlet diameter: 3.33 mm
Outlet diameter: 1.63 mm
3D QCA: 90 %
FFR: 29/91 mmHg
Hyp inlet Q: 10 mL/min
Hyp outlet Q: 2 mL/min
Case O1
A
B
C
Inlet diameter: 2.32mm
Outlet diameter: 1.6mm
3D QCA: 0%
FFR: 64/80 mmHg
Hyp inlet Q: 152mL/min
Hyp outlet Q: 66mL/min
Case E49
A
B
C
Inlet diameter: 2.95 mm
Outlet diameter: 1.04 mm
3D QCA: 13 %
FFR: 81/96 mmHg
Hyp inlet Q: 326 mL/min
Hyp outlet Q: 148 mL/min
Case O2
A
B
C
Inlet diameter: 2.91mm
Outlet diameter: 1.91mm
3D QCA: 60%
FFR: 63/77 mmHg
Hyp inlet Q: 134mL/min
Hyp outlet Q: 49mL/min
Case E50
A
B
C
Inlet diameter: 2.65 mm
Outlet diameter: 1.95 mm
3D QCA: 21 %
FFR: 80/88 mmHg
Hyp inlet Q: 117 mL/min
Hyp outlet Q: 60 mL/min
Case O6
A
B
C
Inlet diameter: 3.33mm
Outlet diameter: 2.23mm
3D QCA: 56%
FFR: 60/99 mmHg
Hyp inlet Q: 251mL/min
Hyp outlet Q: 93mL/min
Case E51
A
B
C
Inlet diameter: 2.83 mm
Outlet diameter: 1.36 mr
3D QCA: 61 %
FFR: 48/105 mmHg
Hyp inlet Q: 230 mL/min
Hyp outlet Q: 42 mL/min
Case O8
A
B
C
Inlet diameter: 3mm
Outlet diameter: 2mm
3D QCA: 52%
FFR: 67/84 mmHg
Hyp inlet Q: 70mL/min
Hyp outlet Q: 27mL/min
Case E52
A
B
C
Inlet diameter: 2.80 mm
Outlet diameter: 1.66 mr
3D QCA: 0 %
FFR: 96/108 mmHg
Hyp inlet Q: 152 mL/min
Hyp outlet Q: 66 mL/min
Case O9
A
B
C
Inlet diameter: 3.11mm
Outlet diameter: 1.8mm
3D QCA: 33%
FFR: 77/92 mmHg
Hyp inlet Q: 225mL/min
Hyp outlet Q: 64mL/min

Case O11

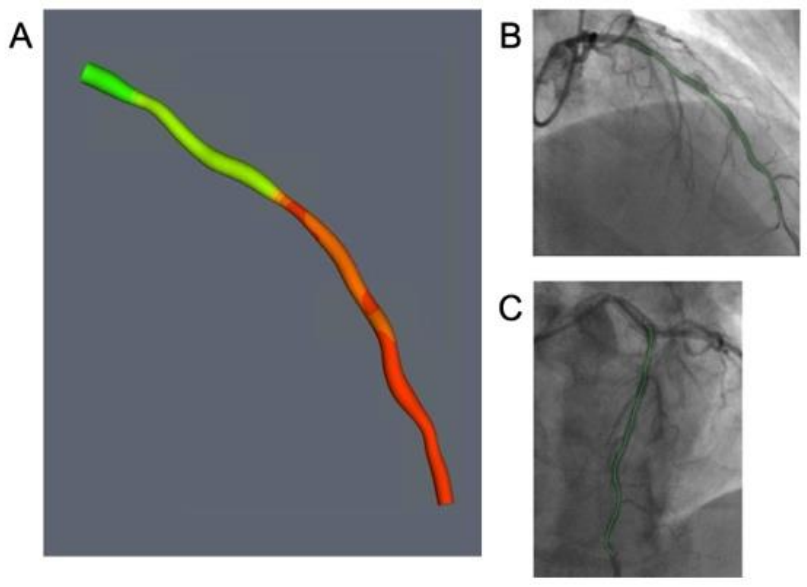

Inlet diameter: 2.96mm
Outlet diameter: 1.88mm
3D QCA: 37%
FFR: 67/102 mmHg
Hyp inlet Q: 317mL/min
Hyp outlet Q: 113mL/min

Case O12

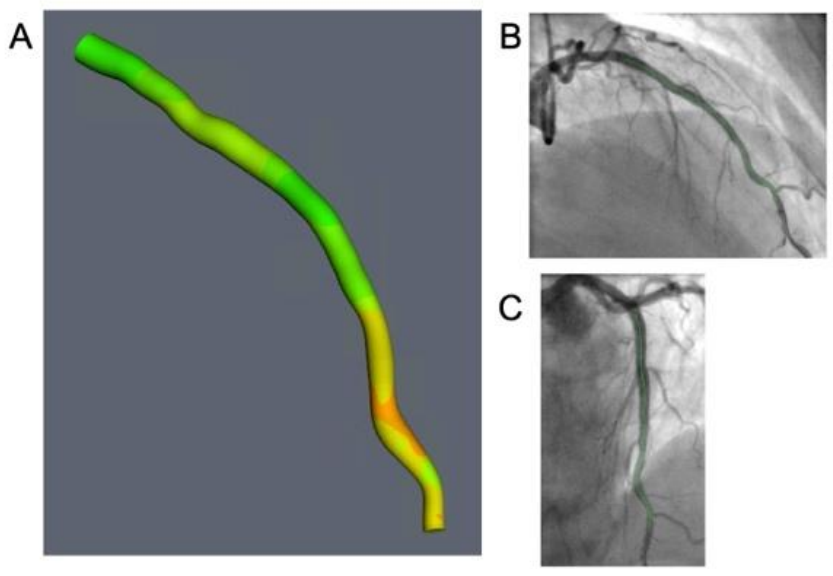

Inlet diameter: 3.29mm
Outlet diameter: 1.84mm
3D QCA: 17%
FFR: 81/105 mmHg
Hyp inlet Q: 714mL/min
Hyp outlet Q: 175mL/min

Case O13

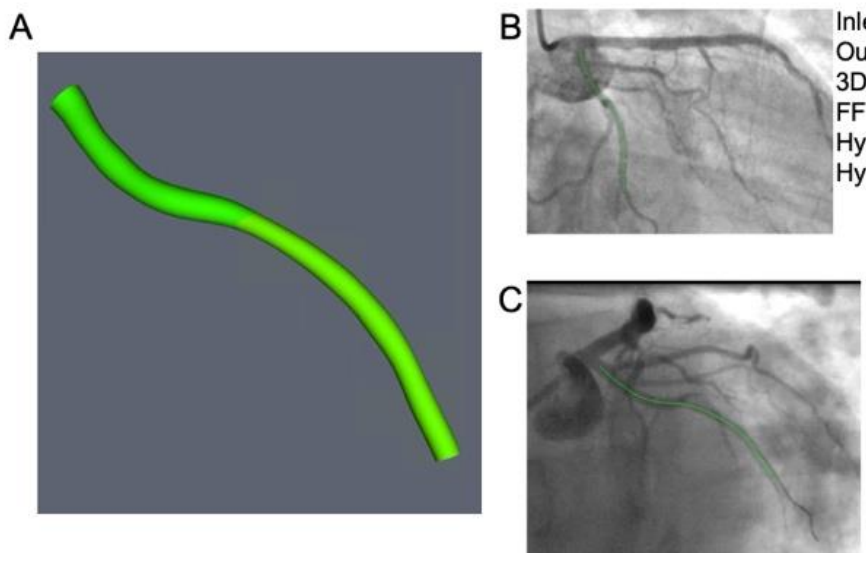

Inlet diameter: 3.24mm
Outlet diameter: 2.15mm
3D QCA: 17%
FFR: 76/84 mmHg
Hyp inlet Q: 264mL/min
Hyp outlet Q: 105mL/min

Case O14

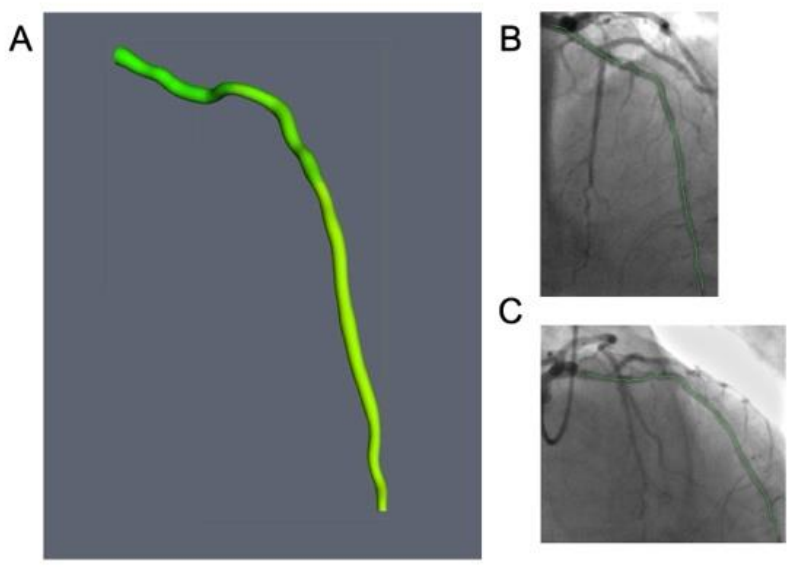

Inlet diameter: 3.07mm
Outlet diameter: 1.33mm
3D QCA: 16%
FFR: 78/91 mmHg
Hyp inlet Q: 170mL/min
Hyp outlet Q: 25mL/min

Case O15

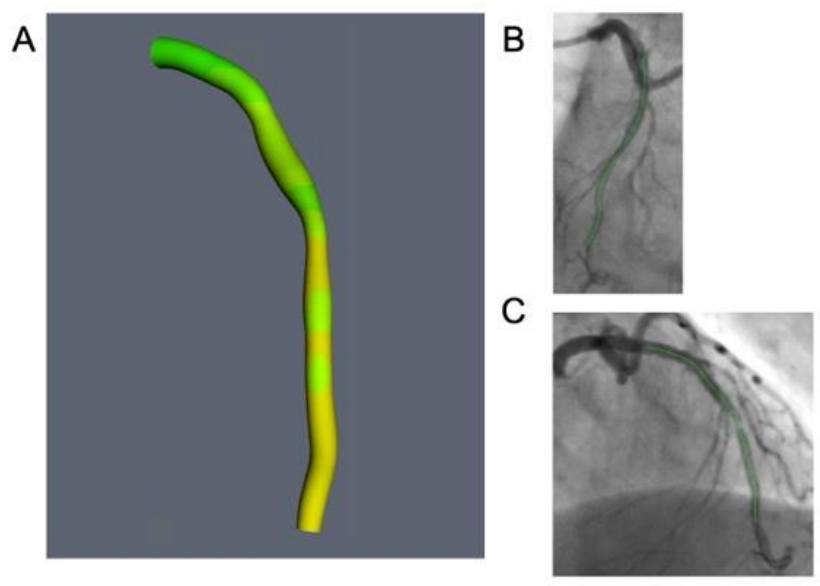

Inlet diameter: 2.89mm
Outlet diameter: 1.85mm
3D QCA: 27%
FFR: 80/97 mmHg
Hyp inlet Q: 344mL/min
Hyp outlet Q: 134mL/min

Case O16

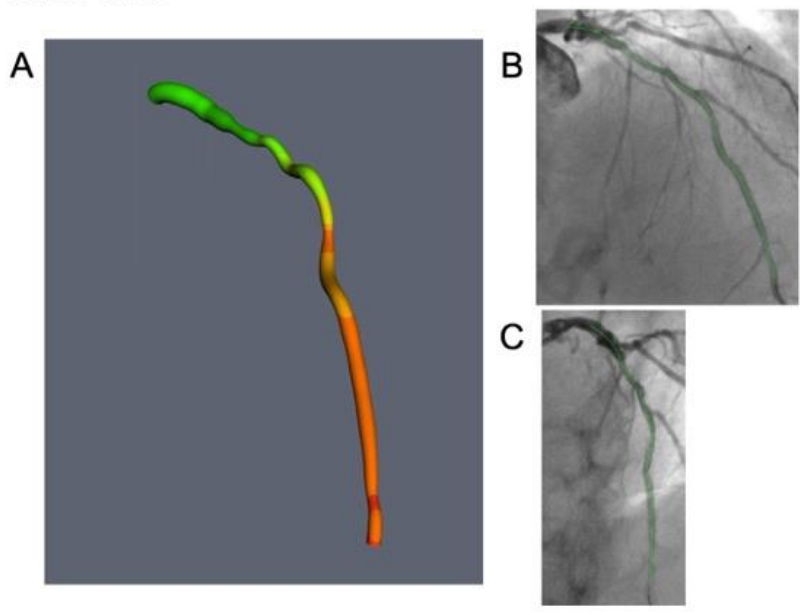

Inlet diameter: 3.36mm
Outlet diameter: 1.82mm
3D QCA: 37%
FFR: 46/66 mmHg
Hyp inlet Q: 178mL/min
Hyp outlet Q: 42mL/min

Case O17

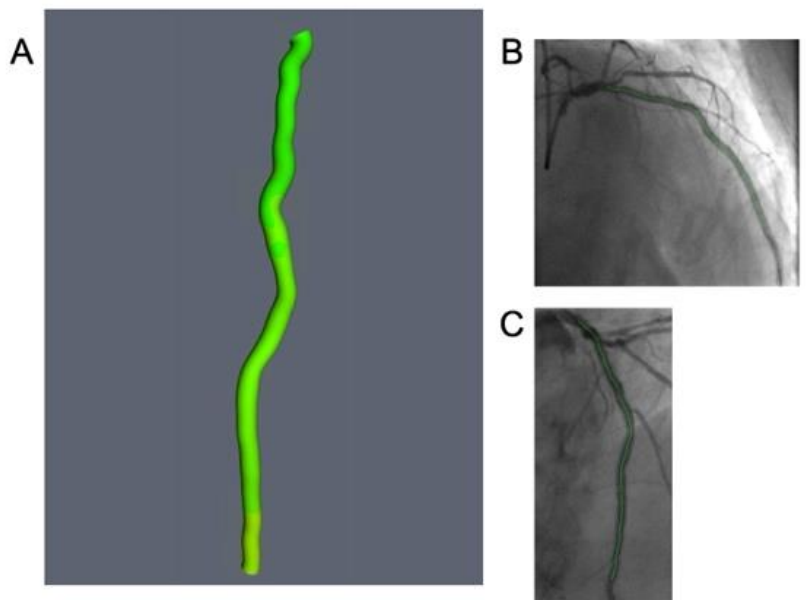

Inlet diameter: 3.2mm
Outlet diameter: 2.67mm
3D QCA: 19%
FFR: 72/81 mmHg
Hyp inlet Q: 219mL/min
Hyp outlet Q: 145mL/min

Case O18

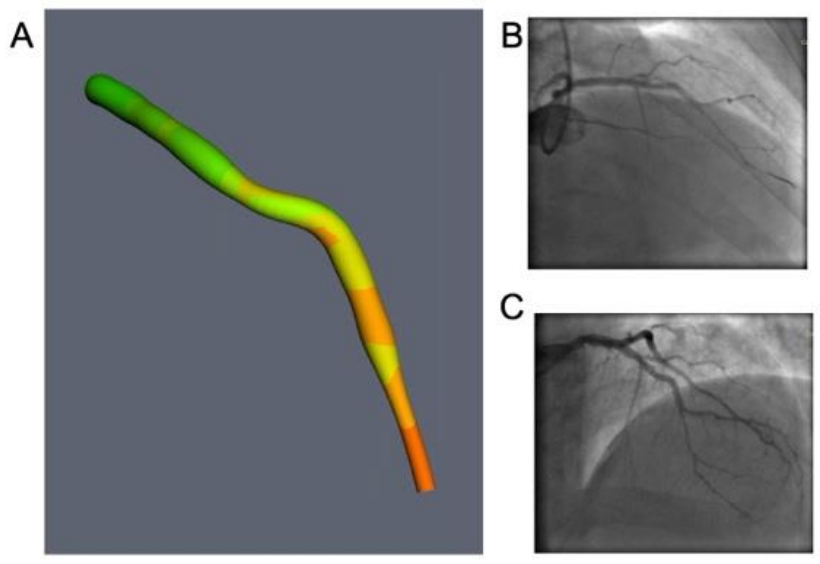

Inlet diameter: 2.66mm
Outlet diameter: 1.32mm
3D QCA: 22%
FFR: 64/91 mmHg
Hyp inlet Q: 496mL/min
Hyp outlet Q: 95mL/min

Case O21

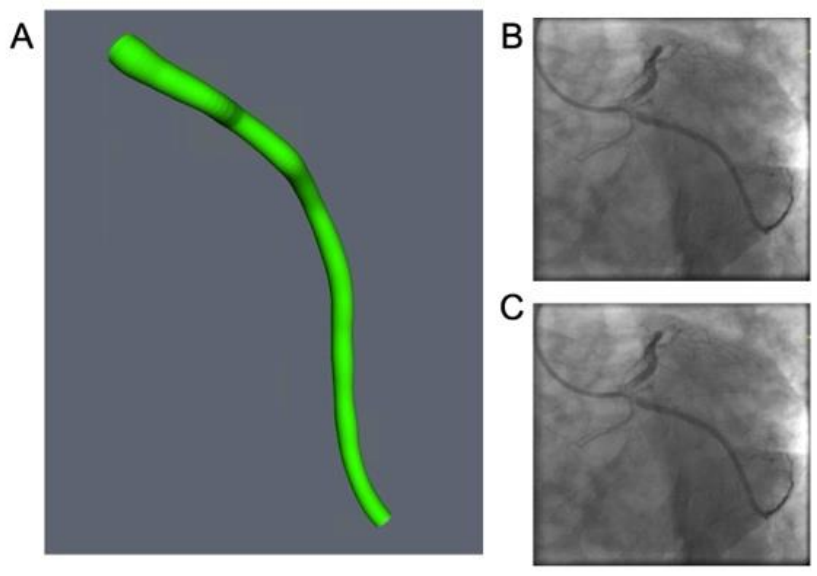

Inlet diameter: 3.21mm
Outlet diameter: 2.13mm
3D QCA: 0%
FFR: 83/87 mmHg
Hyp inlet Q: 114mL/min
Hyp outlet Q: 43mL/min

Case O22

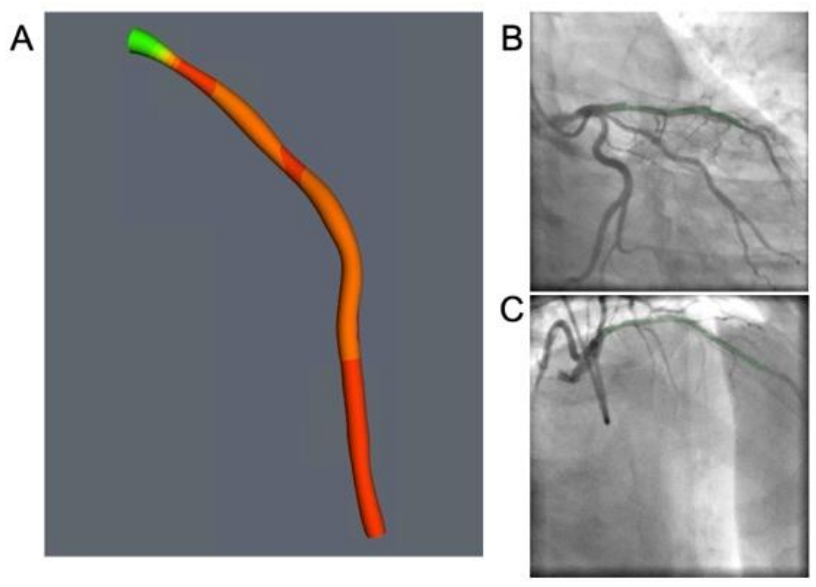

Inlet diameter: 2.81mm
Outlet diameter: 1.97mm
3D QCA: 42%
FFR: 54/83 mmHg
Hyp inlet Q: 269mL/min
Hyp outlet Q: 131mL/min

Case O23

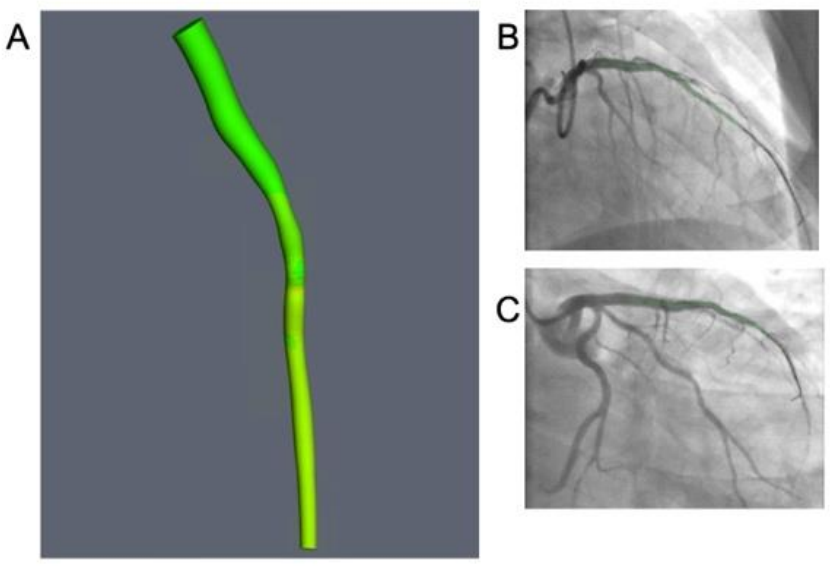

Inlet diameter: 3.59mm
Outlet diameter: 1.58mm
3D QCA: 0%
FFR: 78/89 mmHg
Hyp inlet Q: 362mL/min
Hyp outlet Q: 56mL/min

Case O28

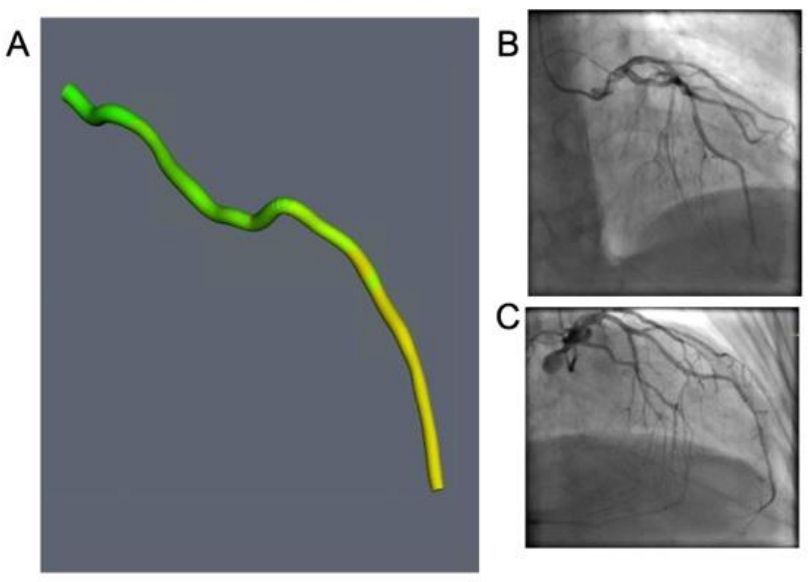

Inlet diameter: 2.49mm
Outlet diameter: 1.39mm
3D QCA: 21%
FFR: 87/106 mmHg
Hyp inlet Q: 165mL/min
Hyp outlet Q: 46mL/min

Case O29

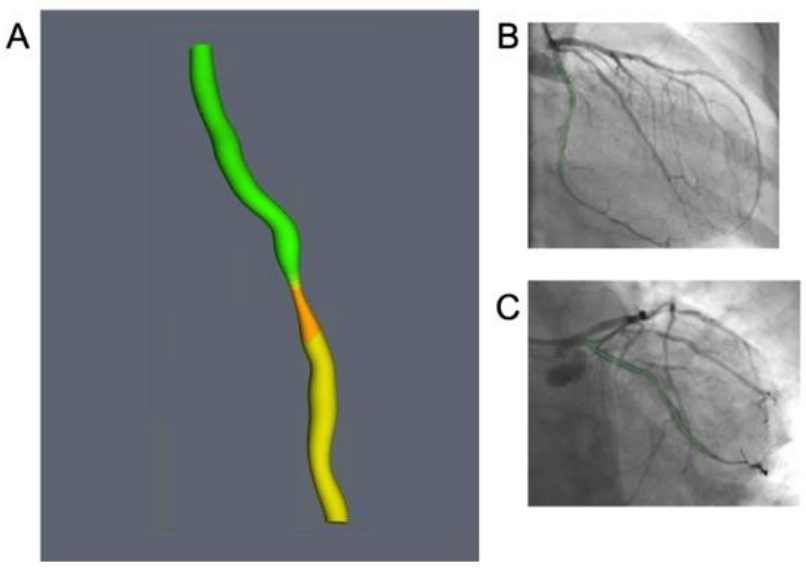

Inlet diameter: 2.18mm
Outlet diameter: 2.13mm
3D QCA: 60%
FFR: 72/94 mmHg
Hyp inlet Q: 91mL/min
Hyp outlet Q: 86mL/min

Case O30

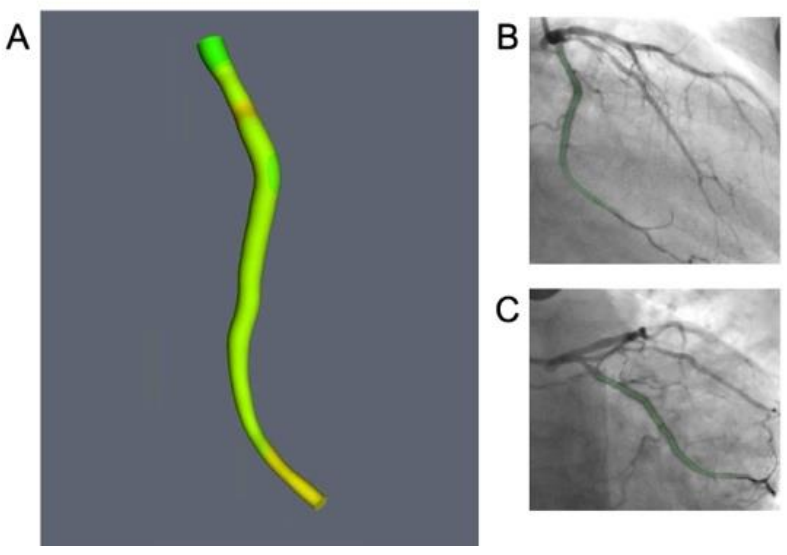

Inlet diameter: 3.1mm
Outlet diameter: 1.79mm
3D QCA: 0%
FFR: 87/106 mmHg
Hyp inlet Q: 419mL/min
Hyp outlet Q: 121mL/min

Case O31

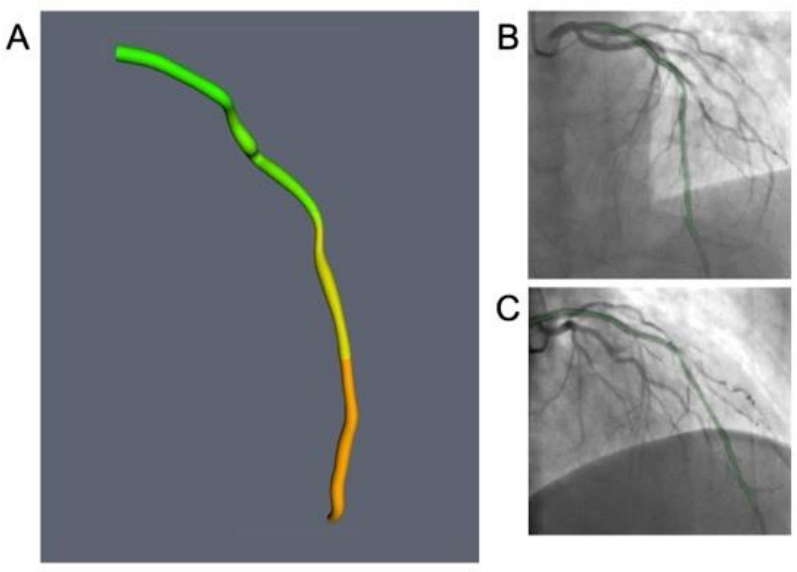

Inlet diameter: 2.71mm
Outlet diameter: 1.78mm
3D QCA: 44%
FFR: 64/82 mmHg
Hyp inlet Q: 140mL/min
Hyp outlet Q: 53mL/min

Case O32

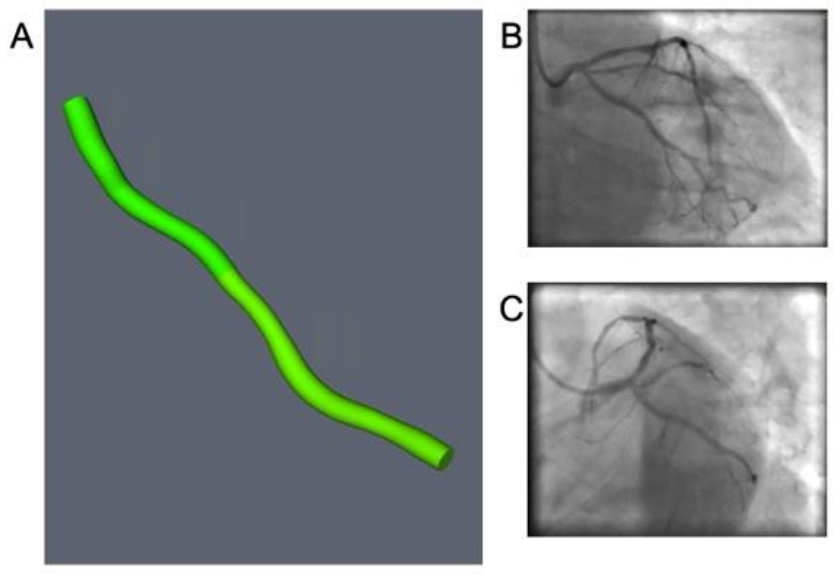

Inlet diameter: 2.36mm
Outlet diameter: 2.3mm
3D QCA: 0%
FFR: 74/81 mmHg
Hyp inlet Q: 118mL/min
Hyp outlet Q: 109mL/min

Case O33

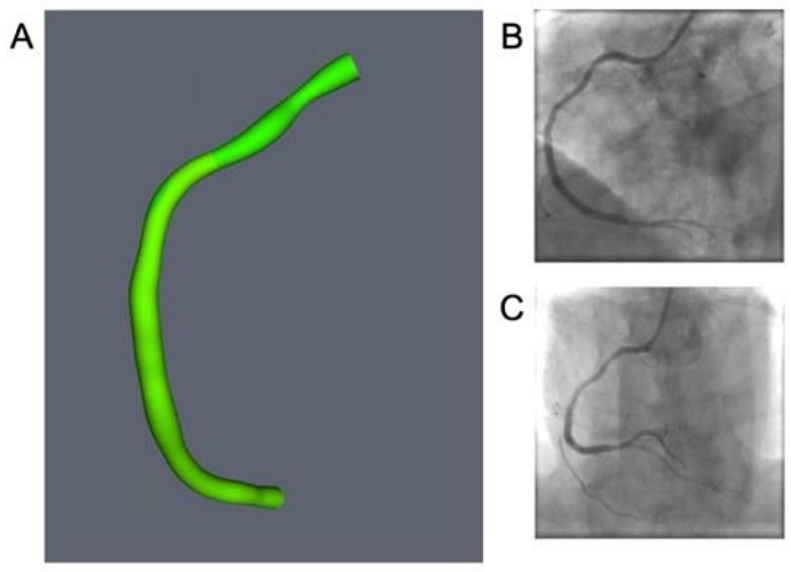

Inlet diameter: 2.88mm
Outlet diameter: 2.63mm
3D QCA: 36%
FFR: 65/70 mmHg
Hyp inlet Q: 93mL/min
Hyp outlet Q: 75mL/min

Case O34

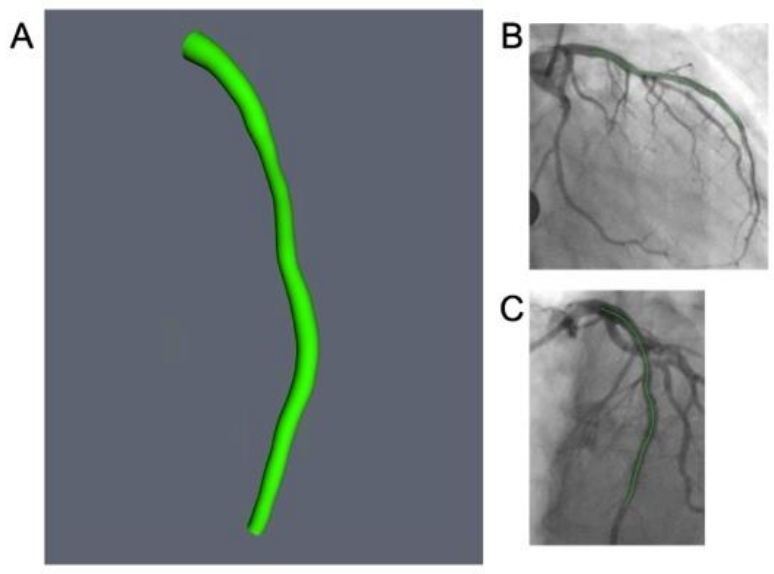

Inlet diameter: 4.23mm
Outlet diameter: 2.21mm
3D QCA: 27%
FFR: 97/99 mmHg
Hyp inlet Q: 133mL/min
Hyp outlet Q: 30mL/min

Case O35

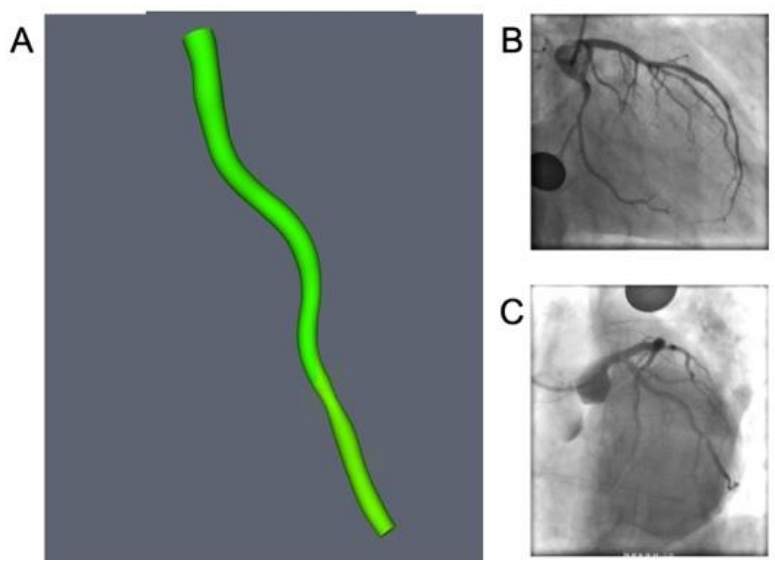

Inlet diameter: 3.27mm
Outlet diameter: 2.11mm
3D QCA: 25%
FFR: 99/107 mmHg
Hyp inlet Q: 236mL/min
Hyp outlet Q: 85mL/min

Case O37

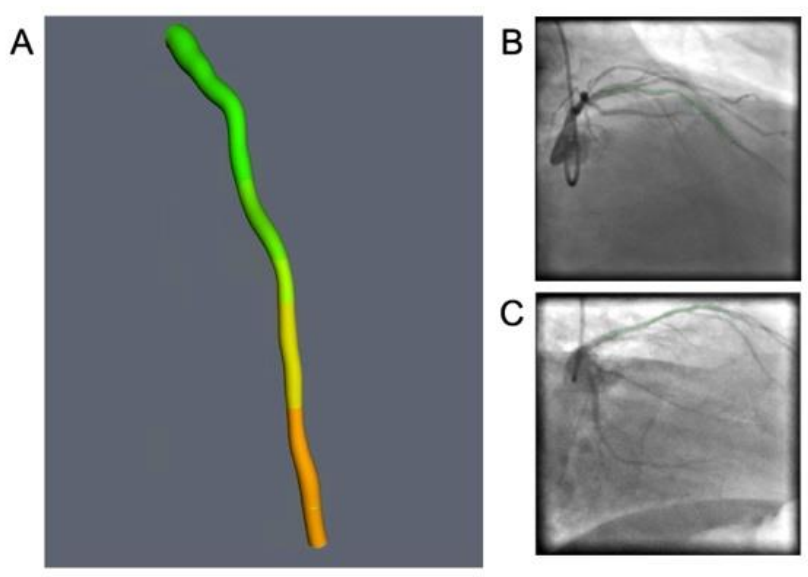

Inlet diameter: 2.98mm
Outlet diameter: 2.07mm
3D QCA: 20%
FFR: 75/97 mmHg
Hyp inlet Q: 291mL/min
Hyp outlet Q: 121mL/min

Case O38

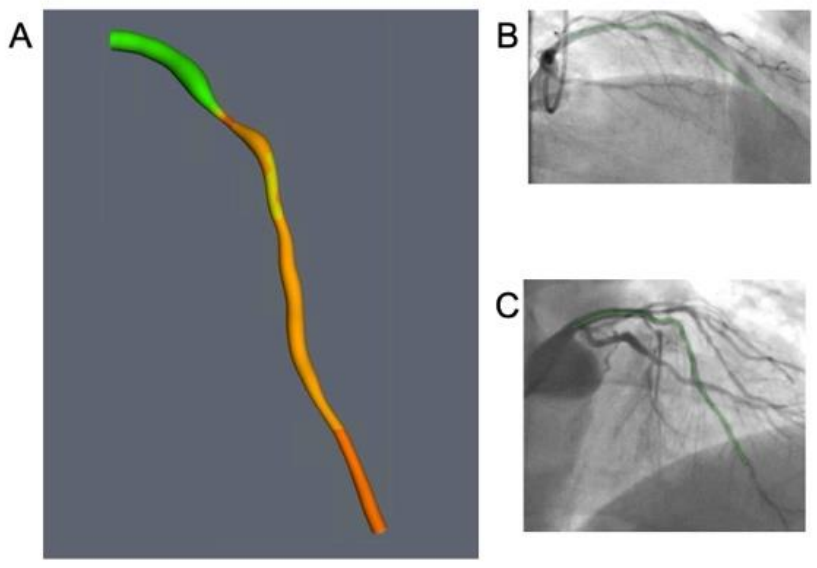

Inlet diameter: 3.01mm
Outlet diameter: 2.12mm
3D QCA: 56%
FFR: 77/106 mmHg
Hyp inlet Q: 174mL/min
Hyp outlet Q: 76mL/min

Case O39

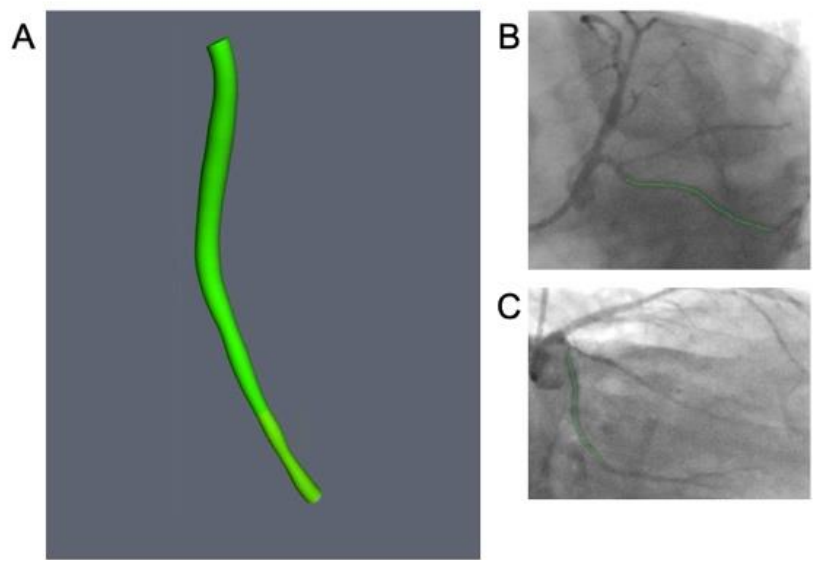

Inlet diameter: 1.73mm
Outlet diameter: 1.63mm
3D QCA: 26%
FFR: 96/105 mmHg
Hyp inlet Q: 53mL/min
Hyp outlet Q: 47mL/min

Case O42

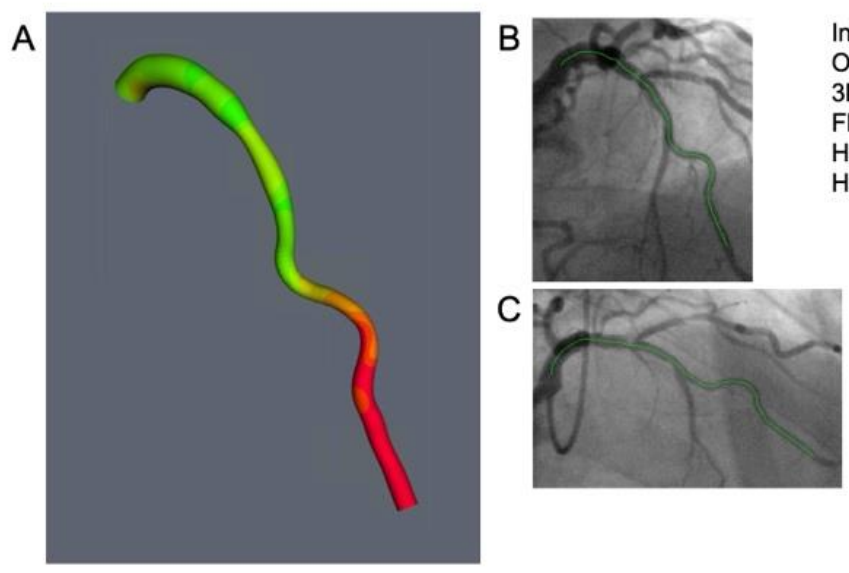

Inlet diameter: 3.71mm
Outlet diameter: 1.99mm
3D QCA: 29%
FFR: 34/58 mmHg
Hyp inlet Q: 358mL/min
Hyp outlet Q: 85mL/min

Case O45

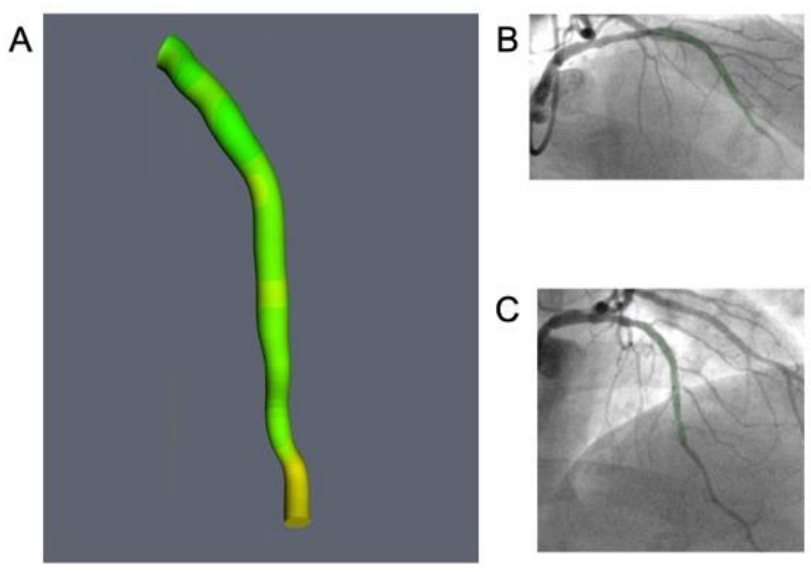

Inlet diameter: 2.85mm
Outlet diameter: 1.97mm
3D QCA: 28%
FFR: 73/88 mmHg
Hyp inlet Q: 277mL/min
Hyp outlet Q: 117mL/min

Case O49

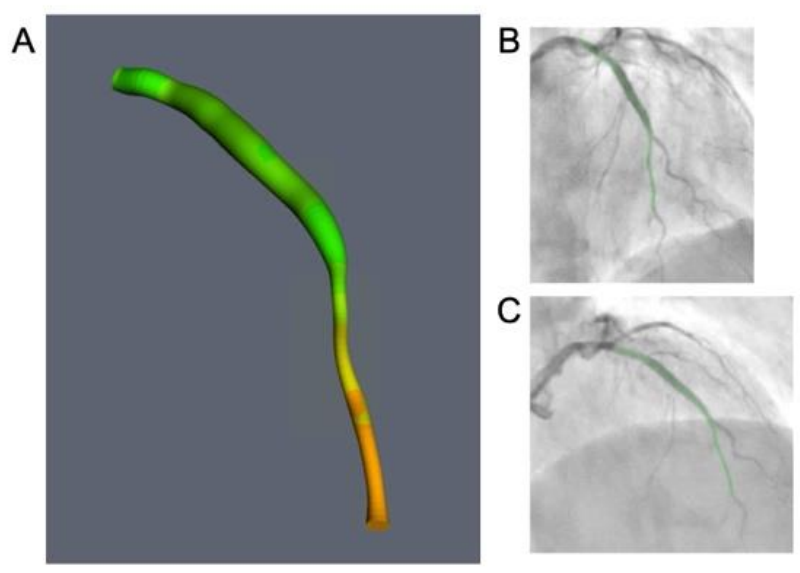

Inlet diameter: 2.15mm
Outlet diameter: 1.51mm
3D QCA: 37%
FFR: 53/70 mmHg
Hyp inlet Q: 95mL/min
Hyp outlet Q: 36mL/min

Case O51

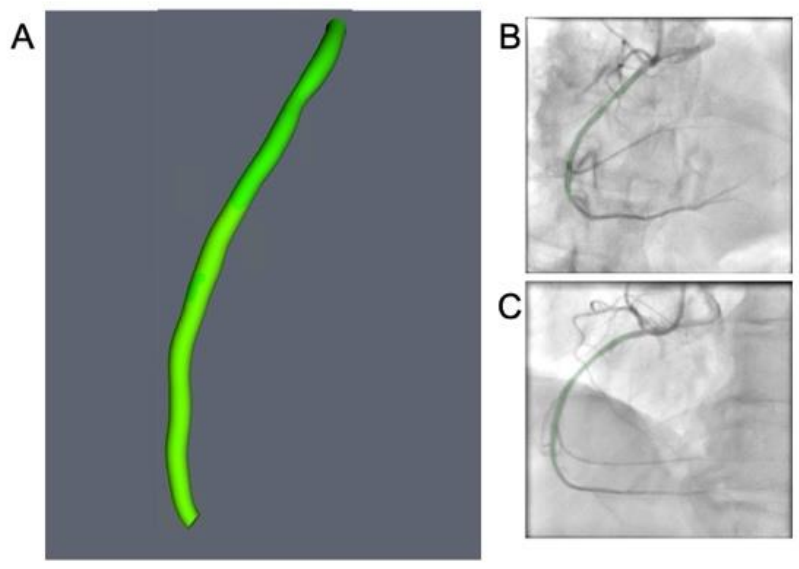

Inlet diameter: 2.25mm
Outlet diameter: 1.79mm
3D QCA: 20%
FFR: 90/96 mmHg
Hyp inlet Q: 107mL/min
Hyp outlet Q: 66mL/min

Case O52

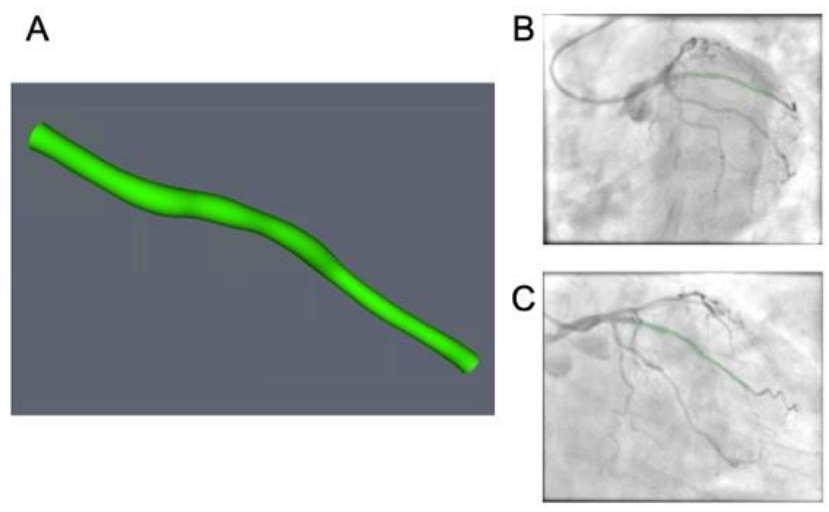

Inlet diameter: 2mm
Outlet diameter: 2mm
3D QCA: 0%
FFR: 70/73 mmHg
Hyp inlet Q: 46mL/min
Hyp outlet Q: 31mL/min

Case O53

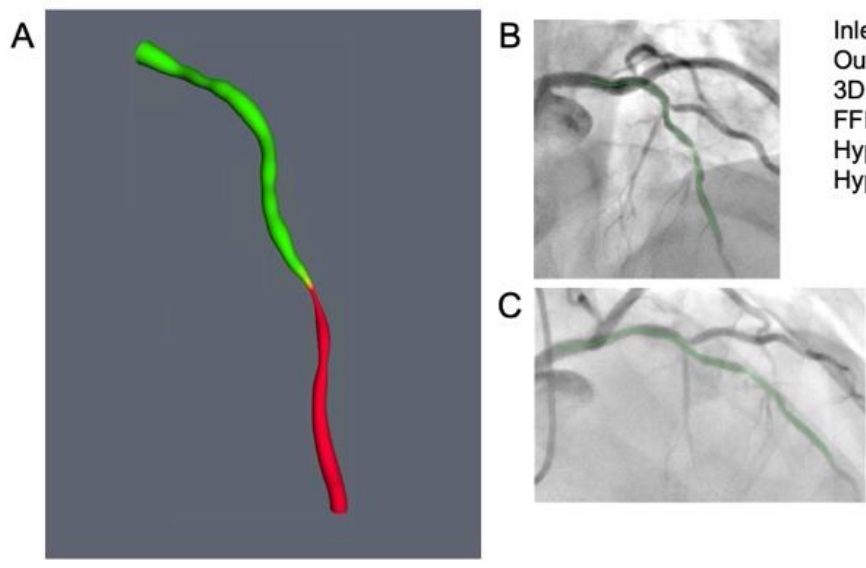

Inlet diameter: 3.76mm
Outlet diameter: 2.23mm
3D QCA: 71%
FFR: 44/104 mmHg
Hyp inlet Q: 107mL/min
Hyp outlet Q: 33mL/min

Case O54

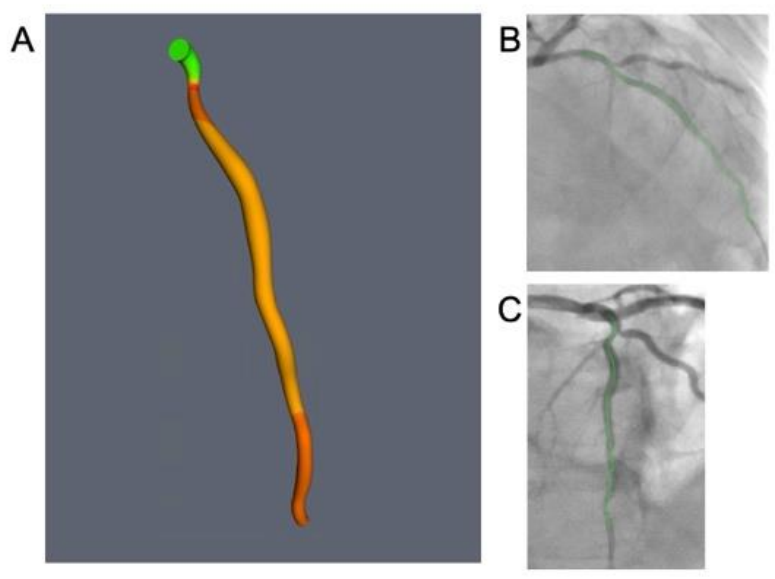

Inlet diameter: 3.15mm
Outlet diameter: 1.93mm
3D QCA: 51%
FFR: 79/109 mmHg
Hyp inlet Q: 239mL/min
Hyp outlet Q: 78mL/min

Case O55

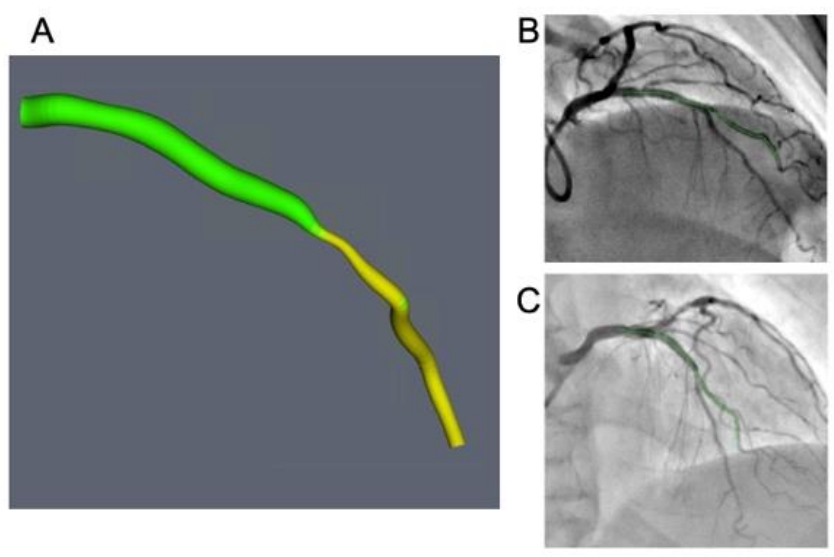

Inlet diameter: 2.67mm
Outlet diameter: 1.5mm
3D QCA: 47%
FFR: 93/116 mmHg
Hyp inlet Q: 133mL/min
Hyp outlet Q: 34mL/min

Case O56

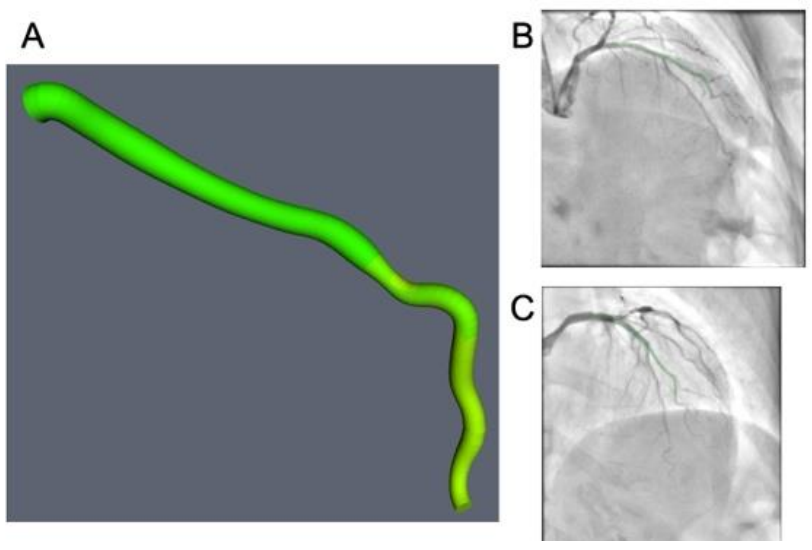

Inlet diameter: 3.05mm
Outlet diameter: 1.51mm
3D QCA: 21%
FFR: 97/113 mmHg
Hyp inlet Q: 269mL/min
Hyp outlet Q: 52mL/min

Case O57

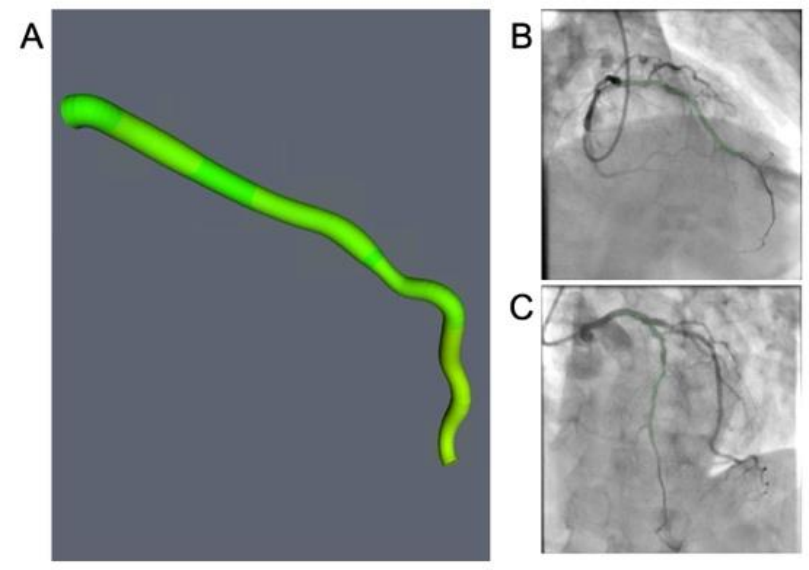

Inlet diameter: 2.79mm
Outlet diameter: 1.86mm
3D QCA: 28%
FFR: 46/67 mmHg
Hyp inlet Q: 207mL/min
Hyp outlet Q: 86mL/min

Case O58

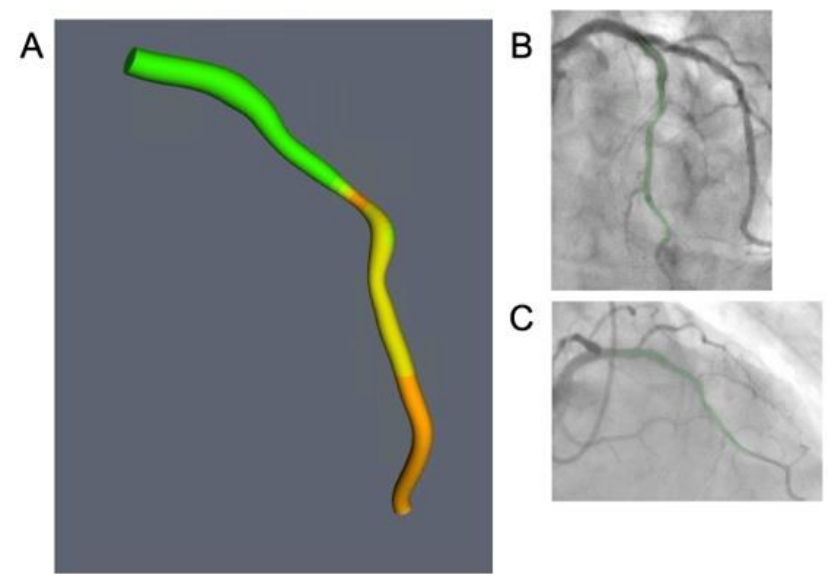

Inlet diameter: 2.56mm
Outlet diameter: 1.75mm
3D QCA: 39%
FFR: 66/86 mmHg
Hyp inlet Q: 157mL/min
Hyp outlet Q: 63mL/min

Case O61

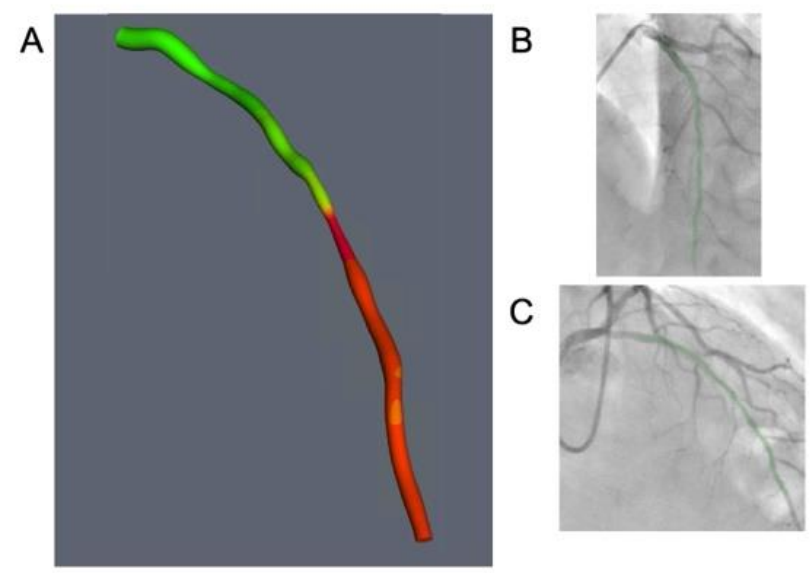

Inlet diameter: 2.78mm
Outlet diameter: 1.64mm
3D QCA: 47%
FFR: 56/95 mmHg
Hyp inlet Q: 236mL/min
Hyp outlet Q: 67mL/min

Case O62

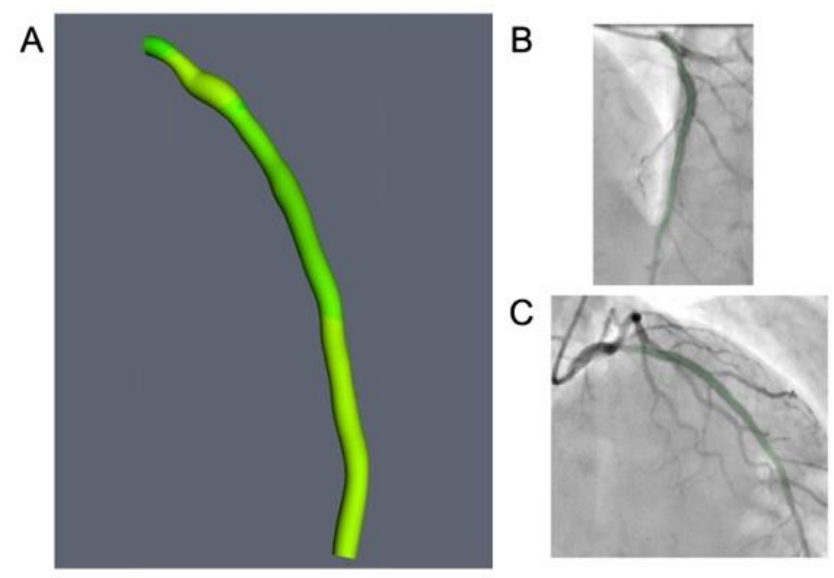

Inlet diameter: 2.55mm
Outlet diameter: 2.53mm
3D QCA: 0%
FFR: 70/81 mmHg
Hyp inlet Q: 214mL/min
Hyp outlet Q: 213mL/min

Case O63

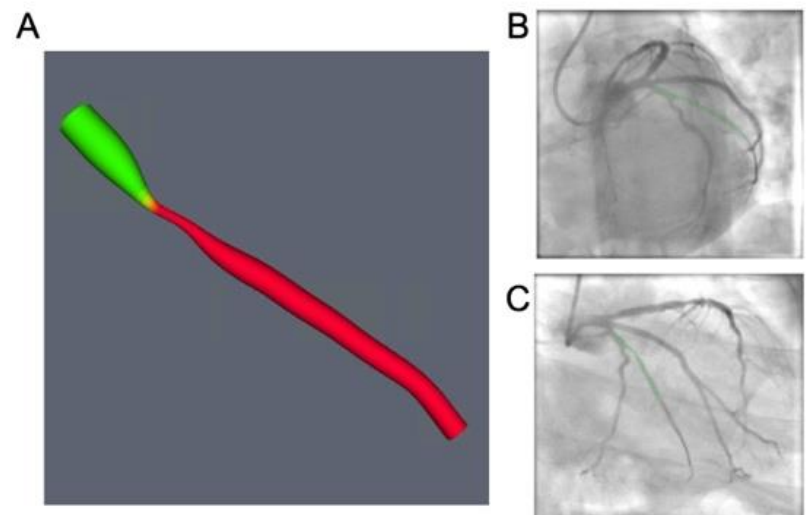

Inlet diameter: 2.65mm
Outlet diameter: 1.6mm
3D QCA: 63%
FFR: 38/86 mmHg
Hyp inlet Q: 83mL/min
Hyp outlet Q: 25mL/min

Case O64

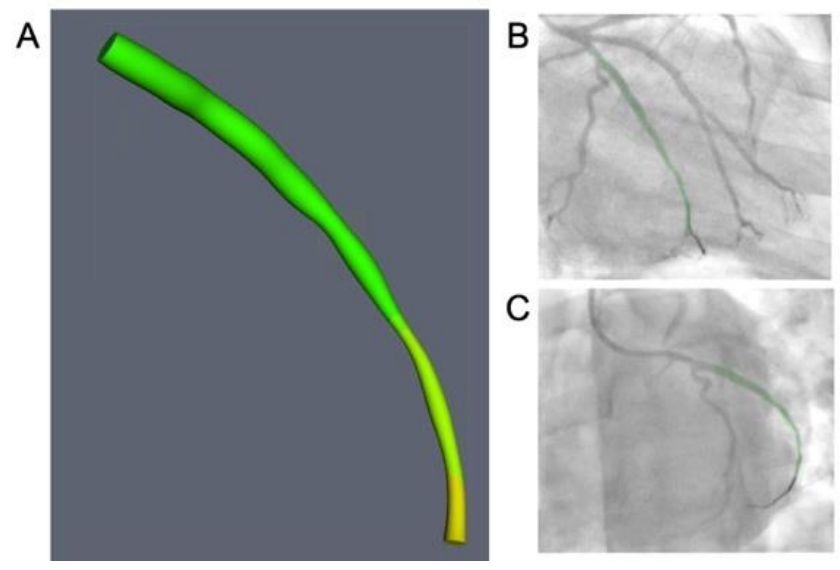

Inlet diameter: 2.64mm
Outlet diameter: 1.76mm
3D QCA: 42%
FFR: 76/90 mmHg
Hyp inlet Q: 146mL/min
Hyp outlet Q: 51mL/min

Case O65

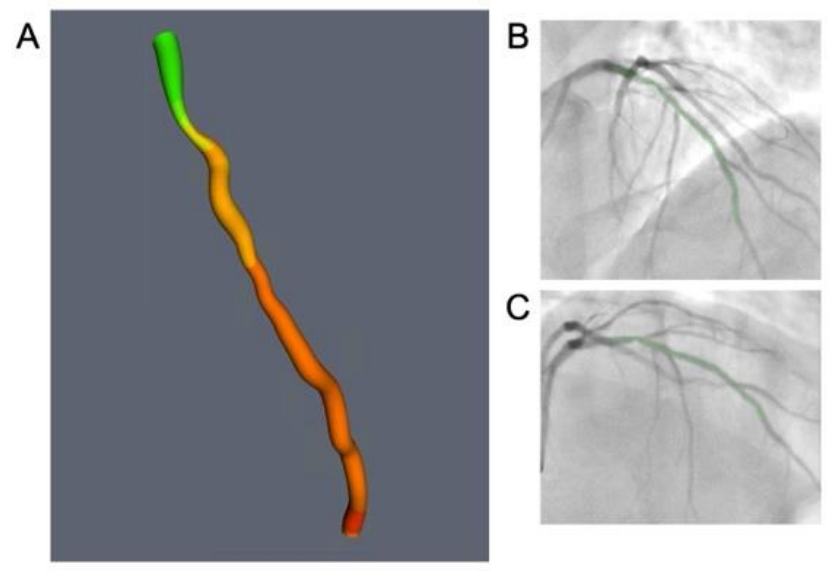

Inlet diameter: 2.78mm
Outlet diameter: 2.08mm
3D QCA: 52%
FFR: 58/83 mmHg
Hyp inlet Q: 116mL/min
Hyp outlet Q: 63mL/min

Case O66

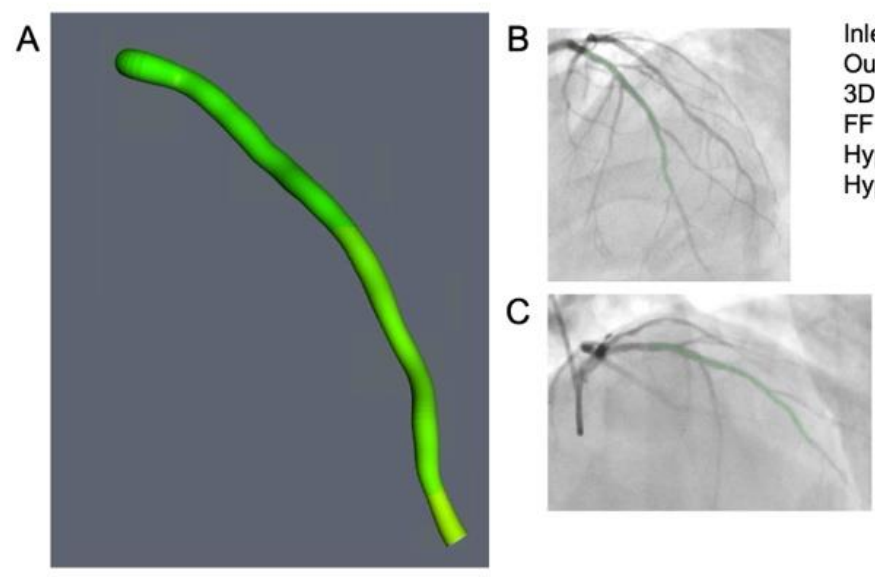

Inlet diameter: 2.89mm
Outlet diameter: 2.19mm
3D QCA: 21%
FFR: 93/104 mmHg
Hyp inlet Q: 210mL/min
Hyp outlet Q: 105mL/min

Case O67

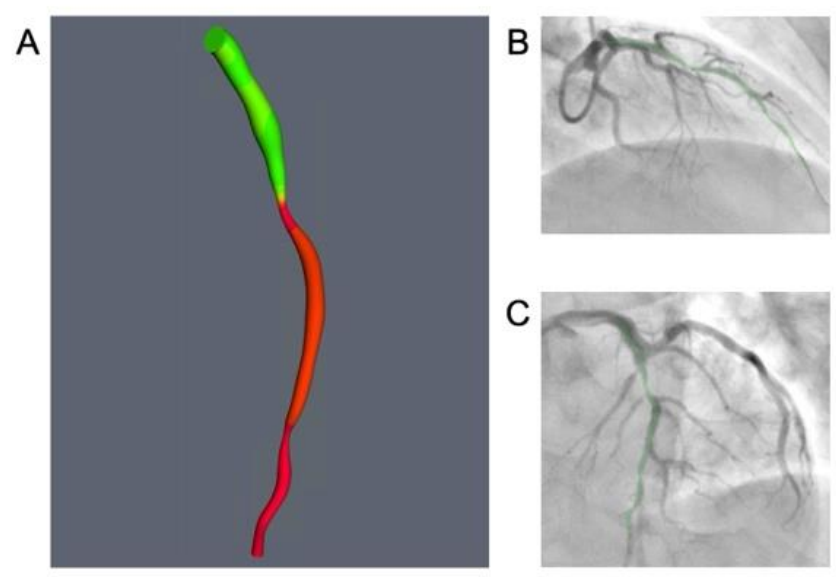

Inlet diameter: 3.34mm
Outlet diameter: 1.58mm
3D QCA: 62%
FFR: 38/80 mmHg
Hyp inlet Q: 175mL/min
Hyp outlet Q: 30mL/min

Case O68

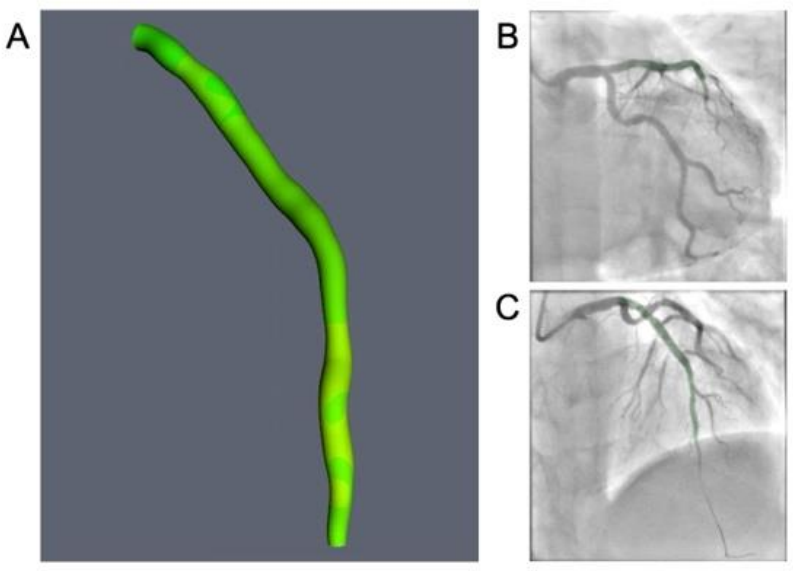

Inlet diameter: 3.28mm
Outlet diameter: 1.7mm
3D QCA: 26%
FFR: 28/31 mmHg
Hyp inlet Q: 214mL/min
Hyp outlet Q: 50mL/min

Case O69

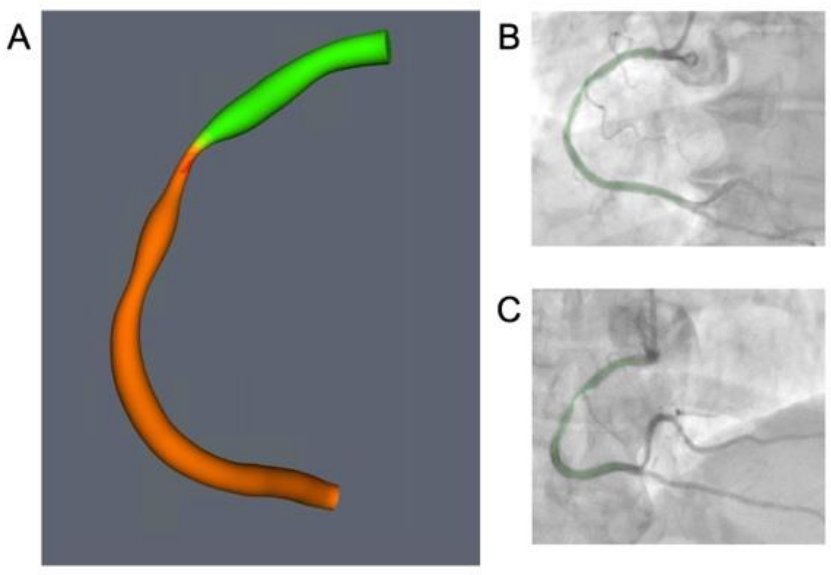

Inlet diameter: 3.08mm
Outlet diameter: 2.63mm
3D QCA: 63%
FFR: 49/70 mmHg
Hyp inlet Q: 104mL/min
Hyp outlet Q: 75mL/min

Case O70

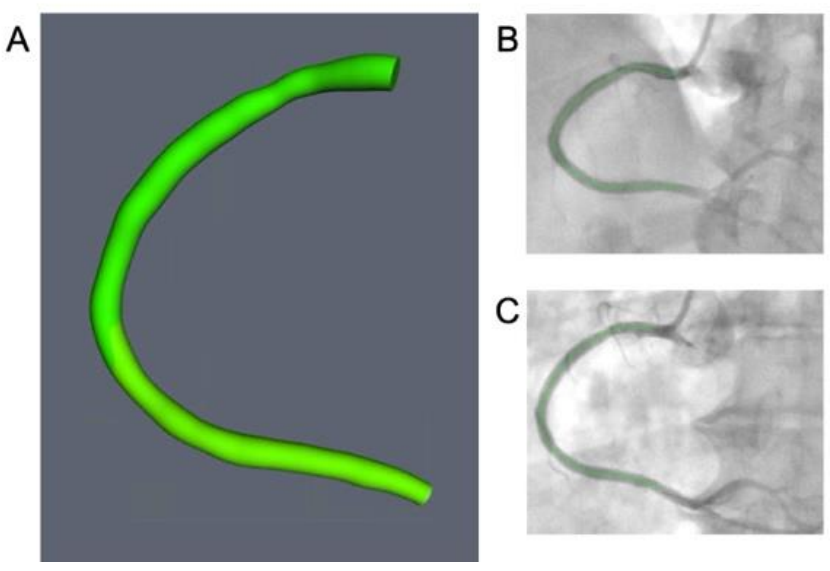

Inlet diameter: 3.27mm
Outlet diameter: 2.47mm
3D QCA: 18%
FFR: 63/68 mmHg
Hyp inlet Q: 205mL/min
Hyp outlet Q: 105mL/min

Case O71

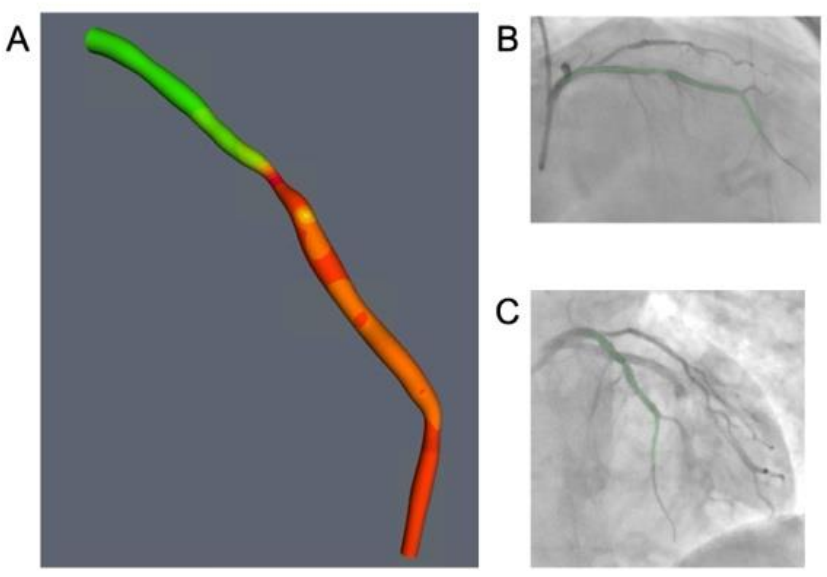

Inlet diameter: 2.92mm
Outlet diameter: 1.67mm
3D QCA: 48%
FFR: 62/97 mmHg
Hyp inlet Q: 301mL/min
Hyp outlet Q: 85mL/min

Case O74

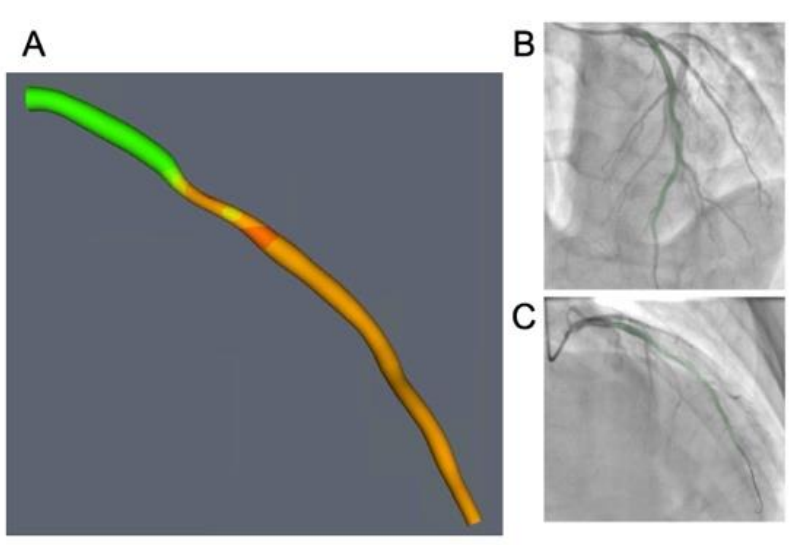

Inlet diameter: 3.55mm
Outlet diameter: 2.42mm
3D QCA: 25%
FFR: 67/83 mmHg
Hyp inlet Q: 583mL/min
Hyp outlet Q: 237mL/min

Case O75

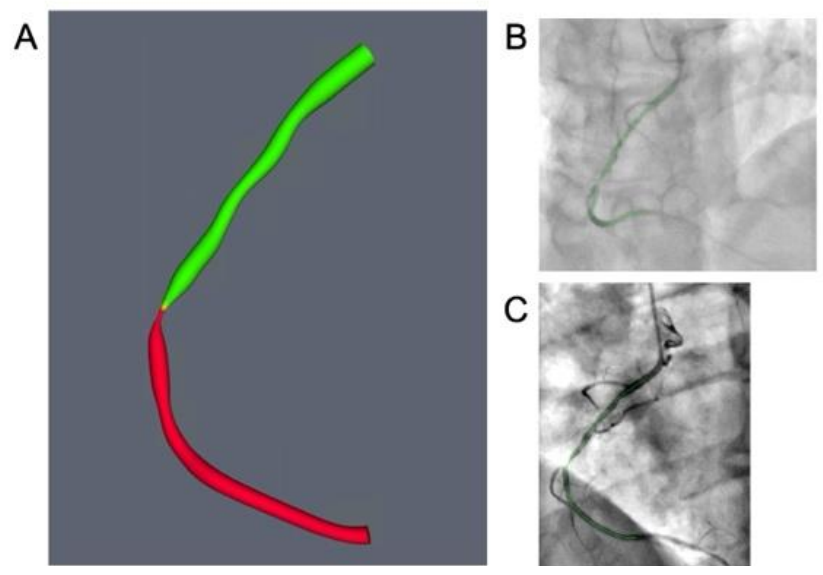

Inlet diameter: 2.86mm
Outlet diameter: 2.03mm
3D QCA: 77%
FFR: 40/99 mmHg
Hyp inlet Q: 63mL/min
Hyp outlet Q: 28mL/min

Case O

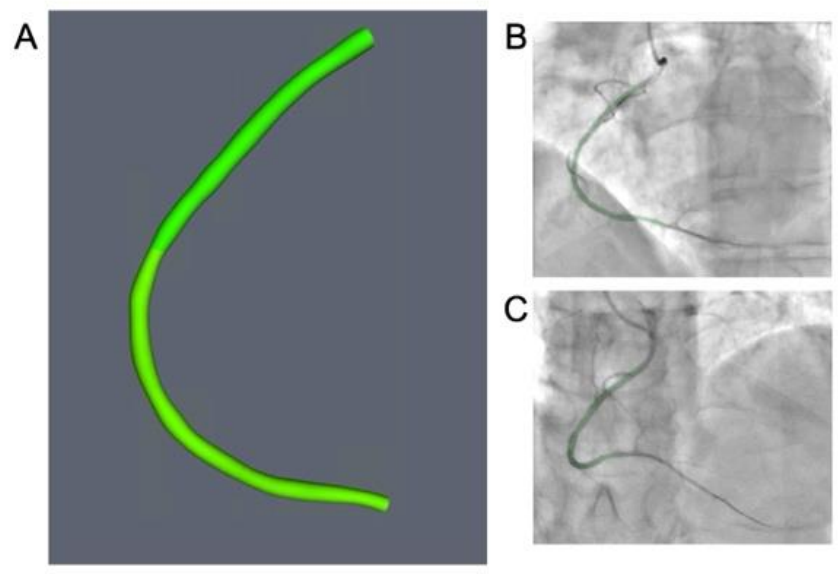

Inlet diameter: 2.53mm
Outlet diameter: 2.14mm
3D QCA: 23%
FFR: 96/106 mmHg
Hyp inlet Q: 156mL/min
Hyp outlet Q: 101mL/min

Case O78

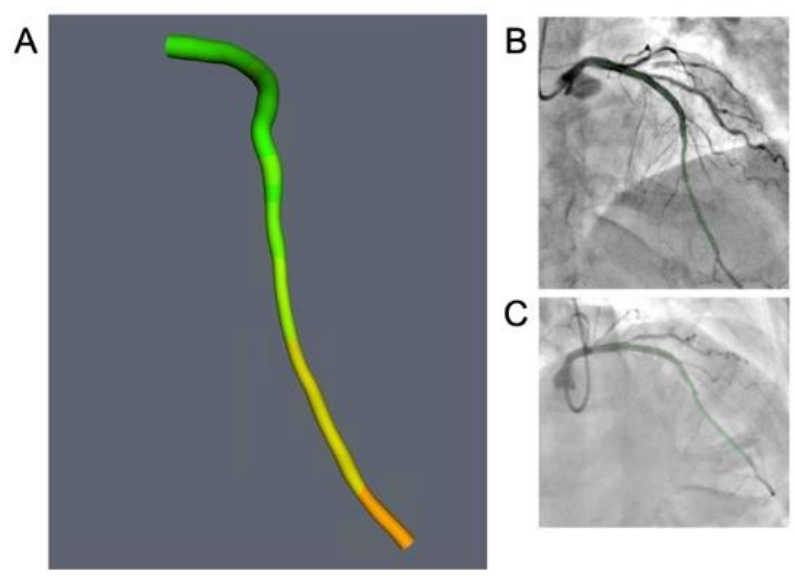

Inlet diameter: 3.39mm
Outlet diameter: 2.15mm
3D QCA: 0%
FFR: 89/114 mmHg
Hyp inlet Q: 399mL/min
Hyp outlet Q: 132mL/min

Case O79

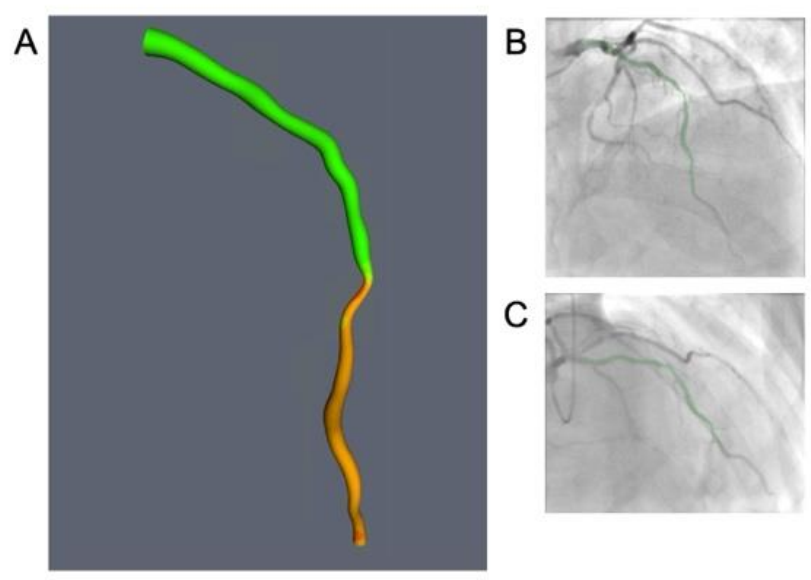

Inlet diameter: 3.27mm
Outlet diameter: 1.79mm
3D QCA: 51%
FFR: 84/112 mmHg
Hyp inlet Q: 152mL/min
Hyp outlet Q: 36mL/min

Case O81

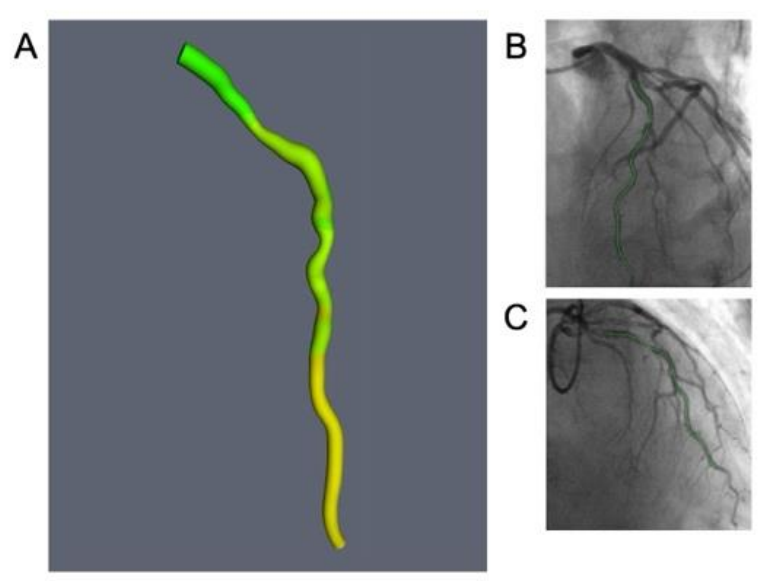

Inlet diameter: 2.82mm
Outlet diameter: 1.68mm
3D QCA: 42%
FFR: 76/92 mmHg
Hyp inlet Q: 163mL/min
Hyp outlet Q: 49mL/min

Case O82

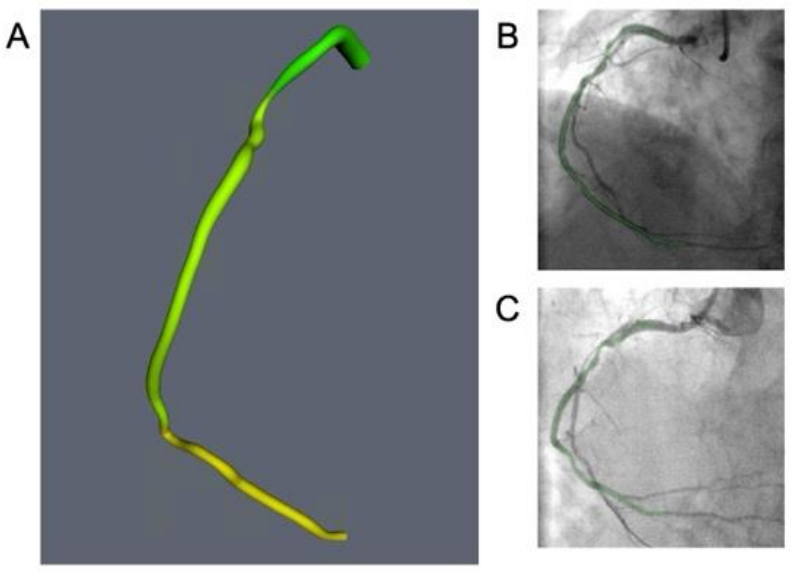

Inlet diameter: 2.66mm
Outlet diameter: 1.22mm
3D QCA: 63%
FFR: 64/79 mmHg
Hyp inlet Q: 47mL/min
Hyp outlet Q: 8mL/min

Case O84

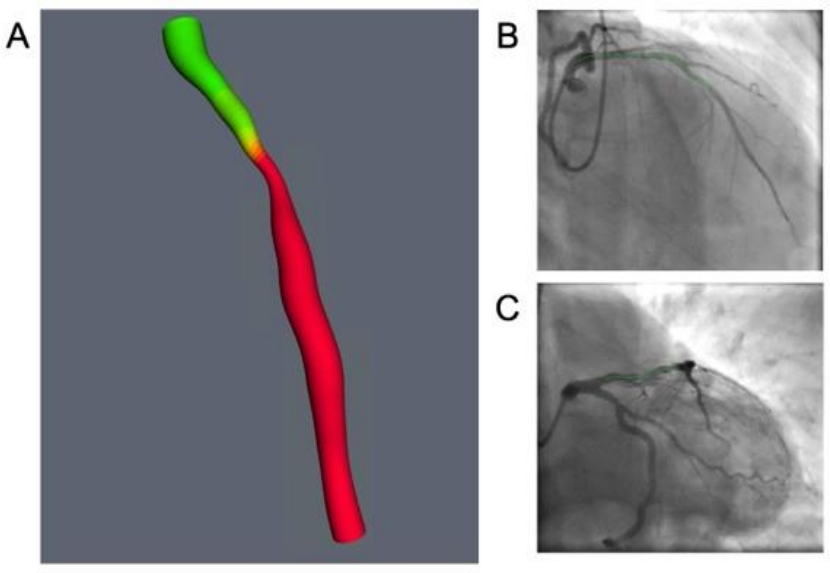

Inlet diameter: 3.07mm
Outlet diameter: 2.34mm
3D QCA: 60%
FFR: 46/86 mmHg
Hyp inlet Q: 177mL/min
Hyp outlet Q: 91mL/min

Case O85

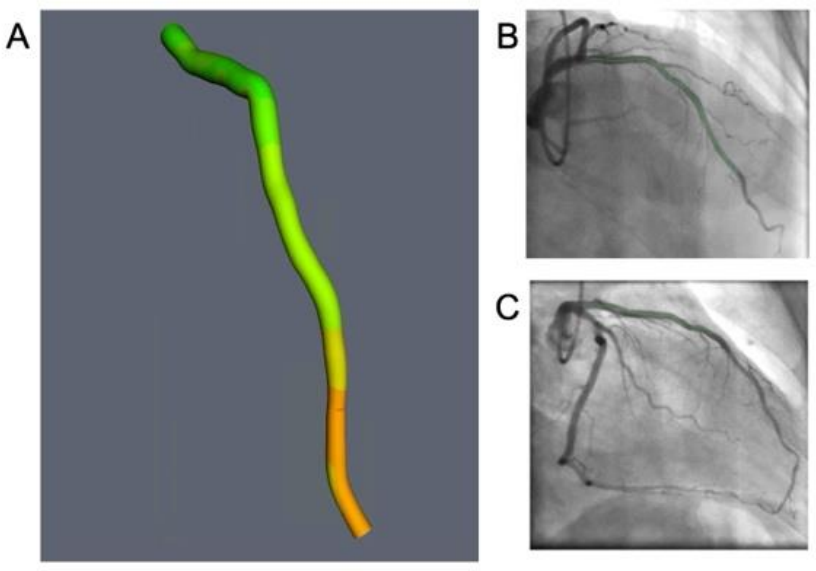

Inlet diameter: 3.22mm
Outlet diameter: 2.49mm
3D QCA: 0%
FFR: 82/108 mmHg
Hyp inlet Q: 563mL/min
Hyp outlet Q: 305mL/min

Case O89

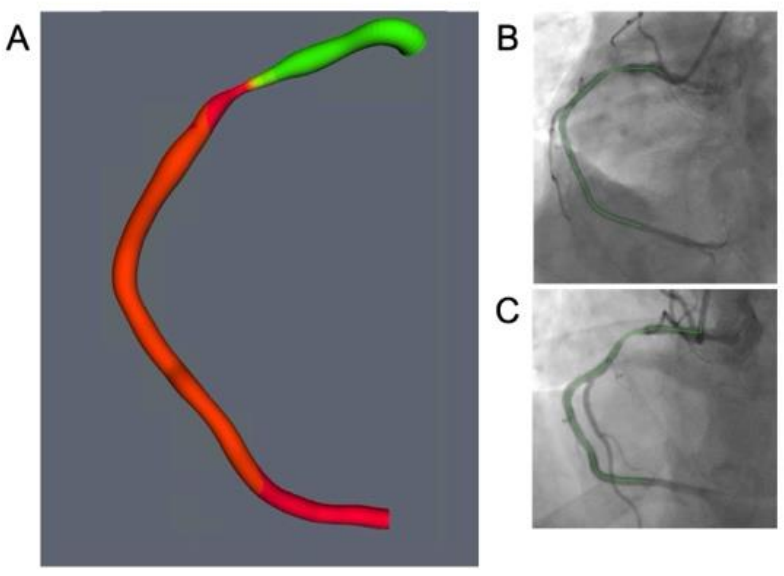

Inlet diameter: 3.22mm
Outlet diameter: 2.29mm
3D QCA: 62%
FFR: 52/82 mmHg
Hyp inlet Q: 128mL/min
Hyp outlet Q: 60mL/min

Case O90

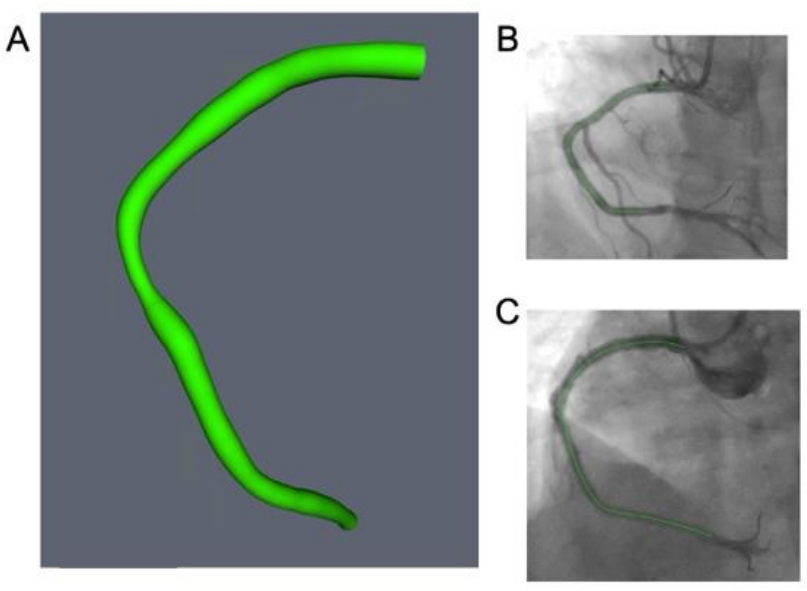

Inlet diameter: 3.34mm
Outlet diameter: 2.12mm
3D QCA: 41%
FFR: 64/65 mmHg
Hyp inlet Q: 37mL/min
Hyp outlet Q: 12mL/min

Case O91

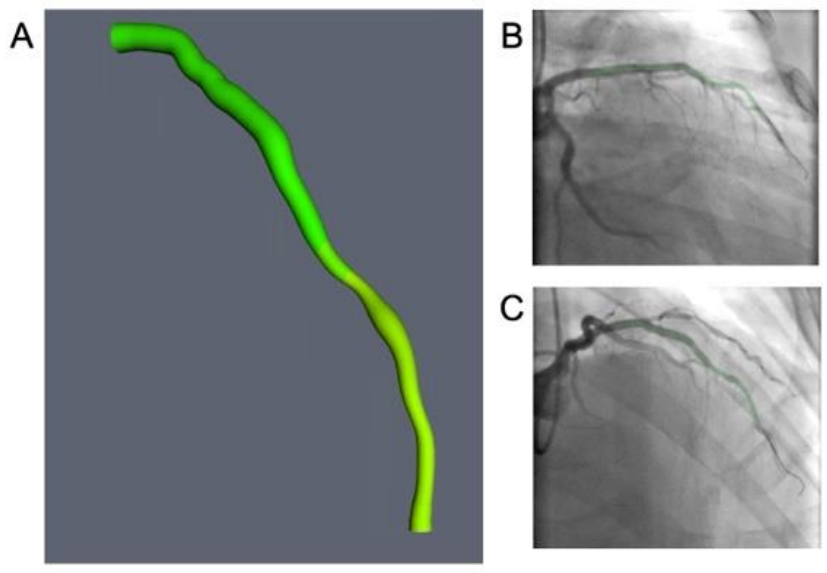

Inlet diameter: 3.35mm
Outlet diameter: 2.1mm
3D QCA: 40%
FFR: 83/97 mmHg
Hyp inlet Q: 236mL/min
Hyp outlet Q: 74mL/min

Case O93

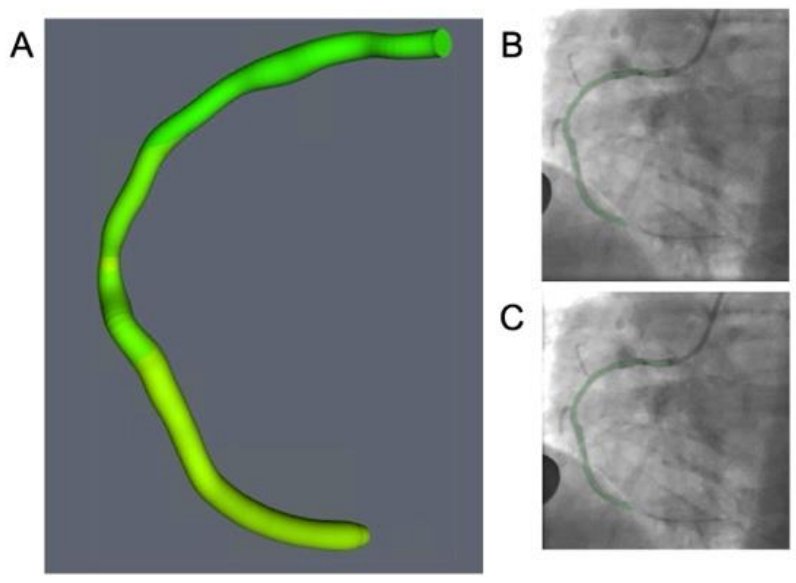

Inlet diameter: 2.25mm
Outlet diameter: 1.82mm
3D QCA: 37%
FFR: 71/81 mmHg
Hyp inlet Q: 109mL/min
Hyp outlet Q: 82mL/min

Case O94

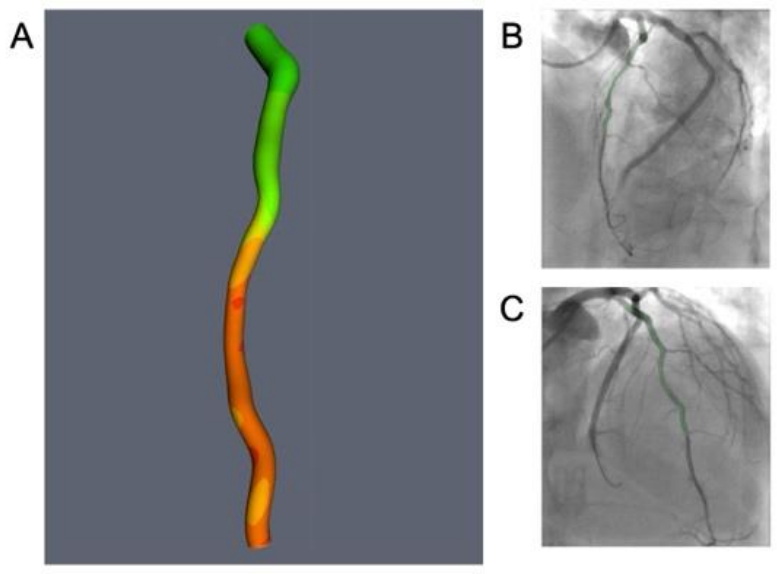

Inlet diameter: 3.22mm
Outlet diameter: 2.15mm
3D QCA: 0%
FFR: 57/81 mmHg
Hyp inlet Q: 496mL/min
Hyp outlet Q: 190mL/min

Case O95

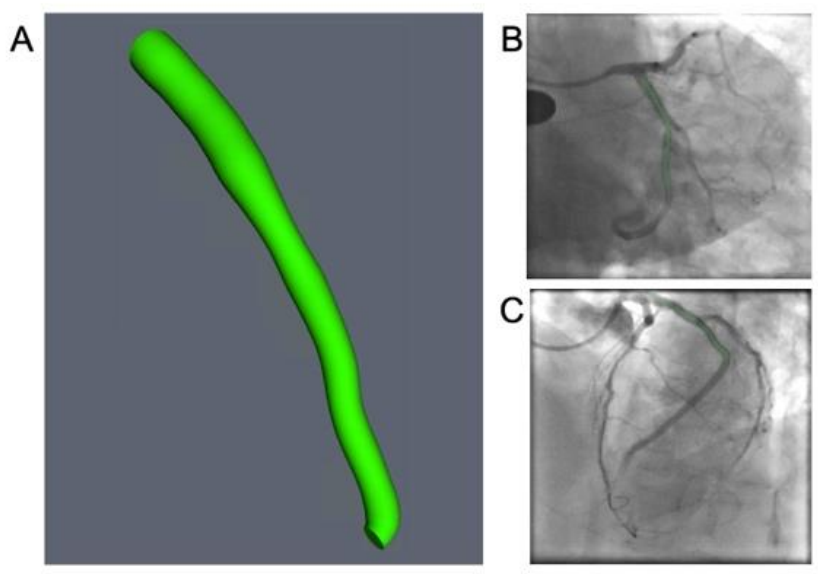

Inlet diameter: 3.97mm
Outlet diameter: 2.18mm
3D QCA: 0%
FFR: 68/68 mmHg
Hyp inlet Q: 124mL/min
Hyp outlet Q: 33mL/min

Case O96

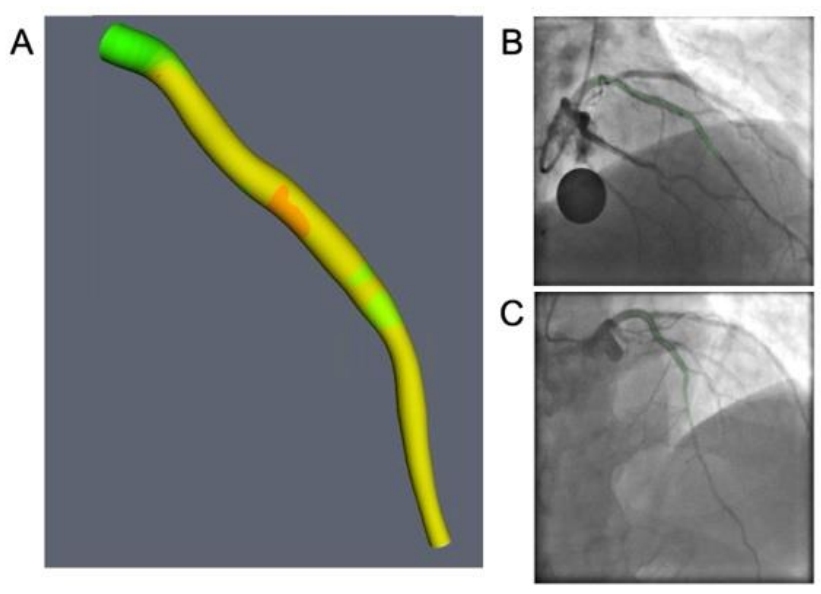

Inlet diameter: 3.37mm
Outlet diameter: 1.81mm
3D QCA: 21%
FFR: 71/89 mmHg
Hyp inlet Q: 627mL/min
Hyp outlet Q: 145mL/min

Case O97

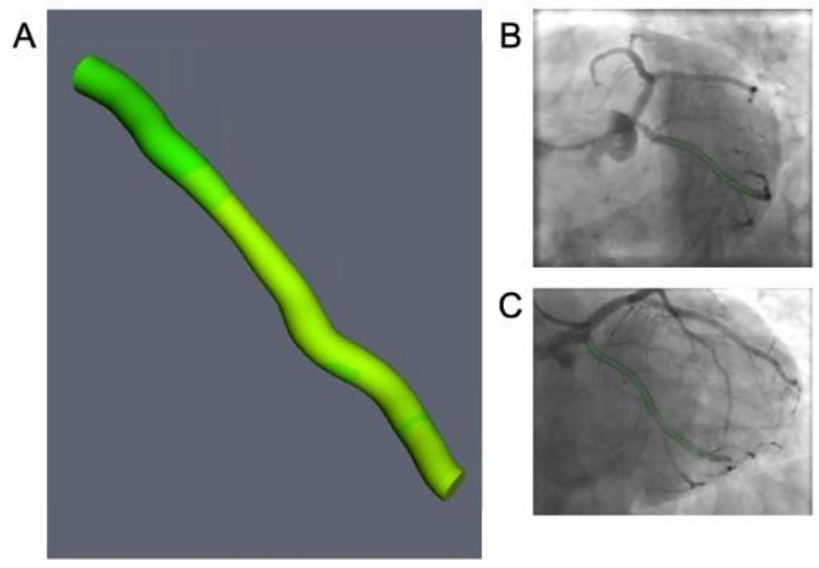

Inlet diameter: 3.24mm
Outlet diameter: 2.67mm
3D QCA: 0%
FFR: 83/97 mmHg
Hyp inlet Q: 447mL/min
Hyp outlet Q: 287mL/min

Case O98

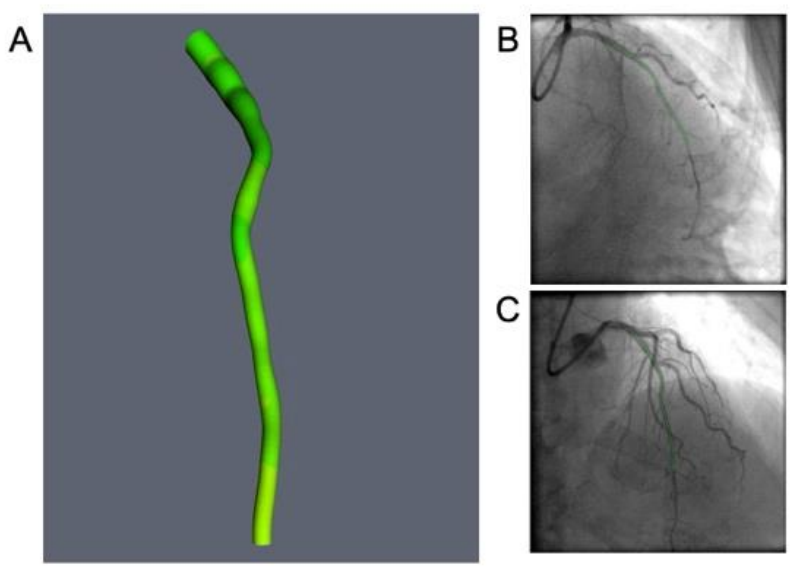

Inlet diameter: 2.82mm
Outlet diameter: 1.45mm
3D QCA: 0%
FFR: 94/107 mmHg
Hyp inlet Q: 218mL/min
Hyp outlet Q: 45mL/min

Case O100

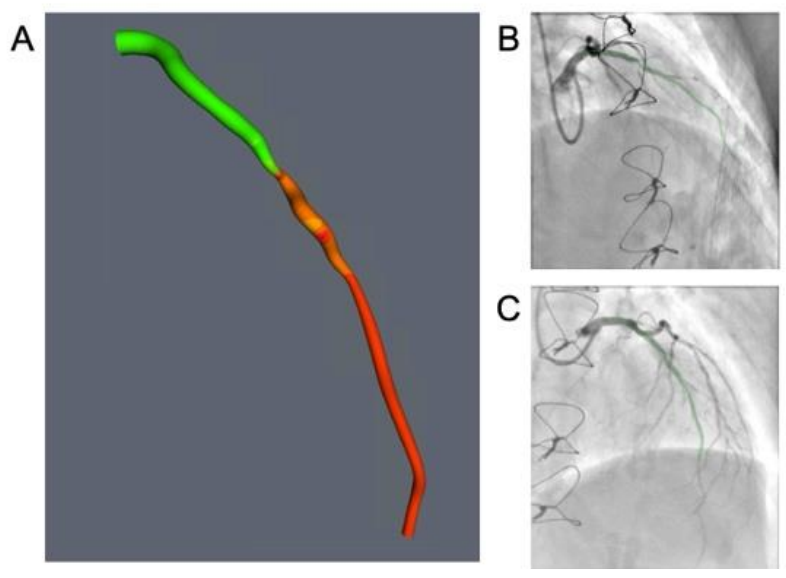

Inlet diameter: 3.58mm
Outlet diameter: 1.62mm
3D QCA: 47%
FFR: 74/112 mmHg
Hyp inlet Q: 266mL/min
Hyp outlet Q: 41mL/min

Case O103

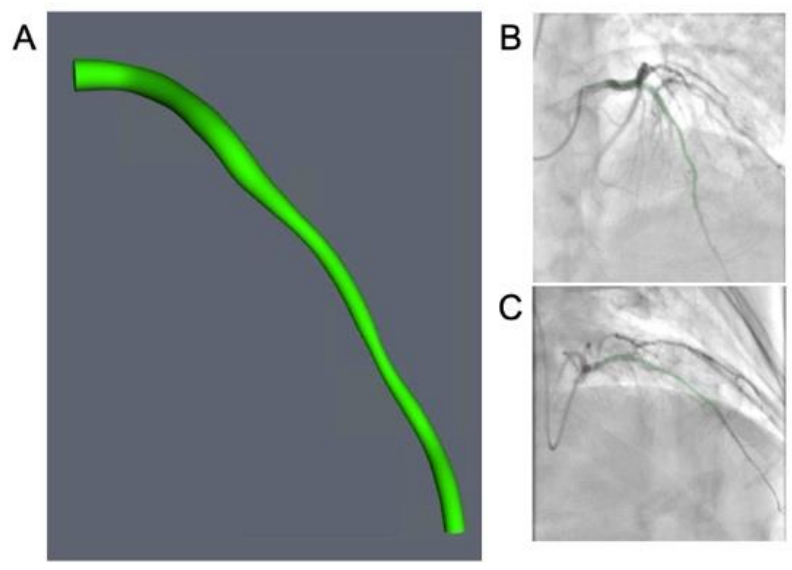

Inlet diameter: 3.56mm
Outlet diameter: 2.05mm
3D QCA: 23%
FFR: 76/80 mmHg
Hyp inlet Q: 95mL/min
Hyp outlet Q: 27mL/min

Case O104

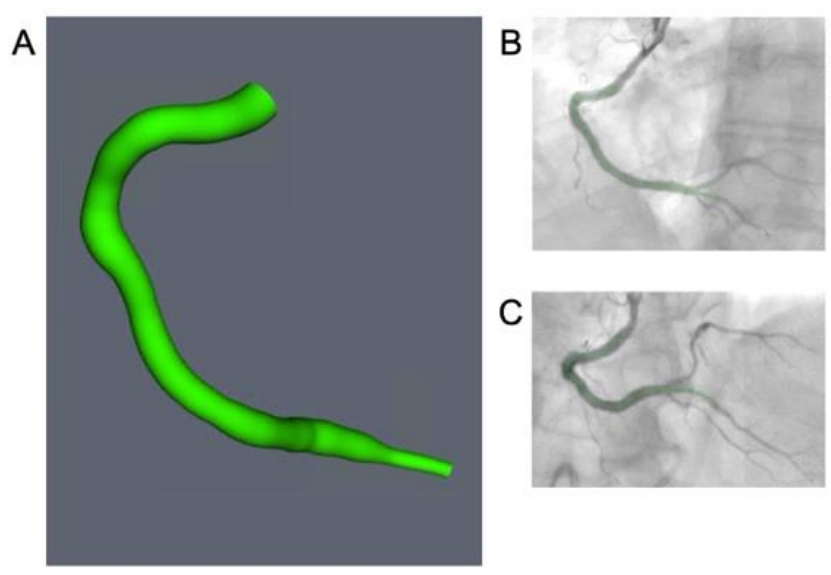

Inlet diameter: 4.5mm
Outlet diameter: 1.98mm
3D QCA: 0%
FFR: 37/38 mmHg
Hyp inlet Q: 145mL/min
Hyp outlet Q: 21mL/min

Case O107

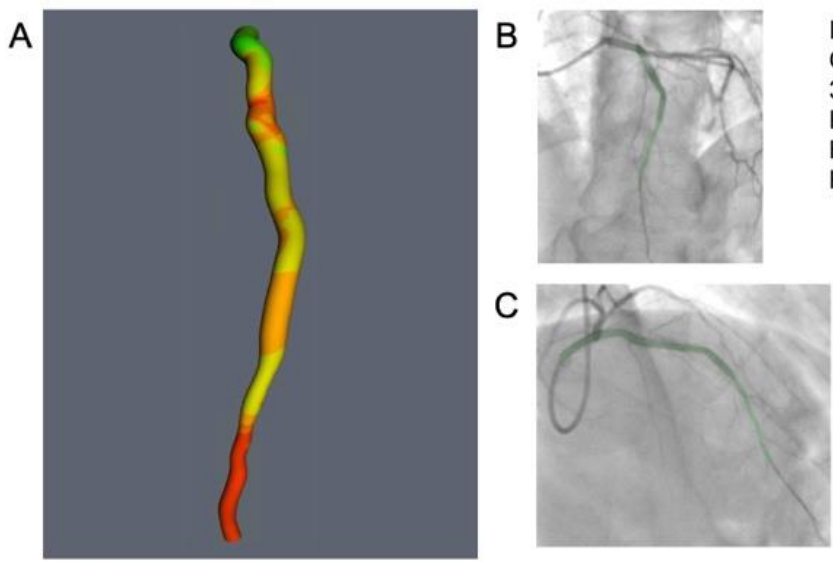

Inlet diameter: 2.81mm
Outlet diameter: 1.83mm
3D QCA: 20%
FFR: 61/92 mmHg
Hyp inlet Q: 406mL/min
Hyp outlet Q: 148mL/min

Case O108

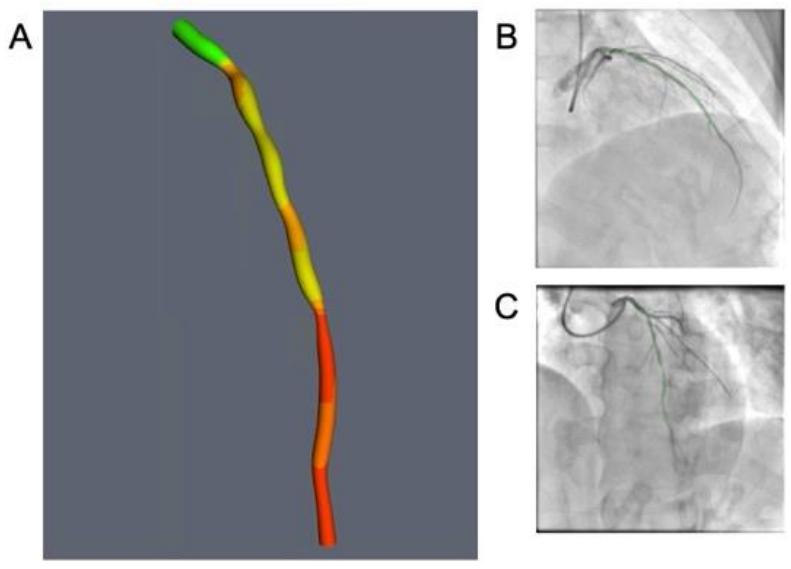

Inlet diameter: 2.59mm
Outlet diameter: 1.82mm
3D QCA: 31%
FFR: 54/80 mmHg
Hyp inlet Q: 163mL/min
Hyp outlet Q: 70mL/min

Case O109

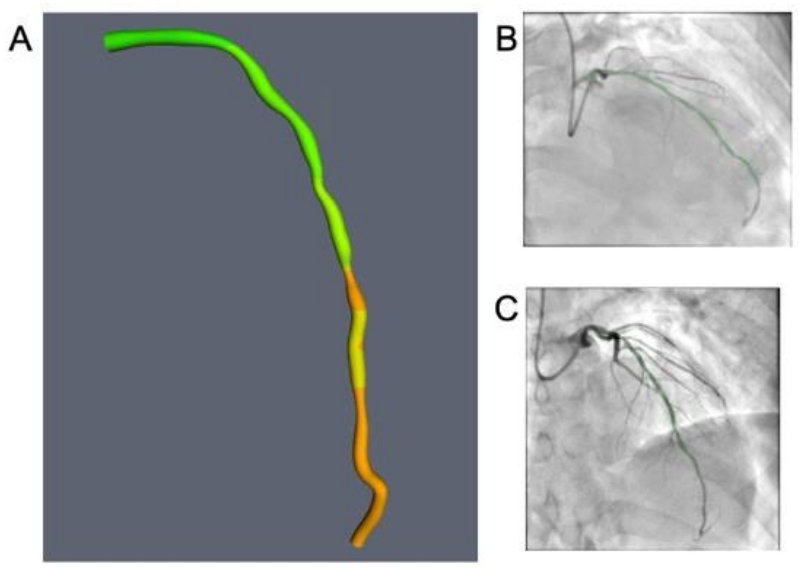

Inlet diameter: 2.77mm
Outlet diameter: 1.84mm
3D QCA: 45%
FFR: 67/88 mmHg
Hyp inlet Q: 104mL/min
Hyp outlet Q: 41mL/min

Case O110

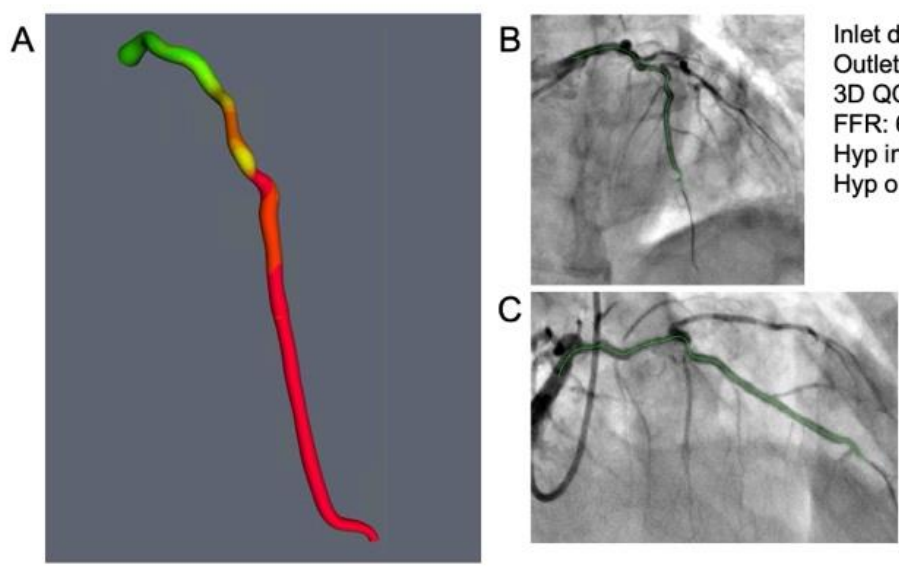

Inlet diameter: 3.81mm
Outlet diameter: 1.37mm
3D QCA: 30%
FFR: 69/117 mmHg
Hyp inlet Q: 517mL/min
Hyp outlet Q: 50mL/min

Case O111

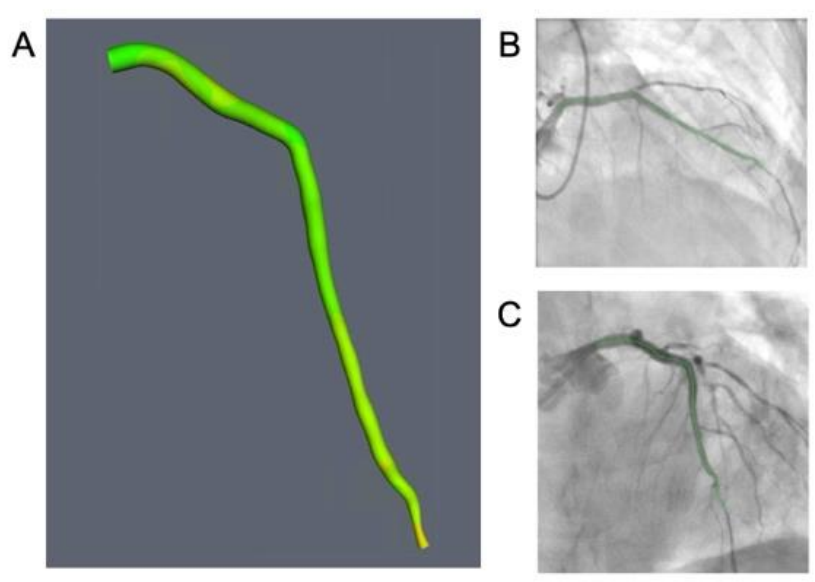

Inlet diameter: 3.8mm
Outlet diameter: 1.57mm
3D QCA: 33%
FFR: 87/107 mmHg
Hyp inlet Q: 614mL/min
Hyp outlet Q: 70mL/min

Case O112

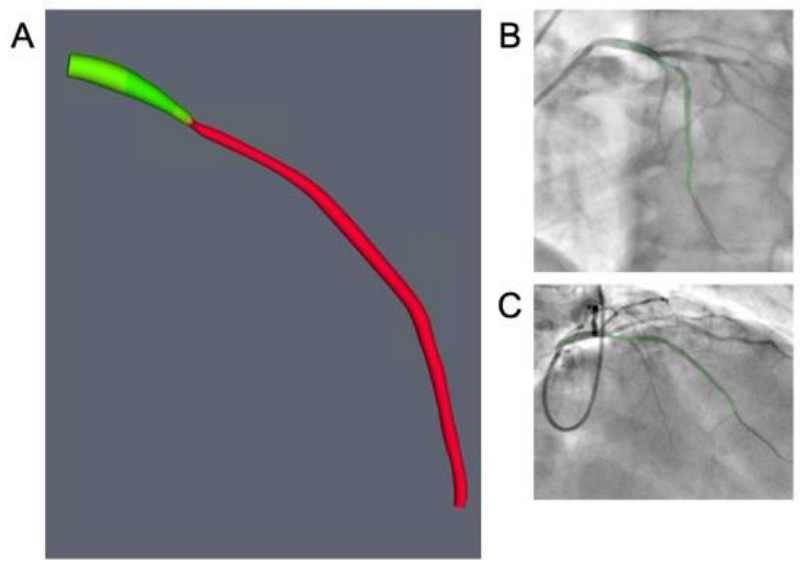

Inlet diameter: 2.89mm
Outlet diameter: 1.51mm
3D QCA: 48%
FFR: 38/106 mmHg
Hyp inlet Q: 262mL/min
Hyp outlet Q: 59mL/min

Case O113

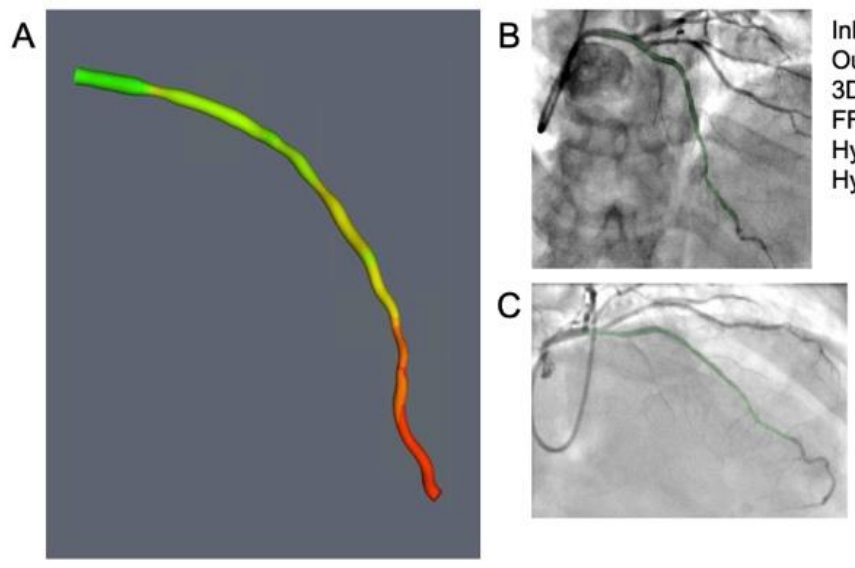

Inlet diameter: 3.23mm
Outlet diameter: 2.13mm
3D QCA: 38%
FFR: 64/97 mmHg
Hyp inlet Q: 222mL/min
Hyp outlet Q: 87mL/min

Case O114

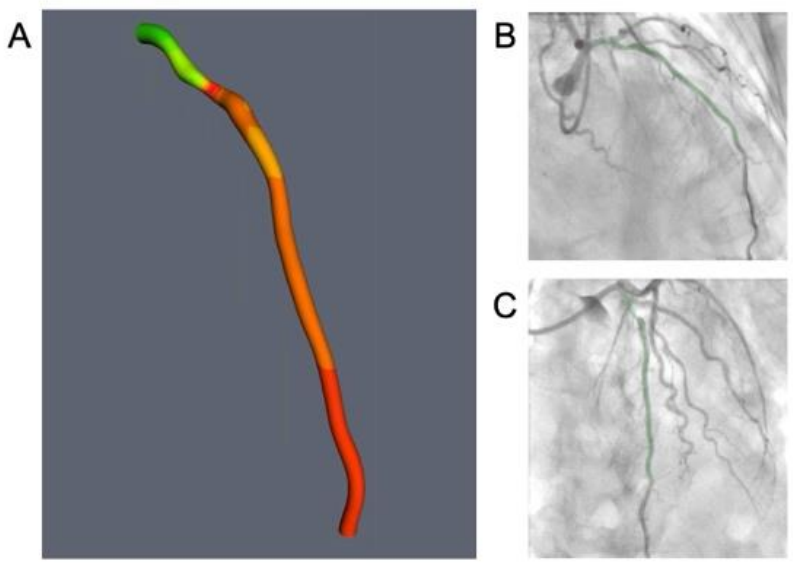

Inlet diameter: 2.74mm
Outlet diameter: 2.25mm
3D QCA: 32%
FFR: 53/81 mmHg
Hyp inlet Q: 265mL/min
Hyp outlet Q: 166mL/min

Case O115

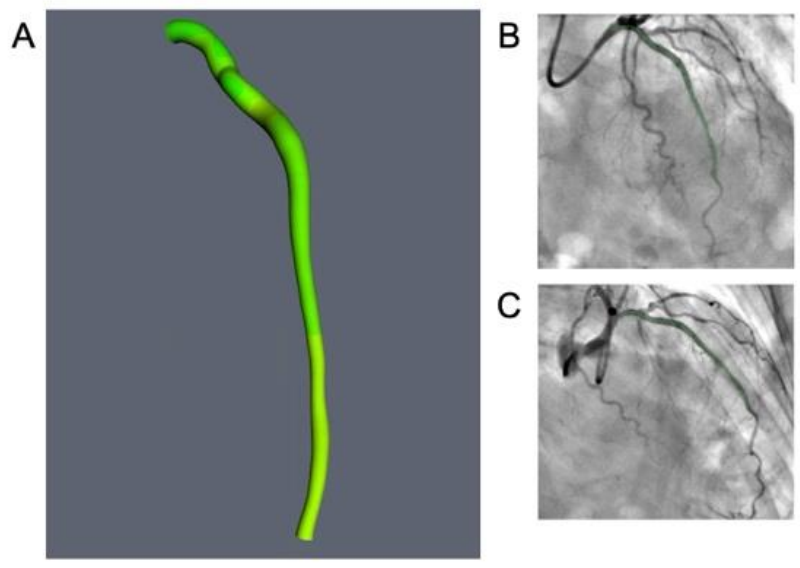

Inlet diameter: 3.13mm
Outlet diameter: 2.09mm
3D QCA: 22%
FFR: 78/91 mmHg
Hyp inlet Q: 276mL/min
Hyp outlet Q: 105mL/min

Case O118

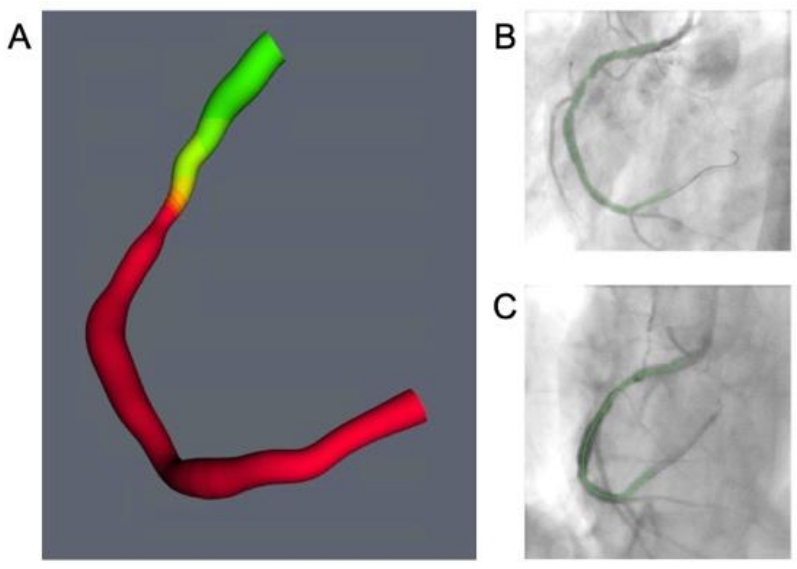

Inlet diameter: 3.22mm
Outlet diameter: 2.5mm
3D QCA: 38%
FFR: 40/78 mmHg
Hyp inlet Q: 331mL/min
Hyp outlet Q: 182mL/min

Case O119

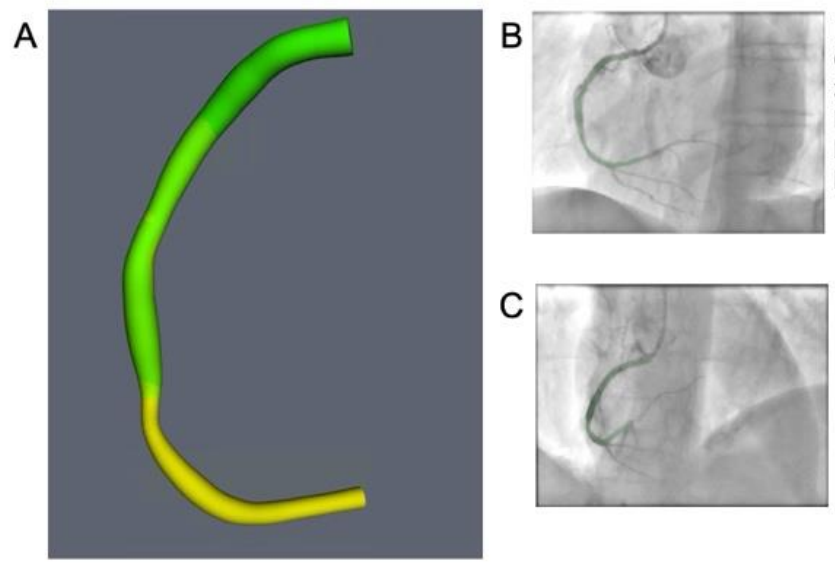

Inlet diameter: 3.26mm
Outlet diameter: 2.22mm
3D QCA: 37%
FFR: 55/67 mmHg
Hyp inlet Q: 274mL/min
Hyp outlet Q: 113mL/min

Case O120

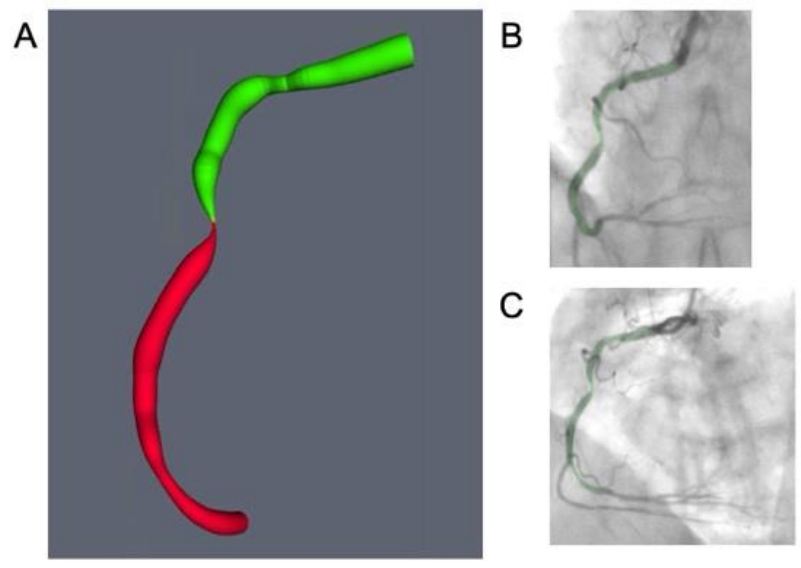

Inlet diameter: 3.4mm
Outlet diameter: 2.32mm
3D QCA: 86%
FFR: 47/83 mmHg
Hyp inlet Q: 17mL/min
Hyp outlet Q: 7mL/min

Case O122

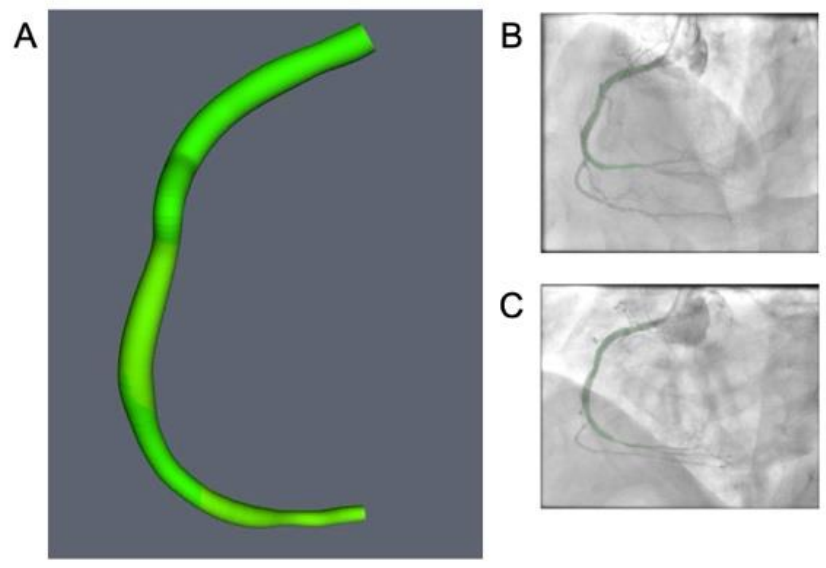

Inlet diameter: 3.03mm
Outlet diameter: 1.7mm
3D QCA: 0%
FFR: 84/89 mmHg
Hyp inlet Q: 180mL/min
Hyp outlet Q: 47mL/min

Case O123

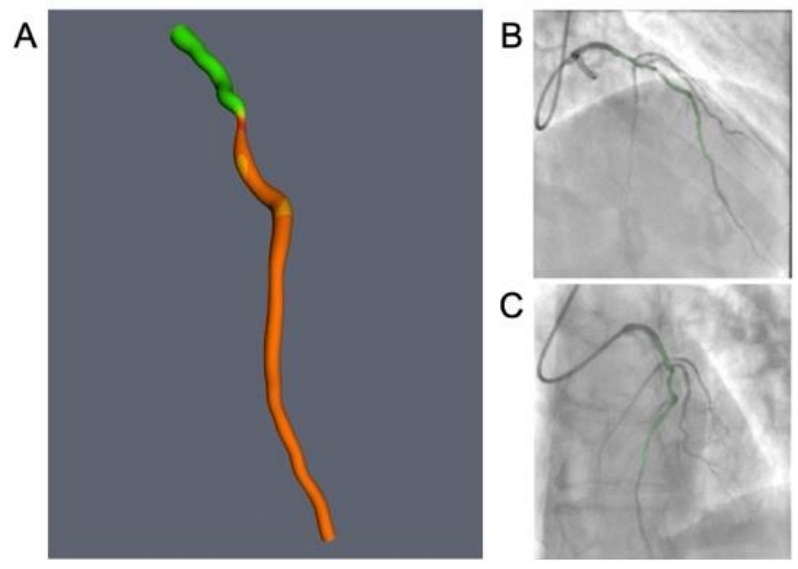

Inlet diameter: 2.91mm
Outlet diameter: 1.82mm
3D QCA: 54%
FFR: 61/89 mmHg
Hyp inlet Q: 128mL/min
Hyp outlet Q: 42mL/min

Case O124

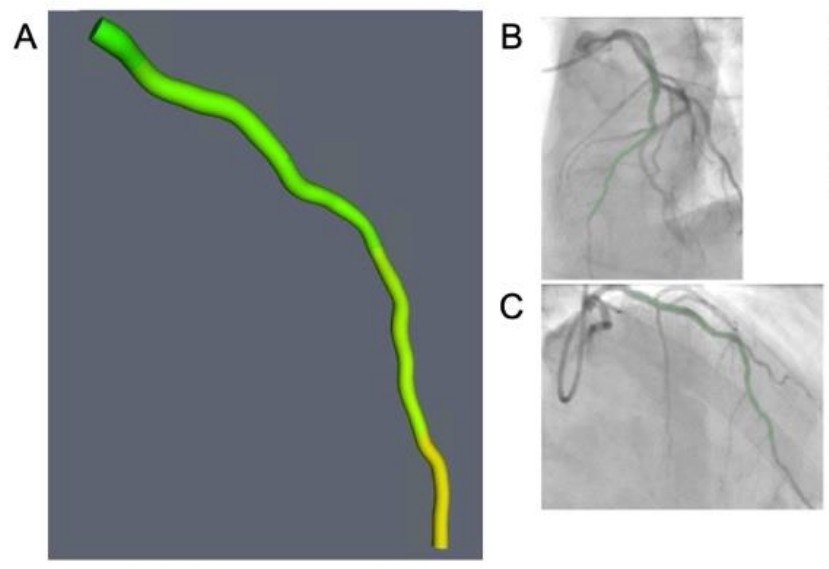

Inlet diameter: 3.14mm
Outlet diameter: 1.97mm
3D QCA: 0%
FFR: 73/90 mmHg
Hyp inlet Q: 271mL/min
Hyp outlet Q: 88mL/min

Case O125

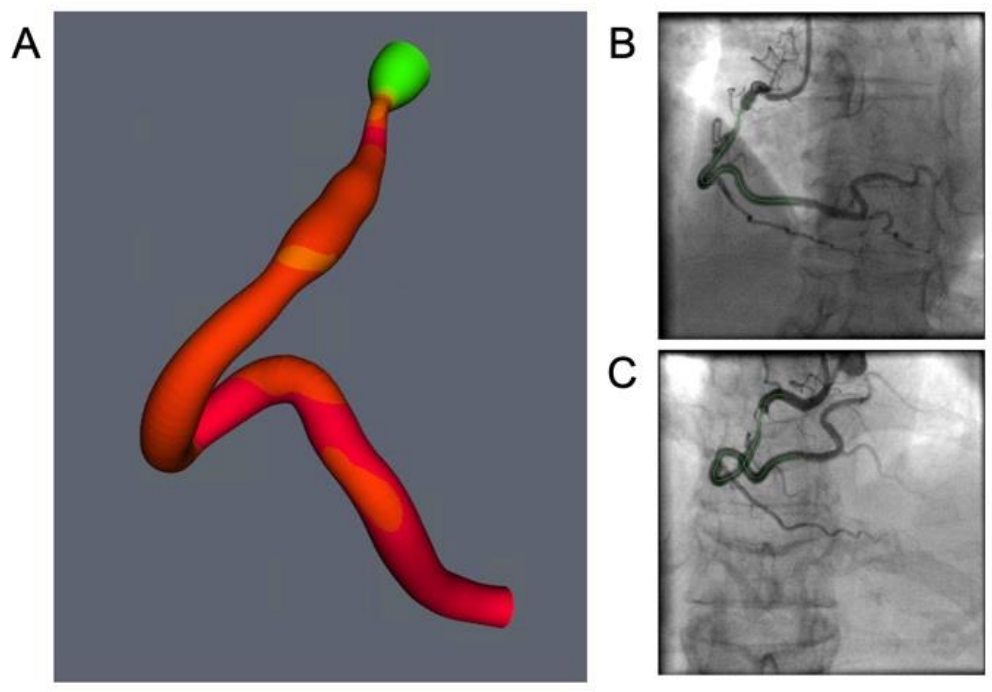

Inlet diameter: 3.69mm
Outlet diameter: 2.74mm
3D QCA: 62%
FFR: 46/74 mmHg
Hyp inlet Q: 154mL/min
Hyp outlet Q: 81mL/min

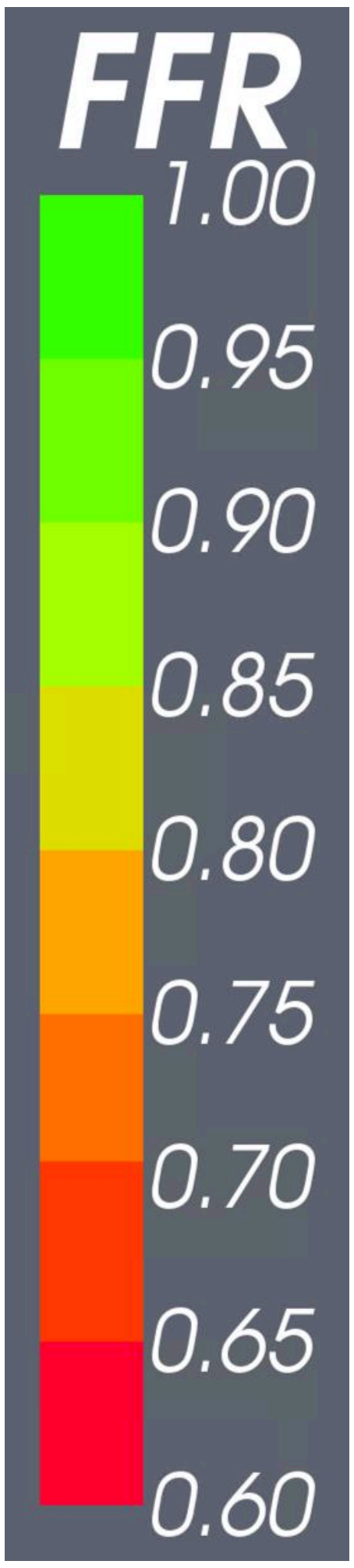

Table A: Demographics and lesion characteristics of excluded patients
BMI, body mass index; CCS, chronic coronary syndrome; NSTEMI, non-ST elevation myocardial infarction; PCI, percutaneous coronary intervention; STEMI, ST-elevation myocardial infarction.

**Demographics (n = 13)**	
Age, y	67.3 ±12.4
Male	10 (83%)
BMI	26.4 ±4.3
Current or ex-smoker	6 (50%)
**Enrolment pathology**	
CCS	5 (42%)
STEMI	2 (17%)
NSTEMI	5 (42%)
**Comorbidities**	
Left ventricular ejection fraction	51.1 ±8.5
Hypertension	4 (33%)
Diabetes mellitus	2 (17%)
Hypercholesterolaemia	4 (33%)
Previous myocardial infarction	5 (42%)
Previous stroke	0
**Vessel characteristics (n = 43)**	
Left anterior descending	18 (42%)
Left circumflex	14 (33%)
Right coronary artery	11 (25%)
Pre-PCI physiology assessment	23 (59%)

Figure D: ROC sensitivity analysis for microvascular resistance reserve (MRR)
Optimal diagnostic threshold 2.5. AUC 95% CI calculated using DeLong's method. Data derived from 116 observations.

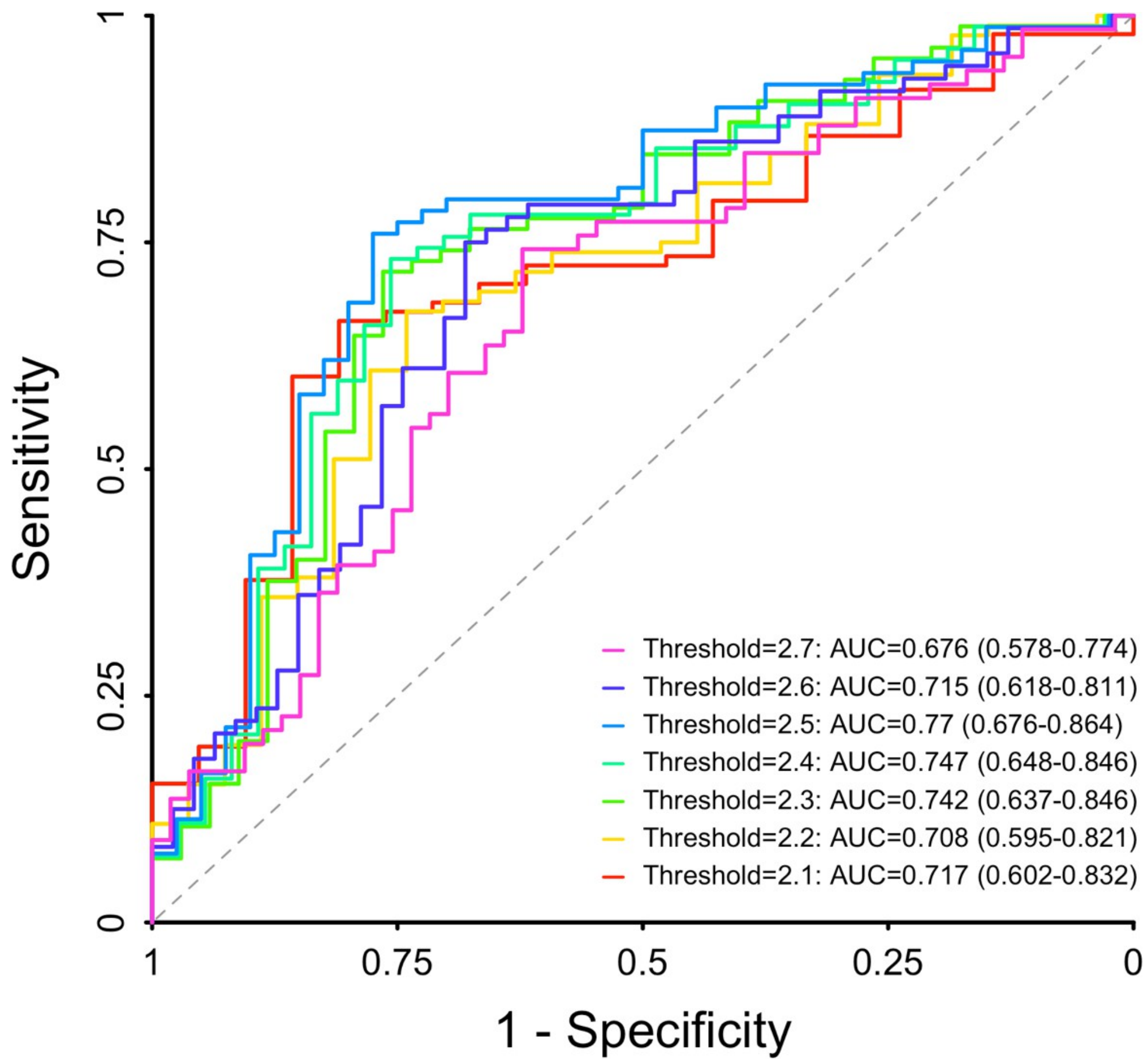

Figure E: Quantile regression Bland-Altman plot for microvascular resistance reserve (MRR) displaying quantile regression line equations
Data derived from 116 observations.

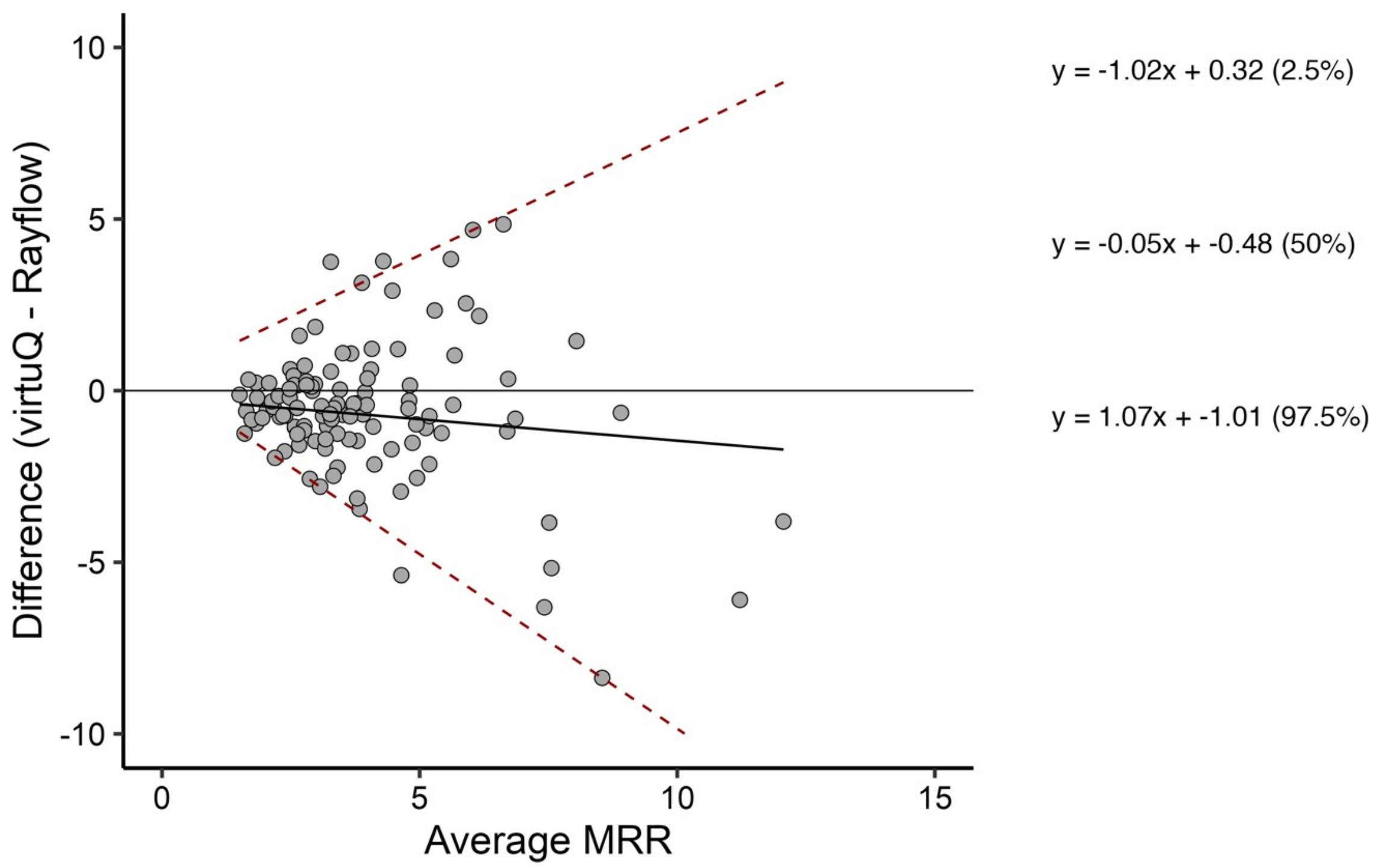

Table B: MRR SHAP analysis results

Average R^2 score values for training set (cross-validation train + test) and test set were:

Metrics	Statistics	Random Forest	Linear Regression
**Cross-validation train R2**	Mean	0.4607	0.3980
	Median	0.4589	0.3811
	Standard deviation	0.0300	0.0573
**Cross-validation test R2**	Mean	-0.0157	0.0686
	Median	-0.0051	0.0912
	Standard deviation	0.1225	0.1664
**Test R2 score**	Mean	0.1553	0.1849
	Median	0.2020	0.2451
	Standard deviation	0.2127	0.1998

These results suggested our MRR data was not suitable for SHAP analysis and was therefore not performed. Data derived from 116 observations.

Figure F: Agreement results for MRR stratified by ACS vs CCS cohorts
A) Correlation plot and simple least regression for Rayflow versus virtuQ derived MRR in ACS cohort (p value computed from Spearman's rank correlation test). B) Bland Altman plot demonstrating agreement between Rayflow and virtuQ MRR in ACS cohort. Median bias (solid line, y=-0.15x – 0.17) and 95% limits of agreement (dashed lines, y=-1.06x + 0.45 and y=1.28x - 1.83) derived from quantile regression. CCS data derived from 71 observations. C) Correlation plot and simple least regression for Rayflow versus virtuQ derived MRR in CCS cohort (p value computed from Spearman's rank correlation test). D) Bland Altman plot demonstrating agreement between Rayflow and virtuQ MRR. Median bias (solid line, y=0.05x – 0.67) and 95% limits of agreement (dashed lines, y=-0.46x – 0.95 and y=0.34x + 2.64) derived from quantile regression. ACS data derived from 45 observations. ACS, acute coronary syndrome; CCS, chronic coronary syndrome; MRR, microvascular resistance reserve.

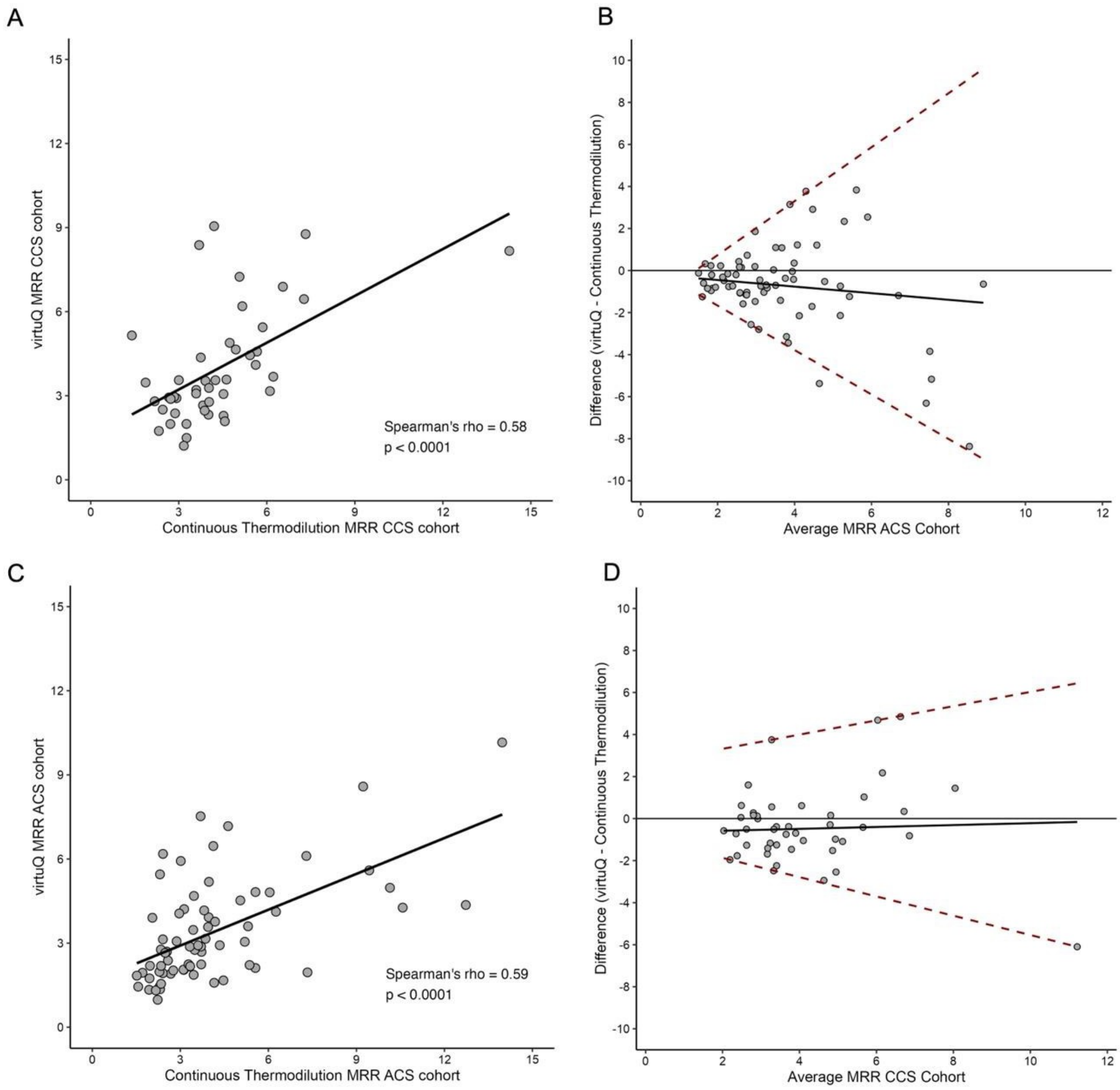

Figure G: Agreement results for MRR in FFR >0.85 cohort
A) Correlation plot and simple least regression for Rayflow versus virtuQ derived MRR (p value computed from Spearman's rank correlation test). B) Bland Altman plot demonstrating agreement between Rayflow and virtuQ MRR. Median bias (solid line, y= 0.63x – 2.02) and 95% limits of agreement (dashed lines, y=-1.36x + 0.93 and y=1.40x – 2.22) derived from quantile regression. Data derived from 37 observations. MRR, microvascular resistance reserve.

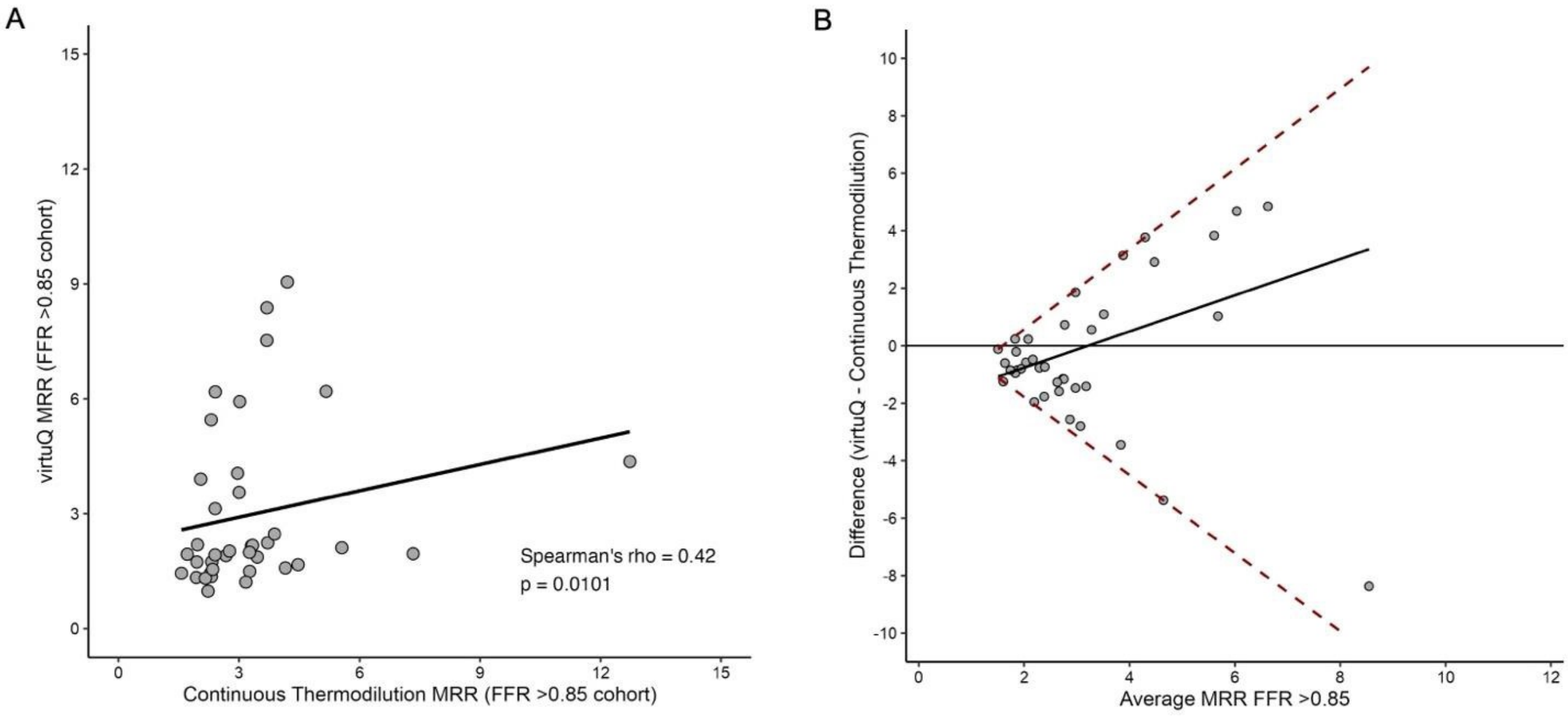

Text D: Rayflow/virtuQ hyperaemic flow Bland-Altman quantile regression line equations
Data derived from 131 observations and correspond to figure 3 panel D in main manuscript.

Q2.5: y = 0.034 * x + -118.37
Q50: y = 0.814 * x + -86.144
Q97.5: y = 1.391 * x + -7.047

Table C: Absolute inlet flow SHAP analysis results

Average R^2 score values for training set (cross-validation train + test) and test set were:

Metrics	Statistics	Random Forest	Linear Regression
**Cross-validation train R^2**	Mean	0.6568	0.6698
	Median	0.6548	0.6740
	Standard deviation	0.0326	0.0217
**Cross-validation test R^2**	Mean	0.5277	0.5228
	Median	0.5351	0.5351
	Standard deviation	0.0545	0.0631
**Test R^2 score**	Mean	0.6642	0.5668
	Median	0.7011	0.5995
	Standard deviation	0.1580	0.1234

Averaged SHAP results for Random Forest and Linear Regression in both the test set and fold test sets are shown on the subsequent two pages respectively. Data derived from 131 observations.

Random forest SHAP analysis for absolute flow:

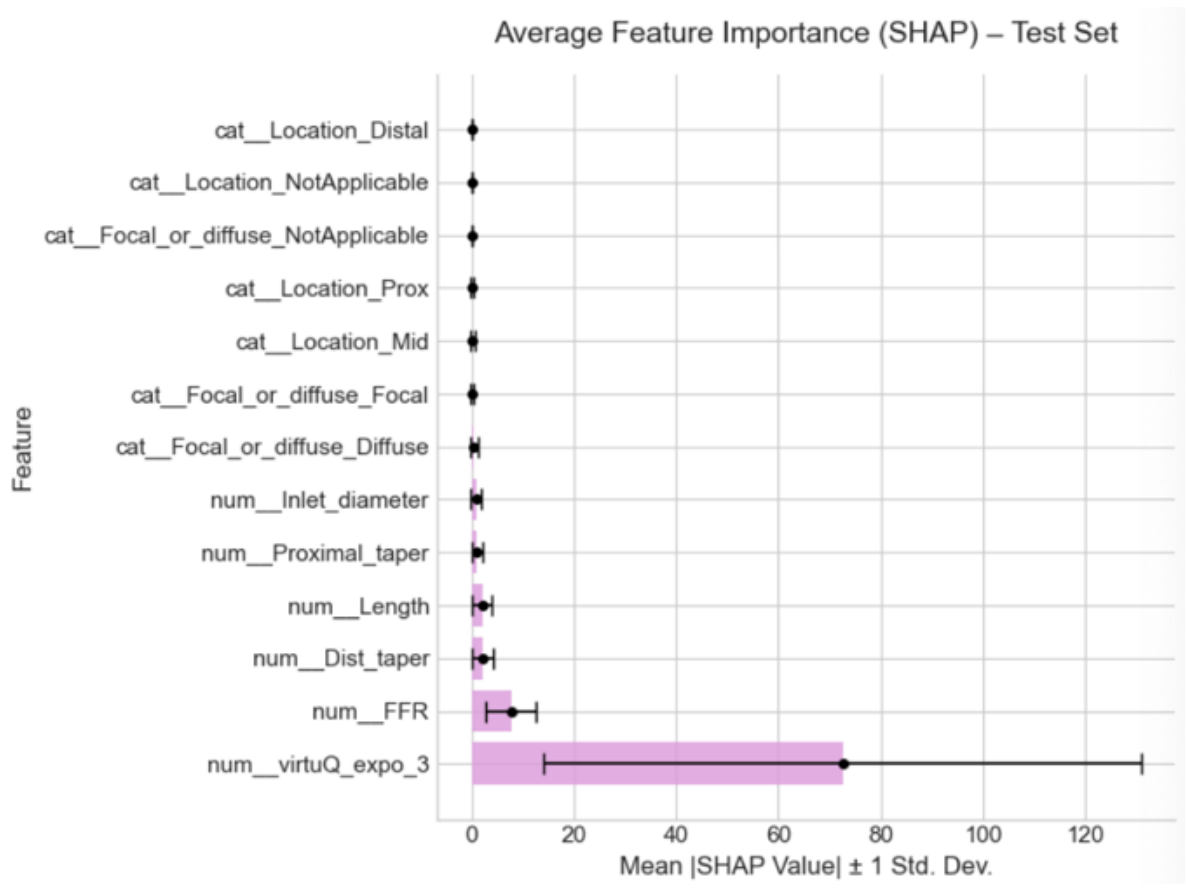

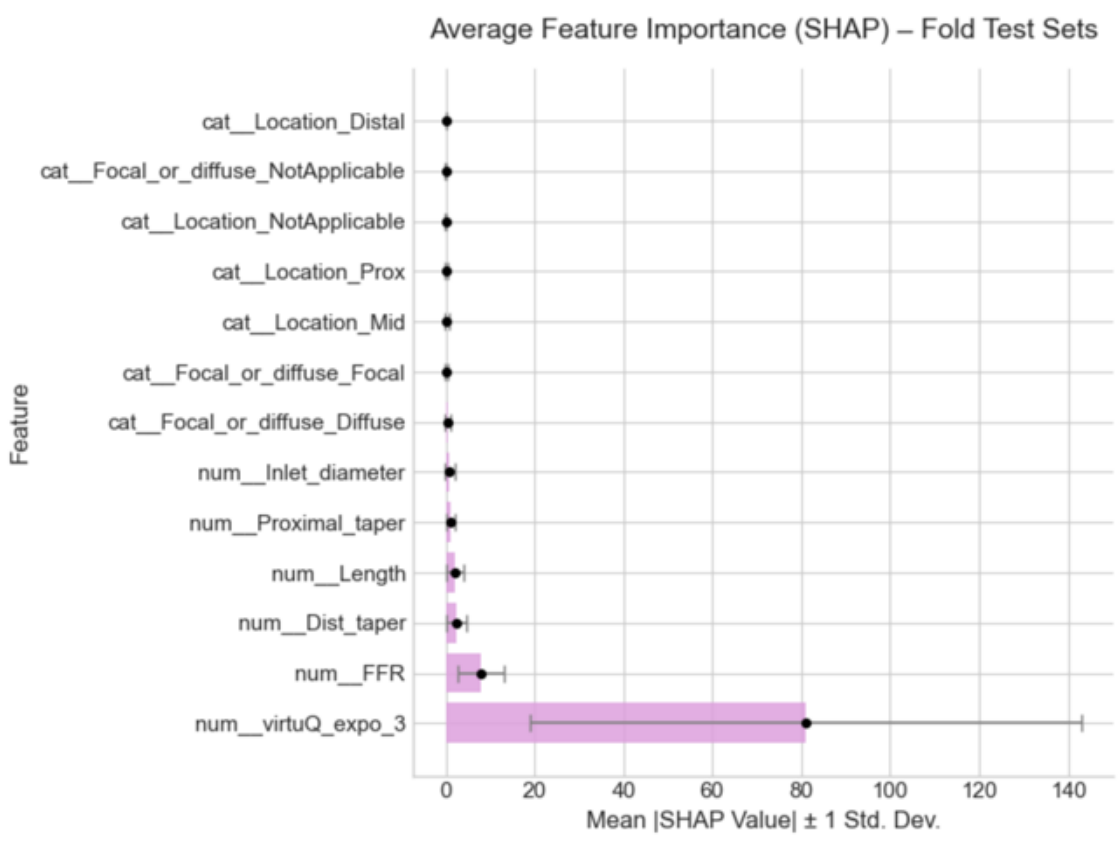

Number of runs included	44/60		Number of runs included	44/60	
Average score	0.5537		Average score	0.5537	
SHAP values (Filtered Runs)			SHAP values (Filtered Runs)		
Feature	Mean	Std	Feature	Mean	Std
num__virtuQ_expo_3	72.555	58.378	num__virtuQ_expo_3	80.239	61.204
num__FFR	7.702	4.954	num__FFR	7.689	4.769
num__Dist_taper	2.249	2.129	num__Length	2.345	2.452
num__Length	2.076	2.020	num__Dist_taper	1.962	2.056
num__Proximal_taper	1.060	0.991	num__Inlet_diameter	1.065	1.082
num__Inlet_diameter	0.860	1.033	num__Proximal_taper	0.750	0.676
cat__Focal_or_diffuse_Diffuse	0.498	0.744	cat__Focal_or_diffuse_Diffuse	0.369	0.513
cat__Focal_or_diffuse_Focal	0.204	0.312	cat__Location_Mid	0.243	0.317
cat__Location_Mid	0.194	0.408	cat__Focal_or_diffuse_Focal	0.178	0.234
cat__Location_Prox	0.152	0.236	cat__Location_Prox	0.053	0.106
cat__Focal_or_diffuse_NA	0.005	0.031	cat__Location_NA	0.037	0.141
cat__Location_NA	0.003	0.021	cat__Focal_or_diffuse_NA	0.000	0.000
cat__Location_Distal	0.000	0.000	cat__Location_Distal	0.000	0.000

Ridge Linear Regression SHAP analysis for absolute flow:

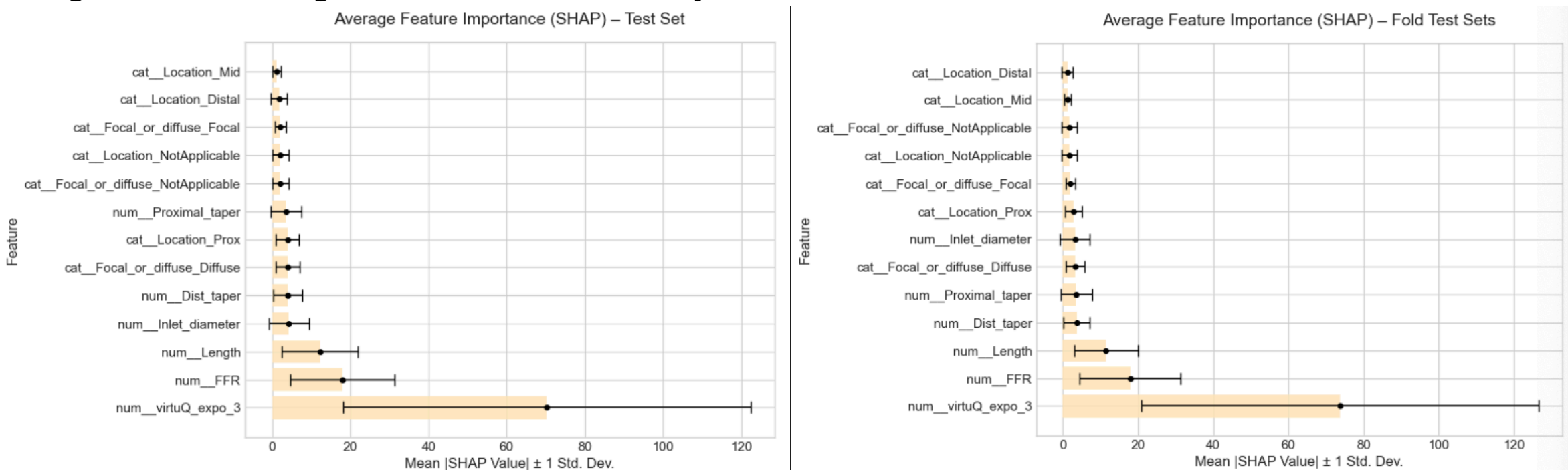

Number of runs included	39/60		Number of runs included	39/60	
Average score	0.5617		Average score	0.5617	
SHAP values (Filtered Runs)			SHAP values (Filtered Runs)		
Feature	Mean	Std	Feature	Mean	Std
num__virtuQ_expo_3	70.275	52.114	num__virtuQ_expo_3	73.660	52.767
num__FFR	17.921	13.415	num__FFR	17.964	13.421
num__Length	12.187	9.775	num__Length	11.537	8.506
num__Inlet_diameter	4.214	5.091	num__Dist_taper	3.738	3.462
num__Dist_taper	3.958	3.736	num__Proximal_taper	3.632	4.172
cat__Focal_or_diffuse_Diffuse	3.923	2.982	cat__Focal_or_diffuse_Diffuse	3.332	2.573
cat__Location_Prox	3.870	2.985	num__Inlet_diameter	3.266	4.003
num__Proximal_taper	3.462	3.902	cat__Location_Prox	2.957	2.229
cat__Focal_or_diffuse_NA	2.076	2.009	cat__Focal_or_diffuse_Focal	2.045	1.195
cat__Location_NA	2.076	2.009	cat__Location_NA	1.768	1.967
cat__Focal_or_diffuse_Focal	2.076	1.424	cat__Focal_or_diffuse_NA	1.768	1.967
cat__Location_Distal	1.686	2.078	cat__Location_Mid	1.252	0.897
cat__Location_Mid	1.191	1.085	cat__Location_Distal	1.216	1.536

Despite both methods producing similar results, the Random Forest had greater stability. We therefore concluded these were the more reliable results and thus presented in the main manuscript.

Figure H: Agreement results for hyperaemic flow stratified by ACS vs CCS cohorts

A) Correlation plot and simple least regression for Rayflow versus virtuQ derived hyperaemic flow in ACS cohort (p value computed from Spearman’s rank correlation test). B) Bland Altman plot demonstrating agreement between Rayflow and virtuQ hyperaemic flow in ACS cohort. Median bias (solid line, y=0.58x – 60.4) and 95% limits of agreement (dashed lines, y=0.18x – 135.9 and y=0.99x + 13.6) derived from quantile regression. ACS data derived from C) Correlation plot and simple least regression for Rayflow versus virtuQ derived hyperaemic flow in CCS cohort (p value computed from Spearman’s rank correlation test). D) Bland Altman plot demonstrating agreement between Rayflow and virtuQ hyperaemic flow. Median bias (solid line, y=0.98x – 108.7) and 95% limits of agreement (dashed lines, y=0.03x – 118.3 and y=1.94x – 66.7) derived from quantile regression. CCS data derived from 86 observations. ACS, acute coronary syndrome; CCS, chronic coronary syndrome.

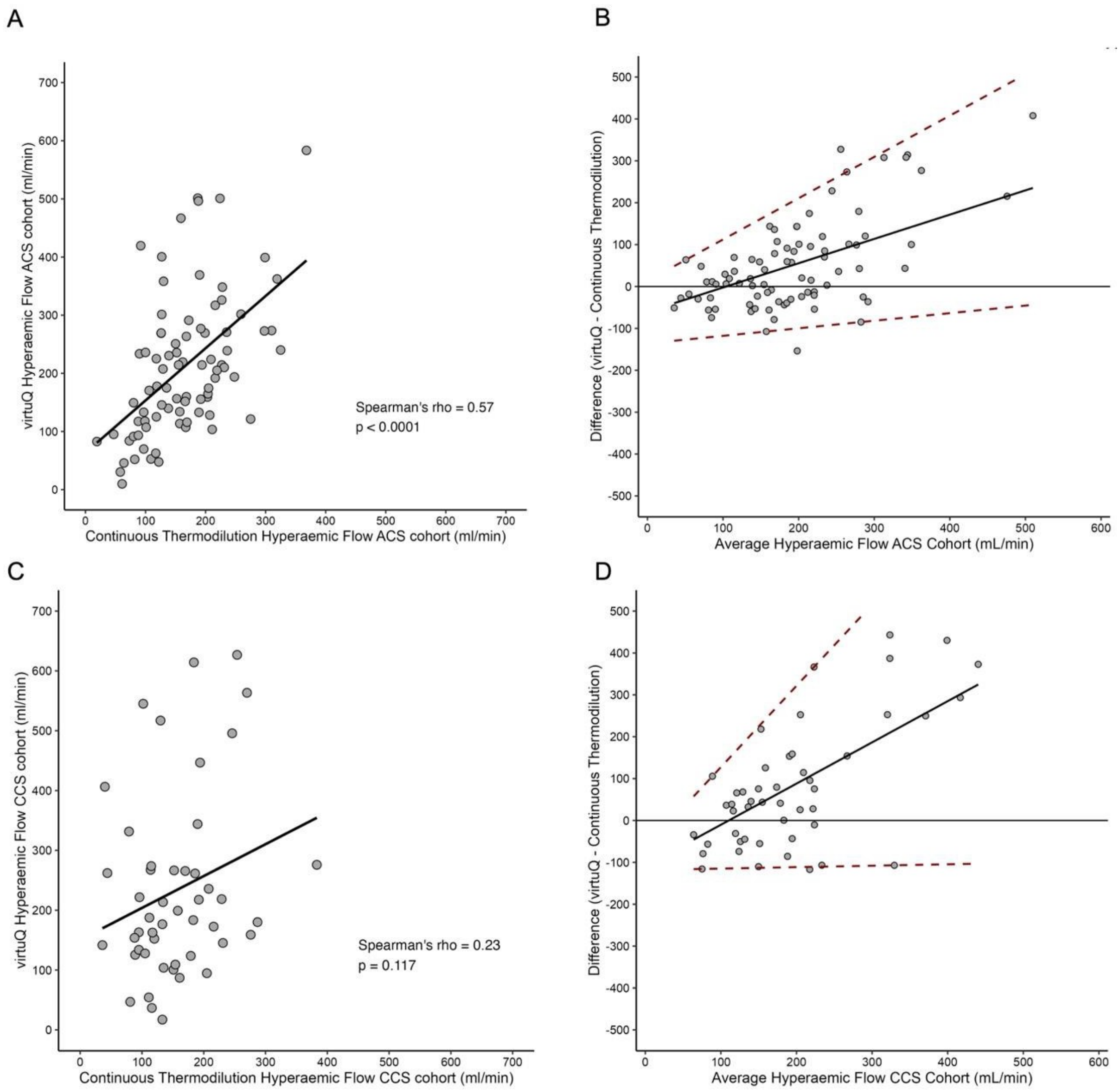

Figure I: Before and after dot plot for hyperaemic inlet flow
Flow-diameter scaling exponent 7/3 (2.33) versus 3.0. Data derived from 131 observations. P value derived using Wilcoxon signed-rank test.

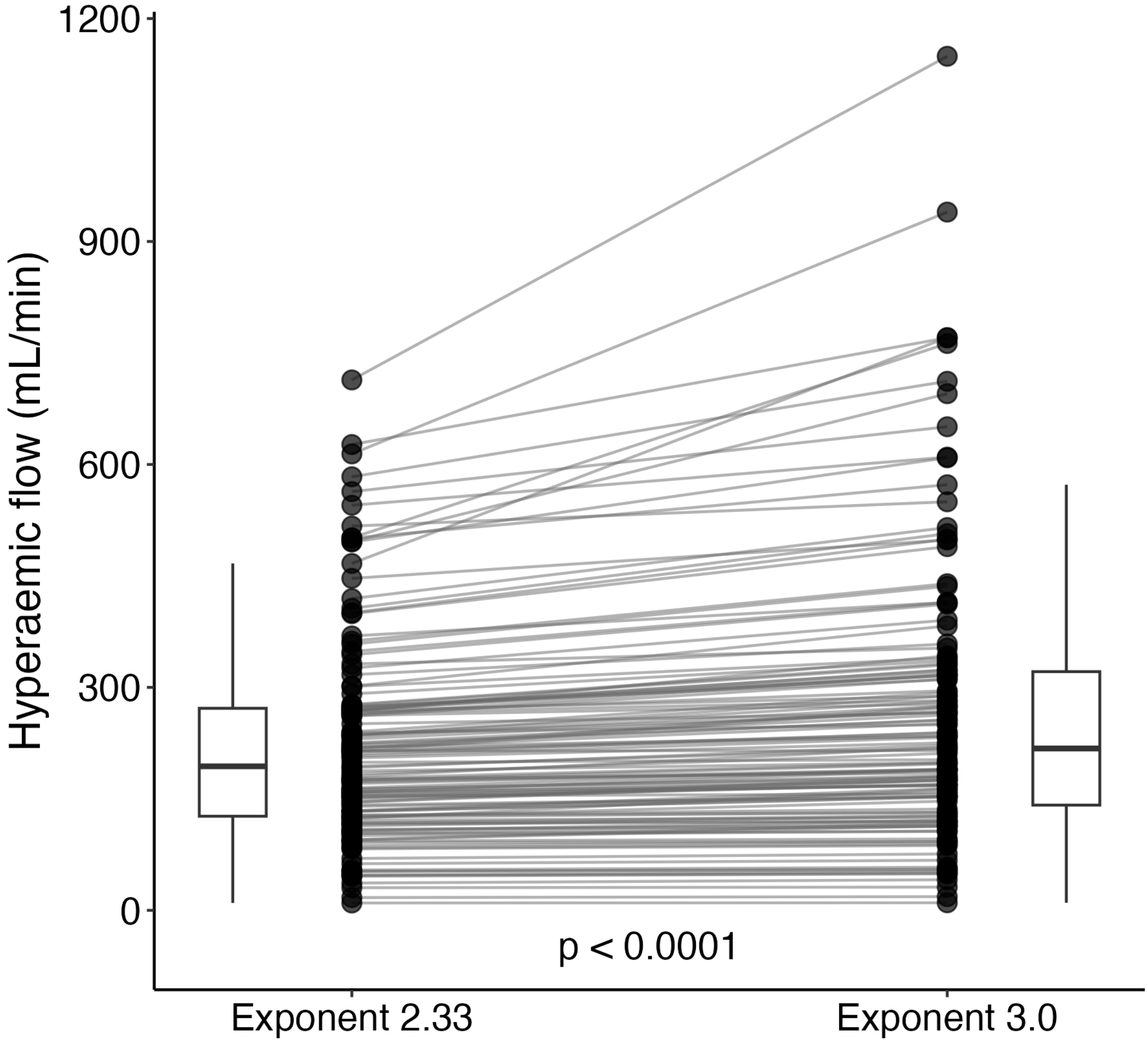

Figure J: Summary results for total resting flow
A) Histogram of invasively measured (Rayflow) flow under resting conditions. B) Histogram of computed (virtuQ) resting flow. C) Correlation plot and simple least regression for Rayflow versus virtuQ derived resting flow (p value computed from Spearman's rank correlation test). D) Bland Altman plot demonstrating agreement between Rayflow and virtuQ resting flow. Median bias (solid line, y=1.40x – 86.0) and 95% limits of agreement (dashed lines, y=0.10x – 88.5 and y=1.65x – 42.9) derived from quantile regression. Data derived from 120 observations.

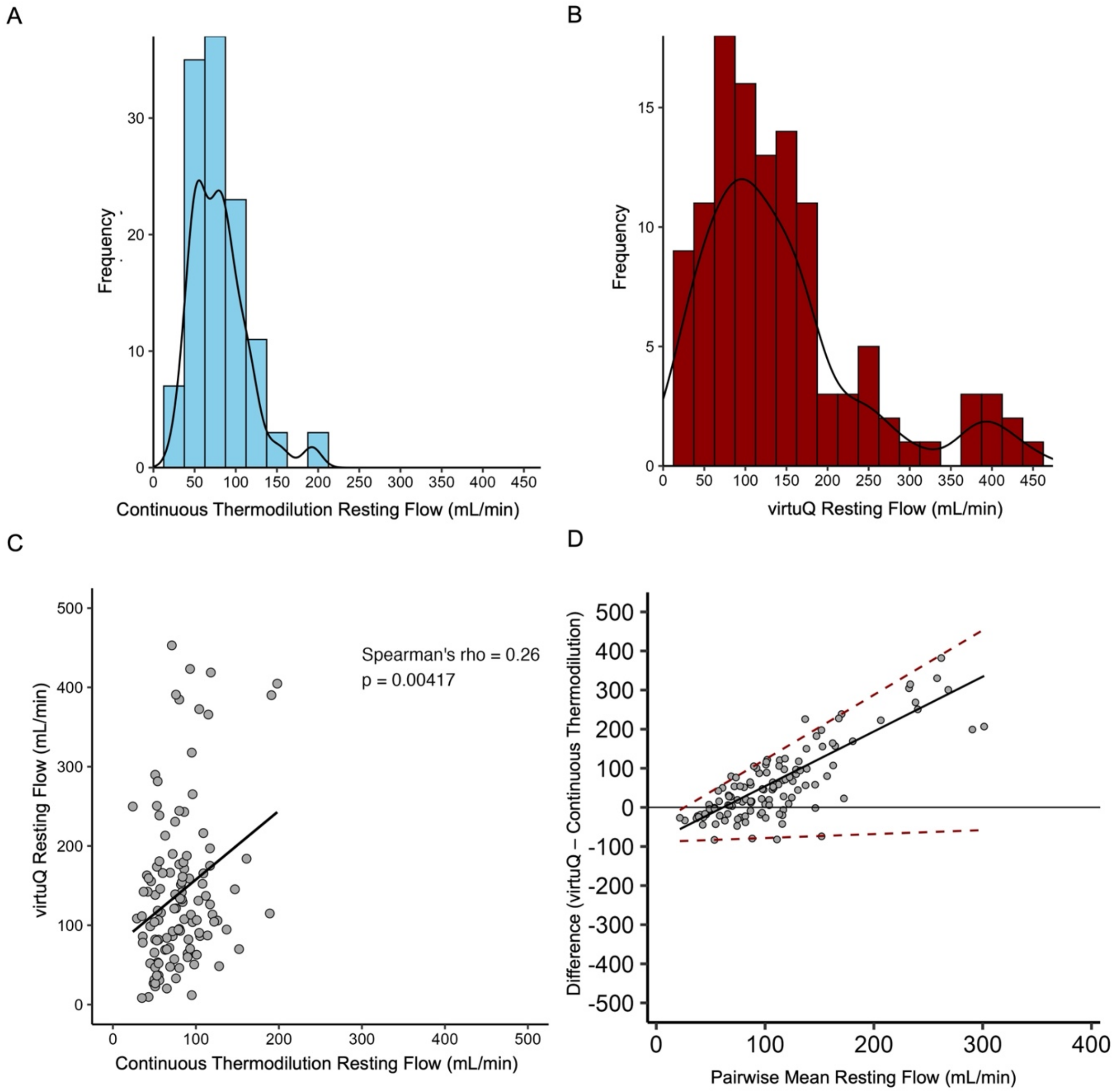

Figure K: Summary results for total vessel resistance
A) Histogram of invasively measured (Rayflow) resistance. B) Histogram of computed (virtuQ) resistance. C) Correlation plot and simple least regression for Rayflow versus virtuQ derived resistance (p value computed from Spearman's rank correlation test). D) Bland Altman plot demonstrating agreement between Rayflow and virtuQ derived resistance. Median bias (solid line, y = 0.69x -321.9) and 95% limits of agreement (dashed lines, y=-1.26x + 11.1 and y=1.75x + 327) derived from quantile regression. Data derived from 131 observations.

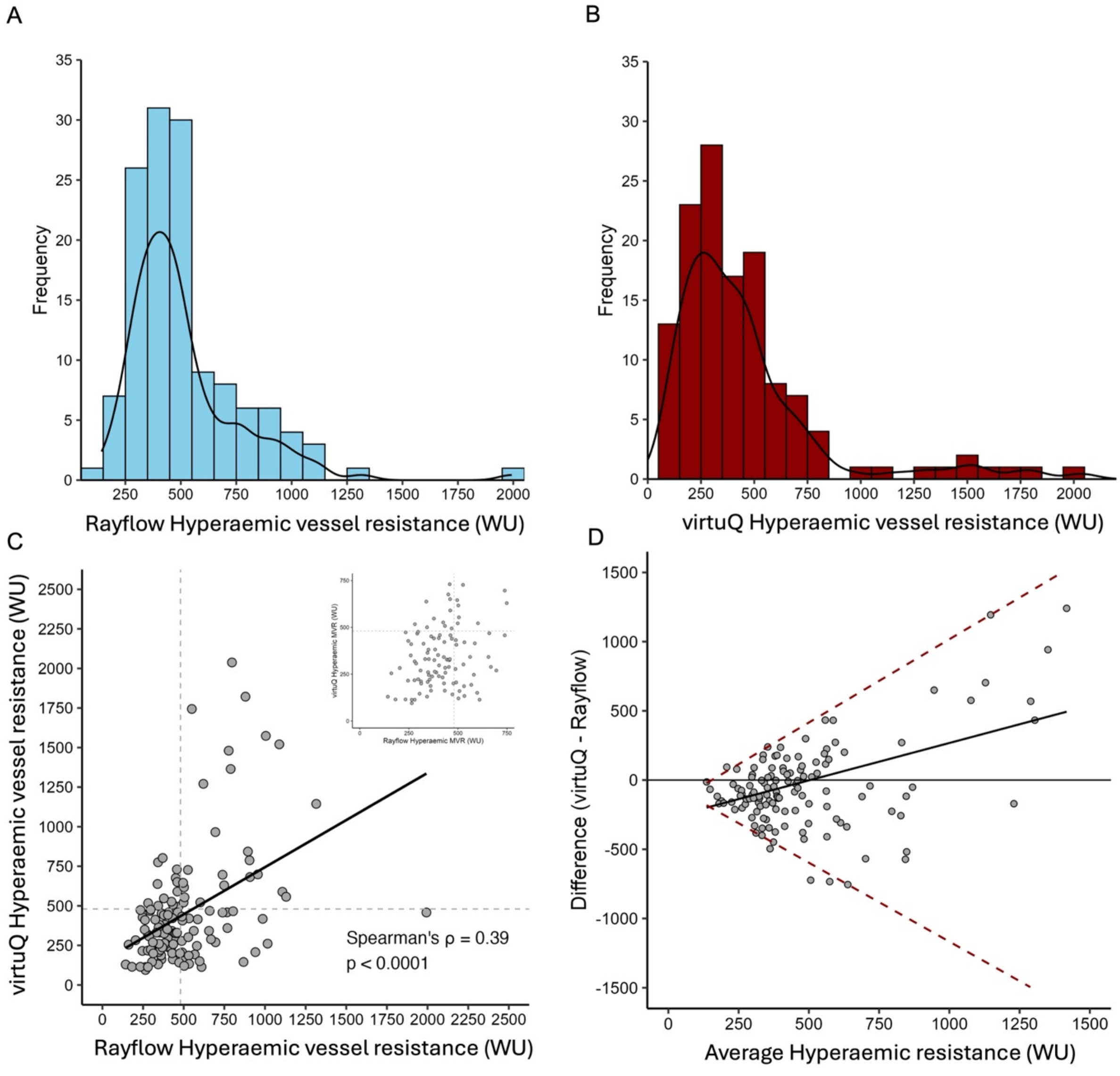

Figure L: Summary results for coronary flow reserve (CFR)
A) Histogram of invasively measured (Rayflow) CFR. B) Histogram of computed (virtuQ) CFR. C) Correlation plot and simple least regression for Rayflow versus virtuQ derived CFR (p value computed from Spearman’s rank correlation test). D) Bland Altman plot demonstrating agreement between Rayflow and virtuQ derived CFR. Median bias (solid line, y = 0.44x -1.06) and 95% limits of agreement (dashed lines, y=-1.47x + 1.14 and y=1.21x -1.15) derived from quantile regression. E) Receiver operator characteristic curve for resistance using a diagnostic threshold of 2.5, p value derived using DeLong’s method. Data derived from 116 observations. AUC, area under the curve

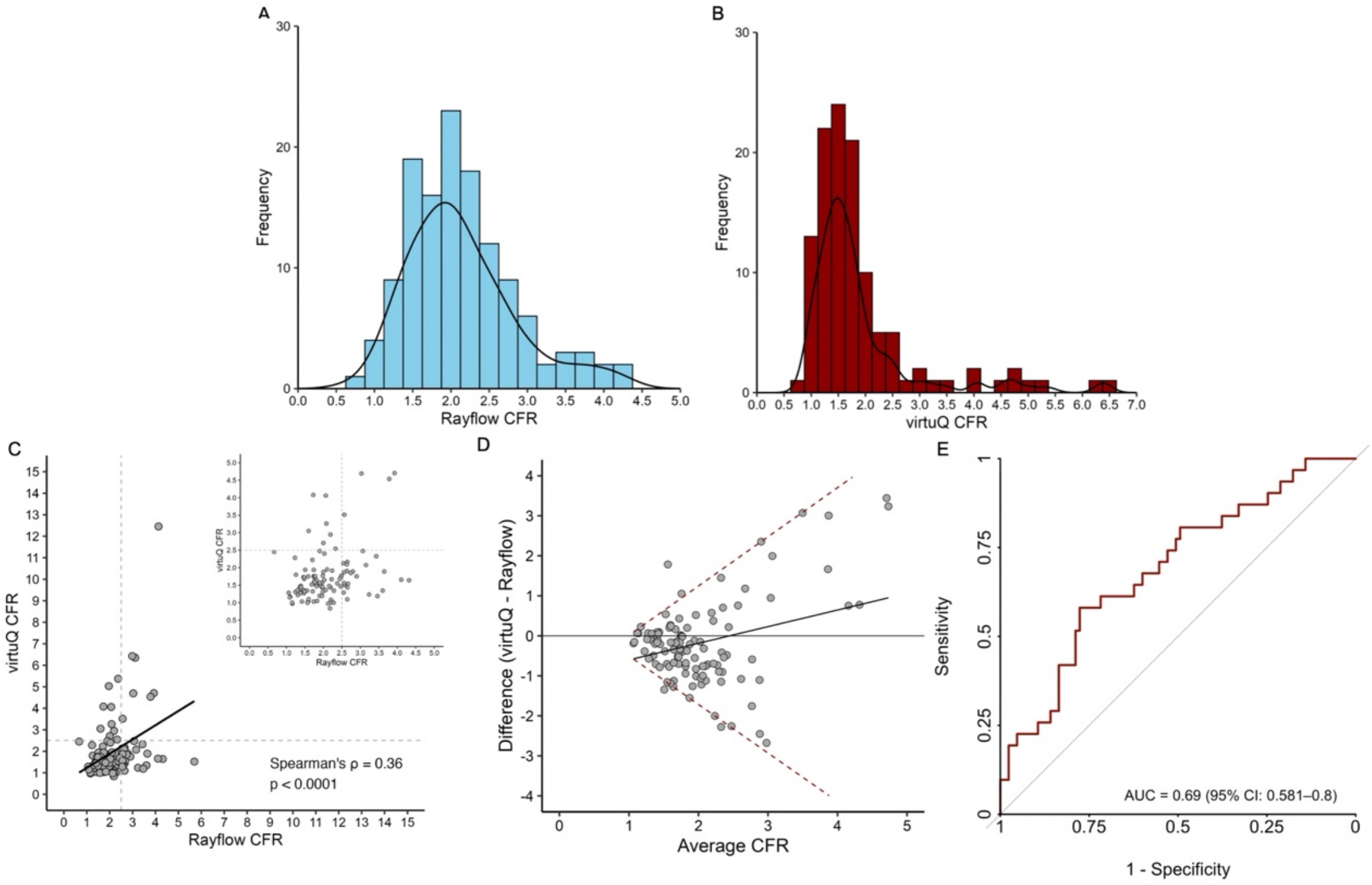

Figure M: ROC sensitivity analysis for coronary flow reserve (CFR)
Optimal diagnostic threshold 2.7. AUC p value derived using DeLong's method. Data derived from 116 observations.

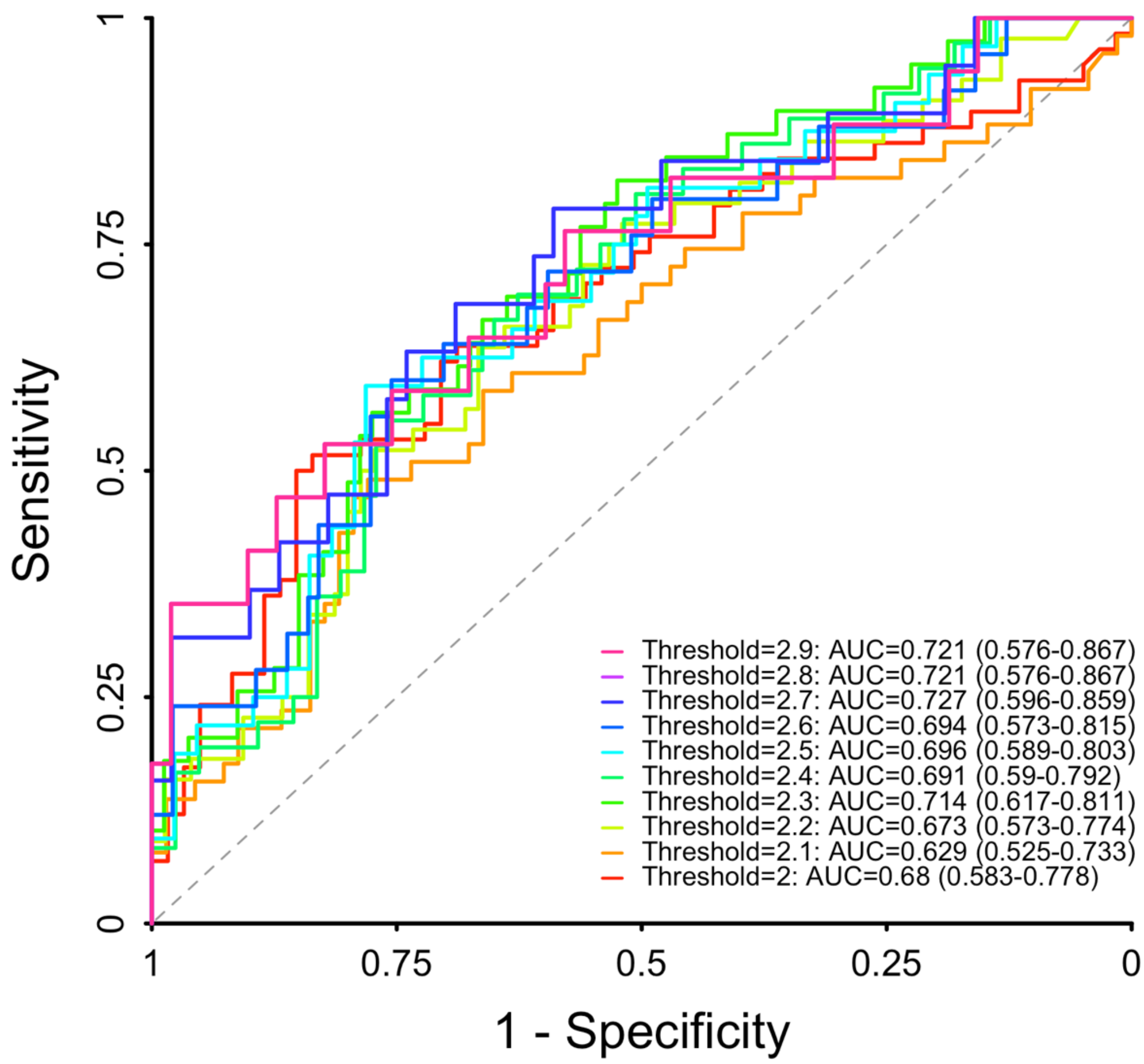

Supplement: Supplementary file 1 [file pdig.0001830.s001.pdf]
