# Supplementary material for: Agreement of an invasive angiography-derived computational model of coronary microvascular physiology against continuous thermodilution
Source: PLOS Digit Health. 2026 Nov 13;5(11):e0001830. doi: 10.1371/journal.pdig.0001830 (PMC13766283; doi:10.1371/journal.pdig.0001830)
Supplement: Supplementary file 5 [file pdig.0001830.s007.docx]

**Reviewer #1**

I have the pleasure to review the article entitled “Agreement and diagnostic accuracy of a computational model of coronary microvascular physiology against continuous thermodilution” by Daniel Taylor and colleagues.
I would like to congratulate the authors about the thorough work carried out in this project. Overall, the manuscript is well written and addresses an important topic of ongoing research (less invasive diagnostic techniques for assessing CMD).
I have some comments that I think should have to be taken into consideration:

**Comment 1:** In the title and abstract, it is not clear to readers how the computational indices are calculated. In my opinion, mentioning that VirtuQ is based upon computational angiography and FFR is needed.

**Response:** The title and abstract have been clearer regarding the methods underpinning virtuQ. We have now amended the title to:

‘*Agreement of an invasive angiography-derived computational model of coronary microvascular physiology against continuous thermodilution’*

In the methods section of the abstract, we now specify:

‘*Invasive coronary angiographic images were used to reconstruct 3D anatomy in-silico*.’

And:

‘*Invasive pressure measurements, recorded during fractional flow reserve assessment, informed the CFD boundary conditions.*’

**Comment 2.** I think CFD-MRR and invasive thermodilution-MRR should be labelled differently to improve clarity and avoid confusion throughout the text (e.g v-MRR for VirtuQ-calculated and t-MRR for continuous thermodilution). Also, for other indices (CFR, absolute flow, etc).

**Response:** We now distinguish these methods using subscripts _Virtu_ and _CIT_. This change is made clear in the methods section:

*‘The subscripts _CIT_ and _Virtu_ are hereon used to denote haemodynamic values measured with the [continuous infusion thermodilution] method and computed with virtuQ respectively.’* (lines 274-276)

**Comment 3.** In line 111, I think that saying that FFR does not give any information about the microcirculation is not correct. Although is mainly used to assess epicardial CAD, FFR values can also be affected in some degree by downstream coronary microcirculation function, mainly hyperaemic resistance (DOI: 10.1136/heartjnl-2013-305124). So, in my opinion, this statement is too categorical.

**Response:** We agree with the reviewer this statement was somewhat sweeping but would stress FFR does not give *useable* information regarding the microvasculature. This sentence has been reworked to give a more nuanced account of the interplay between FFR and the coronary microcirculation:

*‘While FFR is influenced both by anatomy (stenosis severity) and physiology (coronary flow), it does not yield any focused assessment of the coronary microcirculation (4).’* (lines 182 – 184)

**Comment 4.** Was the inclusion of patients done in a prospective fashion or was it a retrospective analysis from a cohort of patients that underwent continuous thermodilution assessment of CMD?

**Response:** All patients were prospectively enrolled into the OxAMI and Essex-SAAMI studies, as is stated in the methods section ‘Patient recruitment and clinical data collection’. The reviewer has correctly highlighted this as an important aspect of study design and have such included the prospective nature of enrolment in the abstract.

**Comment 5.** Regarding the methods, do the authors included patients with CTOs? In that case, how can they deal with collateral branches arising from interrogated vessels? Was that considered in VirtuQ calculations?

**Response:** Patients with CTO were excluded and this is now made clear in the methods section.

**Comment 6.** In patients where ACS was the clinical presentation, was interrogation done only in non-culprit vessels? How much time passed from the clinical presentation (ACS) until physiological assessment? And, were both measurements (VirtuQ and continuous thermodilution) done at the same time? This issue is important, and I think it should be stressed in the discussion: how the dynamic changes in intracoronary physiology after an ACS may influence a measurement that relies on FFR.

**Response:** For patients with ACS, flow measurements were taken during the index angiogram and included both culprit and non-culprit vessels. We apologise this was not clear and has been amended in the methods.

‘*In patients presenting with ACS, flow was recorded in both culprit and non-culprit vessels during the index angiogram.*’ (lines 256 – 258)

As this was an offline analysis, virtuQ simulations were performed after invasive measurements were taken. As these pressures are used directly to inform CFD boundary conditions, there should be no introduction of lead time bias even if virtuQ is used months to years after the invasive measurements. We do however agree that the dynamic microvascular changes after an acute insult are an important clinical consideration when assessing coronary flow which were not given sufficient credence in the discussion. We have now included this specifically, considering the implications for virtuQ:

*‘However, during and shortly after an acute insult, the coronary microvasculature undergoes dynamic changes which influence physiology results. This complicates dichotomisation of patients, during a single invasive catheterisation, as having a favourable versus unfavourable physiological profile. Under circumstances where acute MVD restricts flow, FFR is likely to increase, and this would reduce accuracy of virtuQ.’* (lines 586 – 598)

**Comment 7.** Do the authors have an explanation on why computed MRR and computed CFR tend to be lower than continuous thermodilution-based measurements? In the discussion, it is hypothesized that the relatively high proportion of ACS patients may have biased the measurements (by increasing the prevalence of cases with low MRR). However, if both measurements were done at the same time, the transient abnormalities in the microcirculation secondary to a recent ACS should affect both indices.

**Response:** We feel this discrepancy more likely lies in the physics underpinning virtuQ, specifically the inclusion of only dynamic pressure losses. This decision was a conscious effort to minimise system complexity and account for major determinants of flow (1), in addition to speeding up virtuQ simulations. However, these results suggest the increase in system complexity is warranted, especially for CFR calculations which we now address in the discussion:

*‘The lack of inclusion of static pressure may be particularly important for CFR and MRR calculations; omitting this from simulations is more likely to introduce error when total pressure gradients (Pa minus Pd) are low. This disproportionately affects baseline flow calculations and consequently has a heightened influence over CFR and MRR calculations versus Q and Rμ which are derived solely from hyperaemic pressure measurements.’* (lines 545 – 558)

We have also updated the methods section to improve the clarity of simulation specifications:

‘*Simulations considered only dynamic and not static or gravitational pressure losses.*’ (line 327)

**Comment 8.** In this study, a high proportion of patients had positive FFR, something that it is not that common in most patients referred for invasive assessment of CMD (e.g. patients without epicardial disease or mild/moderate stenoses). This makes, in my opinion, the results less comparable to everyday practice in most centers. I think it should be stressed in the title and/or abstract that this study was carried out mostly in patients with epicardial CAD.

**Response:** We agree with the reviewer our cohort is less typical of patients who would undergo microvascular assessment in most centres, although emerging data suggest structural MVD is *more* prevalent in those with epicardial stenoses than those without (2). Nevertheless, to improve clarity of the cohort’s composition, we have included the following in the abstract results:

*‘Median FFR was 0.81 [0.70 – 0.89] and 108 (81%) vessels contained an epicardial stenosis.’*

**Comment 9.** In figure 3 is concerning the drop in classification agreement around the cutoff of 2.5, that should be the critical point where most cases will be. How can the authors explain this?

**Response:** We feel this is not a concerning finding and rather reflects an inevitability of validating a test of dichotomisation. For any imperfect method, classification exactly at a cutoff is a coin flip by construction; a case is misclassified whenever measurement error exceeds the distance between its true value and the threshold. For a vessel who’s true (CIT) MRR is exactly 2.5, the tolerance for virtuQ to over-estimate while retaining the correct classification of abnormal microvascular physiology is zero. A trough centred on the threshold is not, to our understanding, a concerning finding and is not the primary aim of this display figure. Instead, the figure is included to give a visualisation of diagnostic accuracy at values which lie *away* from the diagnostic threshold. For instance, how much confidence should a clinician put in a virtuQ MRR value of 2.6 versus 3.2 versus 4.8? We have now clarified this issue in the discussion:

‘*While diagnostic accuracy is inevitably weakest at the diagnostic threshold, error increases the number of grey zone cases where confidence in virtuQ for confidently ruling MVD in or out is low.*’ (lines 516 – 518)

**Reviewer #2**

In the current manuscript, the authors aimed to validate a computational model of coronary microvascular physiology in-vivo. They compared 131 arteries from 89 patients with chronic and acute coronary syndromes undergoing continuous thermodilution and angiography-derived assessment by using three-dimensional computational fluid dynamics (3D CFD). The median computed microvascular resistance reserve (MRR) of 2.31 [1.83 – 3.00] was significantly lower than the median thermodilution assessed MRR of 2.79 [2.21 – 3.52] (z = 3.57, p = 0.0004). Moderate relationships were found between both techniques for determining MRR and hyperemic vessel inlet flow and agreement between both techniques improved at lower values.
A few major and minor comments should be addressed to improve the quality of the manuscript.
Major comments:

**Comment 1:** There doesn’t seem to be an exaggerated difference between computed MRR and thermodilution assessed MRR. However, in this population, median values were respectively 2.31 and 2.79 for both techniques. With a pre-specified MRR threshold of ≤2.5, there seem to be an impact on classification of patients in normal or abnormal values. There is no clear discussion about that.

**Response:** We agree this is an important issue which was not given sufficient attention in the discussion. We have now made the implications of this systematic bias clearer:

*‘At the diagnostic threshold overall bias was 24 ±40% and while this is comparable with recent validation studies of invasive techniques, it represents a systematic underestimation of MRR_CIT_ by MRR_Virtu_ of around 0.4. In practice, this bias increases the proportion of patients with abnormal results when virtuQ is used and makes the CFD method a more appropriate tool for ruling MVD out versus in (PPV 38% versus NPV 87%). While diagnostic accuracy is inevitably weakest at the diagnostic threshold, error increases the number of grey zone cases where confidence in virtuQ for confidently ruling MVD in or out is low.’* (lines 496 – 518)

**Comment 2:** One of the major advantages of CFD highlighted by the authors is to decrease instrumentation and need for invasive measurements. There are 2 remarks on that statement. The first is that one still needs an invasive distal pressure measurement. The second is that microcirculation assessment during ANOCA, NSTEMI and STEMI are debated. In a great number of cases, patients will have to come back a second time to the cathlab for microcirculation assessment in stable conditions. This specific advantage of CFD should discussed in more details.

**Response:** We agree the requirement for a pressure wire poses a barrier to widespread use of virtuQ and this is acknowledged in the limitations section. However, personalising CFD boundary conditions with patient-specific invasive measurements will increase simulation accuracy and ‘unlocks’ the microvascular influence contained within FFR, We have now included further discussion of this consideration:

‘*While in-procedure (online) virtuQ assessment may be performed faster than CIT measurements can be taken, the CFD method still requires a pressure wire. This requirement for coronary instrumentation poses a barrier to routine assessment in all vessels, especially for diagnostic only catheterisation laboratories. Alternative wire-free computational methods have been described, where flow is inferred from TIMI frame counting. While comparable diagnostic accuracy results have been reported in a similar cohort to the current study (40), vessel foreshortening and geometric reconstruction errors contribute uncertainty to estimated flow.*’ (lines 605 – 613)

Please see our response to reviewer 1 comment 6 for our response and revision to the manuscript regarding timing of microvascular assessment in the context of ACS.

Minor comments:
**Comment 3:** At line 441-442 the author emphasizes that computed CFD may be less accurate in patients with angina with non-obstructive coronary arteries (ANOCA). However, those patients seem to be the main target population. This should be highlighted.

**Response:** We have acknowledged this as a limitation of the CFD technique. Nevertheless, we have added further discussion of this important limitation, but have retained contextualisation against emerging evidence suggesting MVD is prevalent and of equal clinical significance in patients with epicardial disease:

‘*The current results, where agreement between CIT and virtuQ measurements weakens in vessels lacking an appreciable epicardial stenosis, suggest the CFD method may not be suitable for microvascular assessment in a population enriched by patients with suspected ANOCA. However, the assumption of a predominant phenotype where pathology lies exclusively in the microvascular compartment appears biologically unlikely; large registry data has shown MRR to be a stronger independent predictor of major adverse cardiac events (MACE) in patients with versus without obstructive epicardial stenoses (3)*.’ (lines 570 – 578)

**Comment 4:** Producing suitable images for computed analysis includes more contrast use. This is not mentioned in this article. From the total of 178 arteries, only 116 were selected and analyzed, showing that good image quality is needed. This could potentially increase procedural contrast use.

**Response:** While this may theoretically be a limitation, virtuQ requires only two high-quality angiographic projects and these would be captured during a typical angiography procedure. We have now acknowledged this as a potential limitation:

[virtuQ requires] ‘*two high-quality angiographic projections may theoretically increase contrast use’* (lines 629 – 630).

1.	Eck VG, Donders WP, Sturdy J, Feinberg J, Delhaas T, Hellevik LR, et al. A guide to uncertainty quantification and sensitivity analysis for cardiovascular applications. International journal for numerical methods in biomedical engineering. 2016;32(8):e02755.

2.	Lee JM, Lee SH, Gim DH, Lee SY, Choi KH, Ahn JH, et al. Coronary microvascular dysfunction and cardiovascular outcomes (Multicenter FLOW-CMD Registry): a prospective, multicentre cohort study in South Korea. The Lancet. 2026;407(10545):2319-28.

3.	Boerhout CKM, Lee JM, de Waard GA, Mejia-Renteria H, Lee SH, Jung J-H, et al. Microvascular resistance reserve: diagnostic and prognostic performance in the ILIAS registry. European Heart Journal. 2023;44(30):2862-9.

**Journal Requirements:**

1. Please ensure that your Ethics Statement is available in its entirety at the beginning of your Methods section, under a subheading 'Ethics Statement'. **Confirmed**

2. Please amend your detailed Financial Disclosure statement. This is published with the article. It must therefore be completed in full sentences and contain the exact wording you wish to be published.

a. Please clarify all sources of funding (financial or material support) for your study. List the grants (with grant number) or organizations (with url) that supported your study, including funding received from your institution. 
b. State the initials, alongside each funding source, of each author to receive each grant.
c. State what role the funders took in the study. If the funders had no role in your study, please state: “The funders had no role in study design, data collection and analysis, decision to publish, or preparation of the manuscript.”
d. If any authors received a salary from any of your funders, please state which authors and which funders.

**Response:** Financial disclosures now reads:

*‘For the purposes of this study, DJT was funded by the National Institute for Health and Care Research (NIHR) Sheffield Biomedical Research Centre (BRC). The views expressed are those of the author(s) and not necessarily those of the NIHR or the Department of Health and Social Care. XX, KC, IH, JG and PDM were funded by EPSRC Health Technologies Connectivity Awards (UKRI830). The funders had no role in study design, data collection and analysis, decision to publish, or preparation of the manuscript.’*

3. Please provide separate figure files in .tif or .eps format. **Confirmed**

4. Please provide an Author Summary. This should appear in your manuscript between the Abstract (if applicable) and the Introduction, and should be 150–200 words long. The aim should be to make your findings accessible to a wide audience that includes both scientists and non-scientists. Sample summaries can be found on our website under Submission Guidelines: <https://journals.plos.org/digitalhealth/s/submission-guidelines#loc-parts-of-a-submission>. **Now added**

5. We have noticed that you have uploaded Supporting Information files, but you have not included a list of legends. Please add a full list of legends for your Supporting Information files after the references list. **Now added**

6. We note that your Data Availability Statement is currently as follows: "All data are provided in the manuscript and supplementary material"
 
 Please confirm at this time whether or not your submission contains all raw data required to replicate the results of your study. Authors must share the “minimal data set” for their submission. PLOS defines the minimal data set to consist of the data required to replicate all study findings reported in the article, as well as related metadata and methods.
   
<https://journals.plos.org/digitalhealth/s/data-availability#loc-minimal-data-set-definition>

 For example, authors should submit the following data:
 
 - The values behind the means, standard deviations and other measures reported;
 - The values used to build graphs;
 - The points extracted from images for analysis.
 
 Authors do not need to submit their entire data set if only a portion of the data was used in the reported study.
 
 If your submission does not contain these data, please either upload them as Supporting Information files or deposit them to a stable, public repository and provide us with the relevant URLs, DOIs, or accession numbers. For a list of recommended repositories, please see:

<https://journals.plos.org/digitalhealth/s/recommended-repositories>

 If there are ethical or legal restrictions on sharing a de-identified data set, please explain them in detail (e.g., data contain potentially sensitive information, data are owned by a third-party organization, etc.) and who has imposed them (e.g., an ethics committee). Please also provide contact information for a data access committee, ethics committee, or other institutional body to which data requests may be sent. If data are owned by a third party, please indicate how others may request data access.

**Response:** We have now updated the data availability statement in line with journal requirements:

‘*The data used in this study were sourced from patients prospectively enrolled into the OxAMI (Oxford Acute Myocardial Infarction) and Essex-SAAMI (Essex Stable Angina and Acute Myocardial Infarction) studies. The OxAMI and Essex-SAAMI studies are both ongoing cohort studies and according to institutional requirements, the raw patient-level data cannot be publicly released. Qualified researchers may request access to patient-level data via oxami@cardiov.ox.ac.uk for the OxAMI study and thomas.keeble2@nhs.net for the Essex-SAAMI study.*’

We have also uploaded stl files for all coronary reconstructions. All other data have been provided in the supplementary pdf.

7. Some material included in your submission may be copyrighted. According to PLOS’s copyright policy, authors who use figures or other material (e.g., graphics, clipart, maps) from another author or copyright holder must demonstrate or obtain permission to publish this material under the Creative Commons Attribution 4.0 International (CC BY 4.0) License used by PLOS journals. Please closely review the details of PLOS’s copyright requirements here: PLOS Licenses and Copyright. If you need to request permissions from a copyright holder, you may use PLOS's Copyright Content Permission form.

Please respond directly to this email or email the journal office and provide any known details concerning your material's license terms and permissions required for reuse, even if you have not yet obtained copyright permissions or are unsure of your material's copyright compatibility.

Potential Copyright Issues:

"Graphical Abstract": Please confirm (a) that you are the photographer; or (b) provide written permission from the photographer to publish the photo(s) under our CC-BY 4.0 license.

**Response:** There is no copyright infringement, the author is the owner of all images used to compose the graphical abstract.
