# Supplementary material for: Agreement of an invasive angiography-derived computational model of coronary microvascular physiology against continuous thermodilution
Source: PLOS Digit Health. 2026 Nov 13;5(11):e0001830. doi: 10.1371/journal.pdig.0001830 (PMC13766283; doi:10.1371/journal.pdig.0001830)
Supplement: Peer review history

# Peer review: Decision Letter 0

**DOI:** 10.1371/journal.pdig.0001830.r001

**Authors:** Peter H Charlton, Haibo Ni

26 Aug 2026

Response to Reviewers Revised Manuscript with Track Changes Manuscript **Journal Requirements:**

1. Please ensure that your Ethics Statement is available in its entirety at the beginning of your Methods section, under a subheading 'Ethics Statement'.

2. Please amend your detailed Financial Disclosure statement. This is published with the article. It must therefore be completed in full sentences and contain the exact wording you wish to be published.

a. Please clarify all sources of funding (financial or material support) for your study. List the grants (with grant number) or organizations (with url) that supported your study, including funding received from your institution.

b. State the initials, alongside each funding source, of each author to receive each grant.

c. State what role the funders took in the study. If the funders had no role in your study, please state: “The funders had no role in study design, data collection and analysis, decision to publish, or preparation of the manuscript.”

d. If any authors received a salary from any of your funders, please state which authors and which funders.

3. Please provide separate figure files in .tif or .eps format.

For more information about figure files please see our guidelines:  https://journals.plos.org/digitalhealth/s/figures

https://journals.plos.org/digitalhealth/s/figures#loc-file-requirements

4. Please provide an Author Summary. This should appear in your manuscript between the Abstract (if applicable) and the Introduction, and should be 150–200 words long. The aim should be to make your findings accessible to a wide audience that includes both scientists and non-scientists. Sample summaries can be found on our website under Submission Guidelines: https://journals.plos.org/digitalhealth/s/submission-guidelines#loc-parts-of-a-submission

5. We have noticed that you have uploaded Supporting Information files, but you have not included a list of legends. Please add a full list of legends for your Supporting Information files after the references list.

6. We note that your Data Availability Statement is currently as follows: "All data are provided in the manuscript and supplementary material"

Please confirm at this time whether or not your submission contains all raw data required to replicate the results of your study. Authors must share the “minimal data set” for their submission. PLOS defines the minimal data set to consist of the data required to replicate all study findings reported in the article, as well as related metadata and methods.

https://journals.plos.org/digitalhealth/s/data-availability#loc-minimal-data-set-definition

For example, authors should submit the following data:

- The values behind the means, standard deviations and other measures reported;

- The values used to build graphs;

- The points extracted from images for analysis.

Authors do not need to submit their entire data set if only a portion of the data was used in the reported study.

If your submission does not contain these data, please either upload them as Supporting Information files or deposit them to a stable, public repository and provide us with the relevant URLs, DOIs, or accession numbers. For a list of recommended repositories, please see:

https://journals.plos.org/digitalhealth/s/recommended-repositories

If there are ethical or legal restrictions on sharing a de-identified data set, please explain them in detail (e.g., data contain potentially sensitive information, data are owned by a third-party organization, etc.) and who has imposed them (e.g., an ethics committee). Please also provide contact information for a data access committee, ethics committee, or other institutional body to which data requests may be sent. If data are owned by a third party, please indicate how others may request data access.

7. Some material included in your submission may be copyrighted. According to PLOS’s copyright policy, authors who use figures or other material (e.g., graphics, clipart, maps) from another author or copyright holder must demonstrate or obtain permission to publish this material under the Creative Commons Attribution 4.0 International (CC BY 4.0) License used by PLOS journals. Please closely review the details of PLOS’s copyright requirements here: PLOS Licenses and Copyright. If you need to request permissions from a copyright holder, you may use PLOS's Copyright Content Permission form.

Please respond directly to this email or email the journal office and provide any known details concerning your material's license terms and permissions required for reuse, even if you have not yet obtained copyright permissions or are unsure of your material's copyright compatibility.

Potential Copyright Issues:

"Graphical Abstract": Please confirm (a) that you are the photographer; or (b) provide written permission from the photographer to publish the photo(s) under our CC-BY 4.0 license.

**Additional Editor Comments (if provided):** **Reviewers' Comments:**

**Comments to the Author**

1. Does this manuscript meet PLOS Digital Health’s publication criteria?

Reviewer #1: Yes

Reviewer #2: Partly

**********

2. Has the statistical analysis been performed appropriately and rigorously?

Reviewer #1: Yes

Reviewer #2: Yes

**********

3. Have the authors made all data underlying the findings in their manuscript fully available (please refer to the Data Availability Statement at the start of the manuscript PDF file)?

The PLOS Data policy

Reviewer #1: No

Reviewer #2: Yes

**********

4. Is the manuscript presented in an intelligible fashion and written in standard English?

Reviewer #1: Yes

Reviewer #2: Yes

**********

**Review Comments to the Author**

Reviewer #1: I have the pleasure to review the article entitled “Agreement and diagnostic accuracy of a computational model of coronary microvascular physiology against continuous thermodilution” by Daniel Taylor and colleagues.

I would like to congratulate the authors about the thorough work carried out in this project.

Overall, the manuscript is well written and addresses an important topic of ongoing research (less invasive diagnostic techniques for assessing CMD).

I have some comments that I think should have to be taken into consideration:

1.In the title and abstract, it is not clear to readers how the computational indices are calculated. In my opinion, mentioning that VirtuQ is based upon computational angiography and FFR is needed.

2.I think CFD-MRR and invasive thermodilution-MRR should be labelled differently to improve clarity and avoid confusion throughout the text (e.g v-MRR for VirtuQ-calculated and t-MRR for continuous thermodilution). Also, for other indices (CFR, absolute flow, etc).

3.In line 111, I think that saying that FFR does not give any information about the microcirculation is not correct. Although is mainly used to assess epicardial CAD, FFR values can also be affected in some degree by downstream coronary microcirculation function, mainly hyperaemic resistance (DOI: 10.1136/heartjnl-2013-305124). So, in my opinion, this statement is too categorical.

4.Was the inclusion of patients done in a prospective fashion or was it a retrospective analysis from a cohort of patients that underwent continuous thermodilution assessment of CMD?

5.Regarding the methods, do the authors included patients with CTOs? In that case, how can they deal with collateral branches arising from interrogated vessels? Was that considered in VirtuQ calculations?

6.In patients where ACS was the clinical presentation, was interrogation done only in non-culprit vessels? How much time passed from the clinical presentation (ACS) until physiological assessment? And, were both measurements (VirtuQ and continuous thermodilution) done at the same time? This issue is important, and I think it should be stressed in the discussion: how the dynamic changes in intracoronary physiology after an ACS may influence a measurement that relies on FFR.

7.Do the authors have an explanation on why computed MRR and computed CFR tend to be lower than continuous thermodilution-based measurements? In the discussion, it is hypothesized that the relatively high proportion of ACS patients may have biased the measurements (by increasing the prevalence of cases with low MRR). However, if both measurements were done at the same time, the transient abnormalities in the microcirculation secondary to a recent ACS should affect both indices.

8.In this study, a high proportion of patients had positive FFR, something that it is not that common in most patients referred for invasive assessment of CMD (e.g. patients without epicardial disease or mild/moderate stenoses). This makes, in my opinion, the results less comparable to everyday practice in most centers. I think it should be stressed in the title and/or abstract that this study was carried out mostly in patients with epicardial CAD.

9.In figure 3 is concerning the drop in classification agreement around the cutoff of 2.5, that should be the critical point where most cases will be. How can the authors explain this?

Reviewer #2: In the current manuscript, the authors aimed to validate a computational model of coronary microvascular physiology in-vivo. They compared 131 arteries from 89 patients with chronic and acute coronary syndromes undergoing continuous thermodilution and angiography-derived assessment by using three-dimensional computational fluid dynamics (3D CFD). The median computed microvascular resistance reserve (MRR) of 2.31 [1.83 – 3.00] was significantly lower than the median thermodilution assessed MRR of 2.79 [2.21 – 3.52] (z = 3.57, p = 0.0004). Moderate relationships were found between both techniques for determining MRR and hyperemic vessel inlet flow and agreement between both techniques improved at lower values.

A few major and minor comments should be addressed to improve the quality of the manuscript.

Major comments:

-There doesn’t seem to be an exaggerated difference between computed MRR and thermodilution assessed MRR. However, in this population, median values were respectively 2.31 and 2.79 for both techniques. With a pre-specified MRR threshold of ≤2.5, there seem to be an impact on classification of patients in normal or abnormal values. There is no clear discussion about that.

-One of the major advantages of CFD highlighted by the authors is to decrease instrumentation and need for invasive measurements. There are 2 remarks on that statement. The first is that one still needs an invasive distal pressure measurement. The second is that microcirculation assessment during ANOCA, NSTEMI and STEMI are debated. In a great number of cases, patients will have to come back a second time to the cathlab for microcirculation assessment in stable conditions. This specific advantage of CFD should discussed in more details.

Minor comments:

-At line 441-442 the author emphasizes that computed CFD may be less accurate in patients with angina with non-obstructive coronary arteries (ANOCA). However, those patients seem to be the main target population. This should be highlighted.

-Producing suitable images for computed analysis includes more contrast use. This is not mentioned in this article. From the total of 178 arteries, only 116 were selected and analyzed, showing that good image quality is needed. This could potentially increase procedural contrast use.

**********

what does this mean?). If published, this will include your full peer review and any attached files.

**Do you want your identity to be public for this peer review?**  If you choose “no”, your identity will remain anonymous but your review may still be made public.

For information about this choice, including consent withdrawal, please see our Privacy Policy

Reviewer #1: **Yes:**  Alejandro Travieso

Reviewer #2: No

**********

**Figure resubmission:**

**Reproducibility:**

To enhance the reproducibility of your results, we recommend that authors of applicable studies deposit laboratory protocols in protocols.io, where a protocol can be assigned its own identifier (DOI) such that it can be cited independently in the future. Additionally, PLOS ONE offers an option to publish peer-reviewed clinical study protocols. Read more information on sharing protocols at https://plos.org/protocols?utm_medium=editorial-email&utm_source=authorletters&utm_campaign=protocols

# Author comment: Author response to Decision Letter 1

**DOI:** 10.1371/journal.pdig.0001830.r002

8 Sep 2026

Attachment Submitted filename: Response to Reviewers.docx

# Peer review: Decision Letter 1

**DOI:** 10.1371/journal.pdig.0001830.r003

**Authors:** Peter H Charlton, Haibo Ni

22 Oct 2026

Agreement of an invasive angiography-derived computational model of coronary microvascular physiology against continuous thermodilution

PDIG-D-26-00495R1

Dear Dr Taylor,

We are pleased to inform you that your manuscript 'Agreement of an invasive angiography-derived computational model of coronary microvascular physiology against continuous thermodilution' has been provisionally accepted for publication in PLOS Digital Health.

Before your manuscript can be formally accepted you will need to complete some formatting changes, which you will receive in a follow-up email from a member of our team.

Please note that your manuscript will not be scheduled for publication until you have made the required changes, so a swift response is appreciated.

IMPORTANT: The editorial review process is now complete. PLOS will only permit corrections to spelling, formatting or significant scientific errors from this point onwards. Requests for major changes, or any which affect the scientific understanding of your work, will cause delays to the publication date of your manuscript.

If your institution or institutions have a press office, please notify them about your upcoming paper to help maximize its impact. If they'll be preparing press materials, please inform our press team as soon as possible -- no later than 48 hours after receiving the formal acceptance. Your manuscript will remain under strict press embargo until 2 pm Eastern Time on the date of publication. For more information, please contact digitalhealth@plos.org.

Thank you again for supporting Open Access publishing; we are looking forward to publishing your work in PLOS Digital Health.

Best regards,

Haibo Ni

Academic Editor

PLOS Digital Health

***********************************************************

**Additional Editor Comments (if provided):**

Both reviewers agree that the revision has improved the manuscript significantly, and raised no further questions.

**Reviewer Comments (if any, and for reference):**

Reviewer's Responses to Questions

**Comments to the Author**

Reviewer #1: All comments have been addressed

Reviewer #2: All comments have been addressed

**********

publication criteria?

Reviewer #1: Yes

Reviewer #2: Yes

**********

3. Has the statistical analysis been performed appropriately and rigorously?

Reviewer #1: Yes

Reviewer #2: Yes

**********

4. Have the authors made all data underlying the findings in their manuscript fully available (please refer to the Data Availability Statement at the start of the manuscript PDF file)?

The PLOS Data policy

Reviewer #1: Yes

Reviewer #2: Yes

**********

5. Is the manuscript presented in an intelligible fashion and written in standard English?

Reviewer #1: Yes

Reviewer #2: Yes

**********

Reviewer #1: I would like to thank the authors for the effort put in improving the manuscript. I think that overall, all my questions have been correctly addressed and responded.

I have some final suggestions:

• Regarding comment 8, I think is a good ideato show the median FFR and % of lesions with ischemic value (below the widespread cutoff of 0.80). However, I think it must be clearer (e.g. in the discussion and conclusions) that these results are mainly driven by lesions with a significant proportion of flow-limiting epicardial CAD. This will clearly aware the readers that the results cannot be directly extrapolated to ANOCA patients.

• I fully agree with your explanation of comment 9. However, in clinical grounds, the median values obtained from virtuQ-MRR and invasive MRR indicate that a significant proportion of patients will be switched from diagnostic category (CMD present or no). It is something inherent to binary classifications, but in a clinical picture it will be much more frequent to deal with a value close to the threshold (e.g MRR of 2.4) than to have an extreme value that, of course, will have better agreement between both indices. I just remark that because the statement made in lines 516-518 seems a bit too optimistic.

Reviewer #2: All comments were answered

**********

what does this mean?). If published, this will include your full peer review and any attached files.

**Do you want your identity to be public for this peer review?**  If you choose “no”, your identity will remain anonymous but your review may still be made public.

For information about this choice, including consent withdrawal, please see our Privacy Policy

Reviewer #1: **Yes:**  Alejandro Travieso

Reviewer #2: No

**********
